# Effects of different exercise-based rehabilitation programs in adults with pulmonary arterial hypertension and related pulmonary hypertension populations: a systematic review and network meta-analysis

**DOI:** 10.1186/s13102-026-01864-w

**Published:** 2026-07-08

**Authors:** Chen Hua, Wenrui Huang, Zhen Chen, Xiaoyang Cai, Lin Tang, Juan Jia, Juan Yang, Chen Zhang

**Affiliations:** 1 The Second Affiliated Hospital Of Army Medical University: Xinqiao Hospital, Chongqing, China; 2 The Fourth Clinical Medical College of Guangzhou University of Chinese Medicine, Shenzhen, Guangdong China; 3 Army Medical University Army Health Service Training Base, Chongqing, China

**Keywords:** Pulmonary arterial hypertension, Aerobic exercise, Resistance exercise, Breathing training, Network meta-analysis, Rehabilitation

## Abstract

**Background:**

Pulmonary arterial hypertension (PAH) and related pulmonary hypertension populations are associated with impaired exercise capacity and reduced quality of life. Supervised exercise rehabilitation is recommended for clinically stable patients, but the relative effects and certainty of evidence for different exercise components and combinations remain uncertain.

**Methods:**

We conducted a systematic review and Bayesian network meta-analysis of randomised controlled trials. The review was initiated on 1 July 2025 and registered in PROSPERO on 18 July 2025. PubMed, Embase, Web of Science, the Cochrane Library, CNKI, VIP and Wanfang were searched from database inception, defined as the earliest records indexed in each database, to 2 June 2026, together with reference lists and trial registries. Eligible interventions were aerobic exercise (AE), resistance exercise (RE), breathing training (BT), aerobic exercise plus resistance exercise (AE + RE), aerobic exercise plus breathing training (AE + BT), and usual care (UC). Analyses used change-from-baseline values when available and post-intervention endpoint values otherwise. Risk of bias was assessed with RoB 2. Network estimates were reported as mean differences (MDs) with 95% credible intervals (CrIs). Certainty of evidence was assessed using the GRADE approach adapted for network meta-analysis and the Confidence in Network Meta-Analysis (CINeMA) framework.

**Results:**

Sixteen trials involving 644 participants were included. For health-related quality of life measured using the Short Form-36 (SF-36), AE + RE showed a favourable but imprecise signal, and the credible interval did not exclude no clear effect. For 6-min walk distance (6MWD), BT showed the largest estimated improvement versus UC; however, this finding was based on limited direct evidence and should be interpreted cautiously. AE + BT improved peak oxygen uptake compared with UC. Evidence for forced expiratory volume in 1 s percentage predicted (FEV_1_%) and N-terminal pro-B-type natriuretic peptide (NT-proBNP) was particularly sparse and imprecise, and no firm conclusions could be drawn for these outcomes. Overall, certainty of evidence was generally low or very low for many active-intervention comparisons because of sparse networks, wide credible intervals, risk of bias and clinical indirectness.

**Conclusions:**

Supervised exercise-based rehabilitation showed favourable signals for selected outcomes assessed mainly at the end of the intervention in adults with PAH and related pulmonary hypertension populations. However, the evidence remains uncertain because several networks were sparse, credible intervals were wide, direct active-versus-active comparisons were limited, and certainty of evidence was generally low or very low. SUCRA rankings should be interpreted only as exploratory signals and not as confirmatory evidence for choosing one exercise modality over another. In particular, the favourable ranking of AE + RE for SF-36 should not be considered clear superiority for quality of life. Larger, adequately powered and subtype-stratified trials with standardised exercise prescriptions, harmonised safety reporting and longer follow-up are needed.

**Trial registration:**

PROSPERO CRD420251107670.

**Supplementary Information:**

The online version contains supplementary material available at https://doi.org/10.1186/s13102-026-01864-w.

## Background

Pulmonary arterial hypertension (PAH) is a progressive and life-threatening pulmonary vascular disease characterised by pulmonary vascular remodelling, persistently elevated pulmonary arterial pressure and increased pulmonary vascular resistance. Progressive right ventricular overload may lead to right heart failure and premature death [1, 2]. Although PAH is uncommon, it creates a substantial burden for patients and health systems. Annual incidence has been estimated at 2–7.6 cases per million, with a prevalence of 15–60 cases per million worldwide [3]. Patients commonly experience exertional dyspnoea, fatigue, exercise intolerance and impaired health-related quality of life [3].

Exercise-based rehabilitation has become an important adjunct to pharmacological treatment for clinically stable patients with PAH. Contemporary guidelines and clinical trials support carefully supervised training in selected patients, with programmes commonly combining aerobic exercise, resistance exercise, and breathing or respiratory muscle training [2, 4–7]. These components target different contributors to exercise limitation, including reduced cardiopulmonary reserve, skeletal muscle dysfunction, abnormal ventilatory efficiency and symptom-related avoidance of activity.

Several systematic reviews and meta-analyses have examined exercise training or rehabilitation in pulmonary hypertension, including the reviews by Zeng et al., Yan et al., Satyamurthy et al. and VanAken et al. [7–10]. These reviews consistently suggest that rehabilitation can improve exercise capacity and may improve quality of life. However, most prior syntheses relied mainly on pairwise comparisons, combined heterogeneous exercise programmes, or focused on the overall effect of exercise versus usual care. They therefore provide limited information on the relative effects of specific supervised exercise components or predefined combinations, which is directly relevant to exercise prescription in rehabilitation practice.

To address this gap, we conducted a systematic review and Bayesian network meta-analysis of randomised controlled trials comparing aerobic exercise (AE), resistance exercise (RE), breathing training (BT), aerobic exercise plus resistance exercise (AE + RE), aerobic exercise plus breathing training (AE + BT), and usual care (UC) in adults with PAH or closely related pulmonary hypertension populations treated in rehabilitation settings. The objective was to estimate the comparative effects, rankings and certainty of evidence for these strategies on health-related quality of life, exercise capacity and selected cardiopulmonary or physiological outcomes.

A network meta-analysis was considered appropriate because the available evidence was clinically structured around several competing rehabilitation strategies, but most trials compared one active strategy with UC rather than directly comparing two active interventions. Therefore, a conventional pairwise meta-analysis would mainly estimate the overall effect of individual interventions versus UC and would provide limited information on how different active rehabilitation strategies compare with one another. By combining direct and indirect evidence within a single coherent framework, NMA allowed simultaneous comparison of predefined exercise nodes and provided an exploratory estimate of which components or combinations appeared most promising. Given the small number of trials, sparse networks and limited active-versus-active evidence, these comparisons and rankings were interpreted cautiously and were not treated as confirmatory evidence of superiority.

## Methods

This systematic review and network meta-analysis followed PRISMA 2020 and the PRISMA extension for network meta-analysis [11, 12]. The review was initiated on 1 July 2025 and registered in PROSPERO on 18 July 2025 (CRD420251107670). A PROSPERO record update was submitted on 21 June 2026 to reflect the completed review stage, including completion of study screening, data extraction, risk-of-bias assessment and data synthesis. During the review process, several methodological refinements were made to improve completeness and transparency without changing the core review question or objective. These refinements included expanding the language criteria from English only to English and Chinese, broadening the database coverage, updating the planned risk-of-bias assessment from RoB 1 to RoB 2, searching trial registries for ongoing or unpublished studies, applying GRADE/CINeMA certainty assessment, and conducting PH subtype sensitivity analyses where feasible. To report these refinements transparently, we added a protocol-modification log specifying the date of each modification and whether it was made before screening, data extraction, primary statistical analysis or during revision. Details are provided in Supplementary Appendix 18, Table S18.1.

### Study design and eligibility criteria

Eligibility criteria were defined using the PICOS framework.

The primary target population was adults aged 18 years or older with pulmonary arterial hypertension (PAH). Because the exercise-rehabilitation literature in this field also includes clinically related pulmonary hypertension populations treated in similar rehabilitation settings, we also considered trials enrolling chronic thromboembolic pulmonary hypertension (CTEPH) or congenital heart disease-associated PAH/PH. These populations were included only when they represented the main study population and when the rehabilitation intervention and outcome assessment were clinically comparable with those used in PAH trials.

Because PAH, CTEPH and congenital heart disease-associated PAH/PH differ in pathophysiology, treatment pathways, baseline functional status and potential response to rehabilitation, pulmonary hypertension subtype was treated as a potential effect modifier and a possible source of clinical indirectness. We therefore classified all studies according to pulmonary hypertension subtype, considered subtype distribution when assessing transitivity and certainty of evidence, and performed PAH-only sensitivity analyses where the network structure allowed meaningful estimation.

Eligible interventions were aerobic exercise (AE), resistance exercise (RE), breathing training (BT), aerobic exercise plus resistance exercise (AE + RE), and aerobic exercise plus breathing training (AE + BT). Control groups received usual care (UC) without a structured exercise programme or, in one active-comparator trial, AE alone. Intervention nodes were defined according to the dominant planned exercise components rather than programme setting, supervision level, frequency, session duration, total programme duration or delivery mode. AE included structured whole-body or limb-based rhythmic exercise intended primarily to improve aerobic capacity, such as walking, cycling, treadmill exercise or comparable low-intensity rhythmic exercise. RE referred to structured strengthening exercise using body weight, external resistance or resistance devices. BT included inspiratory muscle training or structured breathing exercises. Combined nodes were assigned when AE was deliberately combined with BT or RE as a planned component of the intervention. When programmes included additional minor components, such as stretching, flexibility training or chest-expansion exercises, classification was based on the dominant therapeutic components specified in the trial protocol or intervention description. Because several rehabilitation programmes were multicomponent and did not map perfectly onto a single exercise category, node assignment was treated as a pragmatic classification for evidence synthesis rather than as confirmation that all interventions within a node were identical in content, dose or delivery. Details and rationale for intervention-node classification are provided in Supplementary Appendix 17.

We placed no restrictions on programme duration, training frequency, exercise intensity, setting or supervision level, provided the intervention could be assigned to one of the predefined treatment categories. This pragmatic node classification was necessary because exercise-based rehabilitation trials in PAH and related pulmonary hypertension populations are clinically heterogeneous and often combine several exercise components. Therefore, the treatment nodes should be interpreted as broad rehabilitation strategies rather than identical exercise prescriptions.

The primary outcomes were health-related quality of life and exercise capacity. Health-related quality of life was assessed using Short Form-36 (SF-36) scores. For quantitative synthesis, we used only SF-36 overall, total or composite scores that were directly reported by the original trial authors and were presented on a comparable 0–100 scale, with higher scores indicating better health-related quality of life. We did not calculate an overall SF-36 score from the eight individual domain scores, and no formula was applied by the review authors to combine SF-36 domains. Studies reporting only individual SF-36 domains, physical or mental component summaries, or SF-36 results in a format that could not be harmonised with the pooled 0–100 metric were not included in the SF-36 network meta-analysis. Exercise capacity was measured by 6-min walk distance (6MWD). Secondary outcomes included peak oxygen uptake (peak VO_2_), N-terminal pro-B-type natriuretic peptide (NT-proBNP), forced expiratory volume in 1 s percentage predicted (FEV_1_%), resting heart rate, and maximum heart rate.

We included individually randomised parallel-group randomised controlled trials published in English or Chinese. Conference abstracts, editorials, commentaries, reviews, animal studies, observational studies, case reports, and studies with insufficient outcome data were excluded.

### Data sources

The review was initiated on 1 July 2025 and registered in PROSPERO on 18 July 2025 (CRD420251107670). The final updated search was completed on 2 June 2026. We searched PubMed, Embase, Web of Science, the Cochrane Library, CNKI, VIP and Wanfang from database inception, defined as the earliest records indexed in each database, to 2 June 2026. The English search strategy combined controlled vocabulary and free-text terms related to pulmonary arterial hypertension or pulmonary hypertension, exercise or rehabilitation, and randomised controlled trials. The Chinese search strategy used corresponding Chinese terms, including “肺动脉高压”, “肺高血压”, “肺性高血压”, “运动”, “运动训练”, “运动康复”, “康复训练”, “有氧运动”, “抗阻训练”, “呼吸训练”, “呼吸肌训练”, “随机”, “随机对照试验” and “临床试验”.

Reference lists of eligible studies and relevant reviews were also screened. Trial registries, including ClinicalTrials.gov, the WHO International Clinical Trials Registry Platform and the Chinese Clinical Trial Registry, were searched to identify ongoing, unpublished or terminated studies. Full English and Chinese search strategies, including the search terms used for each database and registry, are reported in Supplementary Appendix 1*.*

### Study selection and data extraction

All retrieved records were imported into EndNote, and duplicates were removed before screening. Two reviewers (CH and WRH) independently screened titles and abstracts, followed by full-text assessment of potentially eligible studies. Disagreements were resolved through discussion, with a third reviewer (CZ) consulted when necessary.

For duplicate publications, companion reports or overlapping cohorts, we compared author names, recruitment centres, study periods, sample sizes, intervention descriptions and outcome reporting. When multiple reports described the same or overlapping participant cohort, the most complete report was retained as the primary source, and additional non-overlapping information from companion reports was extracted when relevant. The same participants were not counted more than once in any quantitative synthesis.

Two reviewers (CH and WRH) independently extracted data using a standardised extraction form. The extracted information included study characteristics, participant characteristics, pulmonary hypertension subtype, clinical stability and background therapy when reported, intervention details, control conditions, adverse events, follow-up time points and outcome data. To assess comparability across studies, we also extracted potential effect modifiers where available, including mean age, sex distribution, baseline functional status, pulmonary hypertension subtype, intervention duration, training frequency, supervision setting and baseline 6MWD.

When essential outcome data were missing, unclear or not directly usable, we first checked the full text, tables, figures, supplementary materials, companion publications and available trial registry records. If the required data could not be obtained or derived from these sources, the study was not included in the quantitative synthesis for that specific outcome. Studies were still retained in the systematic review and summarised narratively when they met the eligibility criteria but lacked usable quantitative data for a specific network.

For data reported only in figures, numerical values were extracted using digital plot extraction methods when necessary. Extracted values were checked independently by a second reviewer against the original figure. When uncertainty remained because of low figure resolution, unclear axes or overlapping data points, the extracted data were used only if the values could be read with sufficient confidence; otherwise, the study was not pooled for that specific outcome.

For each outcome, data were extracted at the end of the intervention or at the nearest post-intervention assessment time point. This time point was used because it was the most consistently reported across included trials. Longer-term follow-up data were extracted when available and considered narratively, but they were not sufficiently or consistently reported to support separate long-term network meta-analyses.

### Risk of bias assessment

Two reviewers (CH and ZC) independently assessed risk of bias using the Cochrane Risk of Bias 2 tool [13]. Assessments were performed at the outcome level. The five domains covered bias related to the randomisation process, deviations from intended interventions, missing outcome data, outcome measurement, and selection of the reported result. Each domain and the overall judgement were rated as low risk, some concerns, or high risk. Disagreements were resolved through discussion or consultation with a third reviewer (CZ). In the Results section, study-level summaries are presented only as a descriptive overview of the included trials. The primary RoB 2 assessment was outcome-specific, and the certainty assessment and interpretation of each network outcome were based on the outcome-level RoB 2 judgements reported in the Supplementary Material.

### Statistical analysis

Continuous outcomes were analysed as mean differences with 95% credible intervals when studies reported the same outcome on a comparable scale. For each outcome, change-from-baseline values were prioritised when available because they account for within-study baseline values. When change-from-baseline values were unavailable, post-intervention endpoint values were used if the measurement scale and direction were comparable. We did not combine different measurement scales within the same outcome network. The data type used for each study and each outcome, including change-from-baseline values, post-intervention endpoint values, derived or converted endpoint values, and unavailable study-outcome combinations, is reported in Supplementary Appendix 16, Table S16.1. Because some networks included both change-from-baseline and endpoint values, this issue was considered a potential source of bias or indirectness and was taken into account when interpreting precision, transitivity and certainty of evidence. If standard deviations were not directly reported, they were derived from available summary statistics, such as standard errors, confidence intervals, or p values, following Cochrane Handbook guidance. For studies reporting baseline and endpoint values but not change-score standard deviations, SDs of change were calculated from the reported SDs using an estimated within-group correlation coefficient when necessary. When required data could not be obtained or derived, the study was not included in the quantitative synthesis for that outcome.

We first conducted conventional pairwise meta-analyses for direct comparisons supported by at least two studies. Because direct active-versus-active comparisons were scarce, the network meta-analysis was used to combine direct and indirect evidence across predefined intervention nodes; however, all indirect and ranking-based comparisons were interpreted in light of network sparsity, precision and certainty of evidence. These analyses were performed in Stata 17.0 using inverse-variance random-effects models, with between-study heterogeneity estimated using the DerSimonian-Laird method. We then conducted the network meta-analysis within a Bayesian framework using the gemtc package in R (version 4.2.2) [14]. Stata was also used for supplementary analyses and graphical presentation.

Both fixed-effect and random-effects consistency models were explored, and model fit was compared using the deviance information criterion (DIC). Because all outcomes were continuous, the Bayesian network meta-analysis used a normal likelihood with an identity link. Relative treatment effects were assigned vague normal priors, specified as normal distributions centred at 0 with low precision, corresponding to a large variance. In the random-effects model, the between-study standard deviation was assigned a vague uniform prior. The same likelihood, link function and prior structure were applied across all outcome networks.

Four Markov chains were run for 50,000 iterations each, with the first 20,000 iterations discarded as burn-in and a thinning interval of 10. Convergence and Markov chain Monte Carlo performance were assessed using trace plots, density plots, Gelman-Rubin diagnostics and autocorrelation plots. DIC values, posterior residual deviance, effective number of parameters and heterogeneity estimates are reported in Supplementary Appendix 6, Table S6.2. Trace and density plots are provided in Supplementary Appendix 7, and Gelman-Rubin and autocorrelation diagnostic plots are provided in Supplementary Appendix 8. Because the interventions were expected to vary clinically and model fit was similar between fixed-effect and random-effects models, the random-effects consistency model was used for the main analysis.

We assessed transitivity by explicitly comparing the distribution of prespecified potential effect modifiers across direct treatment comparisons rather than relying on visual inspection of forest plots alone. These potential effect modifiers included pulmonary hypertension subtype, age, sex distribution, WHO functional class, baseline 6MWD, programme duration, training frequency, exercise dose, intervention setting and supervision. The distribution of these variables was summarised by direct comparison to assess whether participants and interventions were sufficiently comparable for indirect comparisons. The transitivity assessment is reported in Supplementary Appendix 20, Table S20.1. Agreement between direct and indirect evidence was evaluated with node-splitting models, and loop-specific inconsistency was assessed when closed loops were present. Heterogeneity was evaluated using tau-squared (tau^2^) [15–17], while also considering the size and precision of the estimates. No eligible multi-arm trials were identified. Publication bias and small-study effects were explored using comparison-adjusted funnel plots and searches of trial registries. Where data were sufficient, meta-regression analyses were performed to assess the possible influence of mean age, exercise frequency, intervention duration and baseline 6MWD on the primary outcomes. Detailed pairwise forest plots are provided in Supplementary Appendix 5 (Figures S5.1-S5.7).

### Certainty of evidence assessment

For each outcome and key network comparison, certainty of evidence was assessed using the GRADE approach adapted for network meta-analysis and the Confidence in Network Meta-Analysis (CINeMA) framework [18–20]. We evaluated six domains: within-study bias, reporting bias, indirectness, imprecision, heterogeneity and incoherence. Within-study bias was informed by the outcome-level RoB 2 assessment. Reporting bias was assessed by considering the completeness of outcome reporting, comparison-adjusted funnel plots where feasible and trial-registry searches. Indirectness was judged by considering clinical and methodological differences across populations, intervention nodes, comparators, outcome measurements and the distribution of potential effect modifiers. Imprecision was assessed according to the width of credible intervals and whether the intervals included clinically important benefit, no effect or potential harm. Heterogeneity and incoherence were considered using heterogeneity estimates, node-splitting results, loop-specific inconsistency assessment where available and the structure of direct and indirect evidence.

Certainty of evidence was classified as high, moderate, low or very low. Ranking probabilities and the surface under the cumulative ranking curve (SUCRA) were interpreted alongside effect estimates and certainty judgements and were not used as substitutes for certainty assessment. Detailed certainty judgements and downgrading reasons are provided in Supplementary Appendix 13, Table S13.1.

### Sensitivity analysis by pulmonary hypertension subtype

Because the included evidence combined PAH and related pulmonary hypertension populations, including mixed PAH/CTEPH cohorts, CTEPH-only cohorts and congenital heart disease-associated PAH/PH cohorts, pulmonary hypertension subtype was considered a potential clinical effect modifier. We therefore conducted sensitivity analyses according to pulmonary hypertension population where the available network structure allowed meaningful estimation.

The primary sensitivity analysis was restricted to PAH-only trials. Trials enrolling mixed PAH/CTEPH populations, CTEPH-only populations, or congenital heart disease-associated PAH/PH populations were excluded from this analysis. The PAH-only sensitivity analysis was performed only for outcomes in which the remaining treatment network was connected and contained sufficient data to estimate relative effects. When subtype restriction disconnected the network, left an intervention node supported by only one very small comparison, or produced unstable estimates, a separate network meta-analysis was not performed.

Separate quantitative network meta-analyses for CTEPH-only and congenital heart disease-associated PAH/PH populations were not considered statistically reliable because these subgroups were represented by very few trials and did not form connected outcome-specific networks. These studies were therefore summarised narratively and compared with the direction of the primary analysis where possible. Details of pulmonary hypertension subtype classification and sensitivity-analysis feasibility are provided in Supplementary Appendix 14, Table S14.1.

## Results

### Study selection

**Fig. 1:**
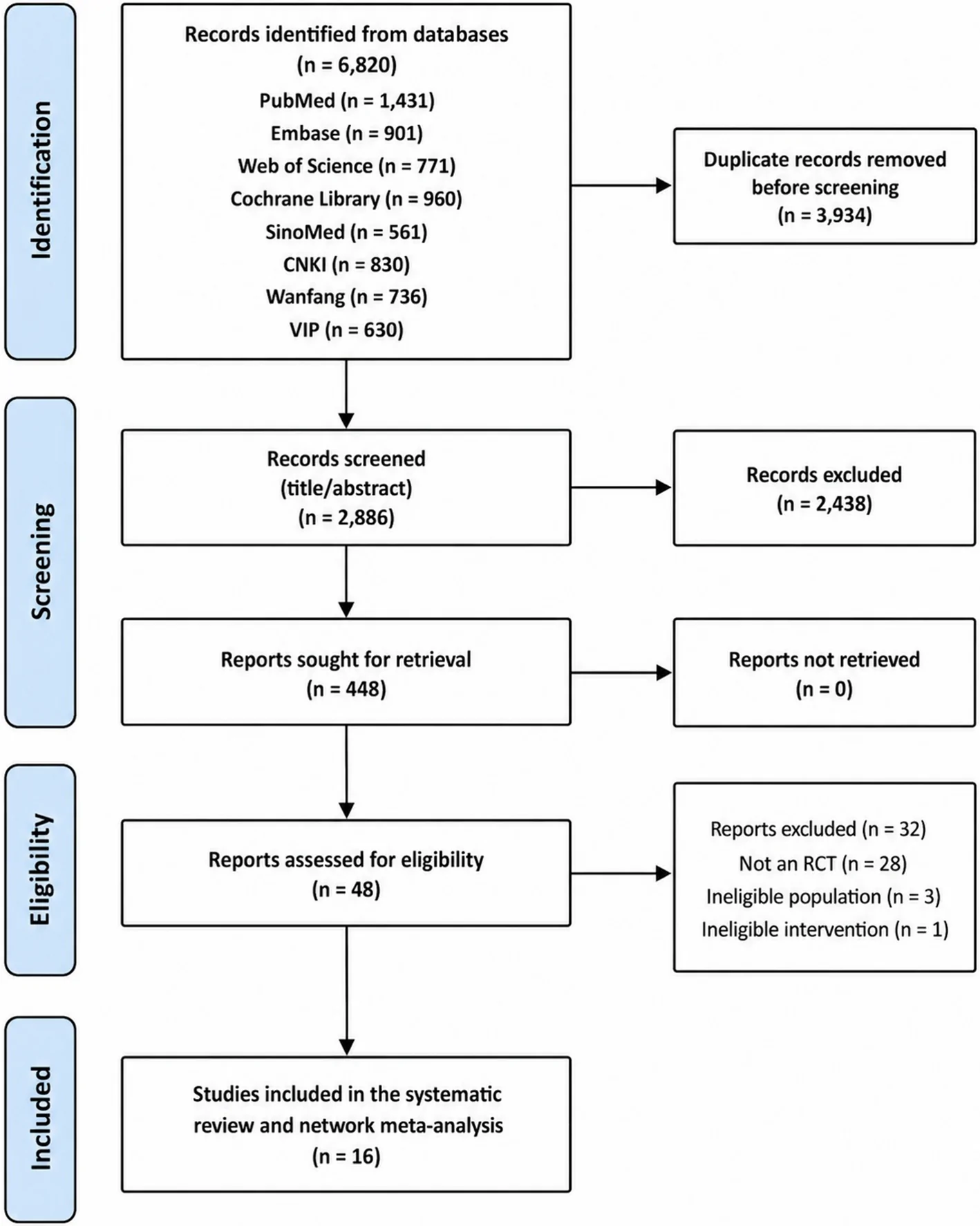
PRISMA 2020 flow diagram of study selection

We identified 6,820 records. After duplicates were removed, 2,886 records were screened by title and abstract, and 48 full-text reports were assessed for eligibility. Sixteen randomised controlled trials involving 644 participants were included in the final analysis [21–36]. The study selection process is shown in Fig. 1. Studies excluded after full-text review and the main reasons for exclusion are listed in Appendix 2, Table S2.1.

### Study characteristics

The 16 included RCTs [21–36] involved 644 participants and were conducted in China, Germany, the United States, Spain, Turkey, Greece, Indonesia, Australia and India. Thirteen studies were single-centre trials and three were multicentre trials. Regarding pulmonary hypertension subtype, 10 trials enrolled PAH-only cohorts, three enrolled mixed PAH/CTEPH cohorts, one enrolled a CTEPH-only cohort and two enrolled congenital heart disease-associated PAH/PH populations. Reported baseline disease severity varied across studies, but most trials enrolled clinically stable patients with WHO functional class II–III disease; a smaller number included patients with WHO functional class I or IV. Mean participant age ranged from 22.7 to 66.0 years. Women were the majority overall, and among studies reporting sex distribution, the proportion of men ranged from 0% to 62.5%.

Clinical stability and background therapy were reported inconsistently. Where reported, participants were described as clinically stable, and several trials required stable disease-targeted or background medical therapy before enrolment or no planned medication changes during the intervention period. However, the duration and definition of treatment stability were not reported uniformly across all trials. These differences in pulmonary hypertension subtype, baseline disease severity, clinical stability and background therapy were considered when assessing clinical comparability, transitivity, indirectness and certainty of evidence.

The most common direct comparisons were AE versus UC (six studies) and AE + BT versus UC (five studies). Other direct comparisons included BT versus UC (two studies), AE + RE versus UC (two studies) and AE + BT versus AE (one study). Direct active-versus-active evidence was therefore limited to one trial, and most active comparisons in the network relied partly on indirect evidence. Full study and baseline characteristics, including pulmonary hypertension subtype, baseline functional status, clinical stability, background therapy and inclusion in the PAH-only sensitivity analysis, are presented in Supplementary Appendix 3, Table S3.1.

### Intervention characteristics

The included rehabilitation programmes were grouped according to the predefined intervention nodes: aerobic exercise (AE), resistance exercise (RE), breathing training (BT), aerobic exercise plus resistance exercise (AE + RE), and aerobic exercise plus breathing training (AE + BT). The control node was usual care (UC). Intervention nodes were assigned according to the dominant planned exercise components rather than programme setting, supervision level, frequency, session duration, total programme duration or delivery mode. Therefore, these nodes should be interpreted as pragmatic rehabilitation categories rather than identical exercise prescriptions.

Exercise dose and delivery varied substantially across studies and intervention nodes. Overall, programme duration ranged from 6 weeks to 6 months, training frequency ranged from 2 to 7 sessions per week, and session duration ranged from approximately 20 to 60 min. The AE node included walking-based activity, treadmill walking, cycle ergometer training, upper-extremity aerobic exercise, Wuqinxi bird-play exercise and walking/cycle-based programmes. These interventions differed in intensity, supervision and setting, ranging from hospital- or outpatient-supervised training to home-based or remotely supported physical-activity programmes.

The BT node mainly consisted of inspiratory muscle training or structured breathing exercises, commonly delivered five to seven times per week for approximately 20 to 30 min per session, when reported. The AE + BT node included programmes combining aerobic or whole-body movement components with breathing or respiratory training. These programmes included supervised rehabilitation followed by home continuation, hospital-based aerobic exercise plus inspiratory muscle training, breathing exercises combined with whole-body breathing-related movement, and home-based exercise programmes with aerobic and breathing components. The AE + RE node included programmes combining aerobic training with resistance or strengthening exercises, but the included studies varied in programme duration, setting, supervision and additional components.

Some interventions required pragmatic classification because they did not fully correspond to conventional exercise categories. Wuqinxi bird-play exercise was classified as AE because it was a structured, low-intensity, rhythmic whole-body exercise programme intended to improve functional capacity, although it should not be considered identical to conventional treadmill or cycle-based aerobic training. Jia 2019 [22] was classified as AE + BT because the intervention combined breathing exercises with coordinated whole-body breathing-related movement; however, this classification reflects the dominant breathing-related and whole-body movement components rather than equivalence to standard aerobic training plus inspiratory muscle training. Kagioglou 2021 [32] was classified as AE + RE because aerobic exercise and resistance exercise were the dominant planned components, although the programme also included stretching and breathing/chest-expansion exercises. Karapolat 2019 [34] was treated as an active-comparator trial because the experimental intervention combined aerobic exercise and breathing exercises, whereas the comparator received aerobic exercise alone.

Because exercise frequency, session duration, programme duration, intensity, setting, supervision, progression criteria, adherence support and co-interventions varied across trials, differences between treatment nodes may reflect not only exercise modality but also dose and delivery characteristics. These factors were therefore considered when assessing transitivity, heterogeneity, indirectness and certainty of evidence. Detailed intervention dose and delivery characteristics are reported in Supplementary Appendix 3, Table S3.2, and the rationale for intervention-node classification is provided in Supplementary Appendix 17.

### Risk of bias of included studies

Risk of bias was assessed at the outcome level using the Cochrane RoB 2 tool. Because several trials contributed data to more than one outcome, risk-of-bias judgements could differ across outcomes within the same study, particularly for subjective outcomes such as SF-36 and more objective outcomes such as 6MWD, peak VO_2_ or physiological measures. Therefore, the study-level summary is presented only as a descriptive overview and was not used as a substitute for outcome-level assessment.

Across the included trials, the most common concerns related to the randomisation process, selection of the reported result, and outcome measurement. For self-reported outcomes such as SF-36, lack of blinding in exercise-rehabilitation trials may have increased the risk of measurement bias. For objective or performance-based outcomes, such as 6MWD and peak VO_2_, outcome measurement concerns were generally interpreted in relation to assessor blinding, standardisation of testing procedures and completeness of outcome data.

The outcome-level RoB 2 judgements were used in the GRADE/CINeMA certainty assessment for each network comparison. Complete outcome-level RoB 2 judgements are provided in Supplementary Appendix 4, Table S4.1. A study-level overview is provided only for descriptive purposes in Supplementary Appendix 3, Table S3.3.

### Assessment of transitivity, consistency, and heterogeneity

Assessment of transitivity was based on an explicit comparison of prespecified potential effect modifiers across direct treatment comparisons. These variables included pulmonary hypertension subtype, age, sex distribution, WHO functional class, baseline 6MWD, programme duration, training frequency, intervention setting and supervision. Overall, no single effect modifier showed an extreme imbalance that clearly precluded synthesis; however, clinical heterogeneity was present, particularly for pulmonary hypertension subtype, intervention dose, setting and supervision. These differences were considered when judging indirectness and certainty of evidence. The comparison of effect modifiers across direct treatment comparisons is summarised in Supplementary Appendix 20, Table S20.1. For outcomes with enough direct evidence, node-splitting and loop-specific analyses did not show clear disagreement between direct and indirect evidence. Overall statistical heterogeneity was low to moderate, but this judgement was interpreted together with the sparse number of studies and the width of the credible intervals. Detailed pairwise forest plots, inconsistency tests, model fit results and transitivity summaries are presented in Supplementary Appendix 5 (Figures S5.1-S5.7), Supplementary Appendix 3, Table S3.4, and Supplementary Appendix 6, Table S6.2.

### Network geometry

Networks were available for all primary outcomes and several secondary outcomes. The SF-36 network included six studies with 248 participants, the 6MWD network included 13 studies with 495 participants, the NT-proBNP network included five studies with 232 participants, the peak VO_2_ network included nine studies with 295 participants, the FEV_1_% network included four studies with 113 participants, the resting heart rate network included seven studies with 240 participants, and the maximum heart rate network included eight studies with 256 participants.

The distribution of direct evidence was uneven across comparisons. Across the full set of included trials, most direct evidence compared an active rehabilitation strategy with UC. The most common direct comparisons were AE versus UC and AE + BT versus UC. Smaller amounts of direct evidence were available for BT versus UC and AE + RE versus UC. Direct active-versus-active evidence was available only for AE + BT versus AE, which was contributed by one trial. Therefore, most active-versus-active comparisons in the network, such as BT versus AE, AE + RE versus AE, AE + BT versus AE + RE and BT versus AE + BT, were estimated mainly or entirely through indirect evidence.

**Fig. 2:**
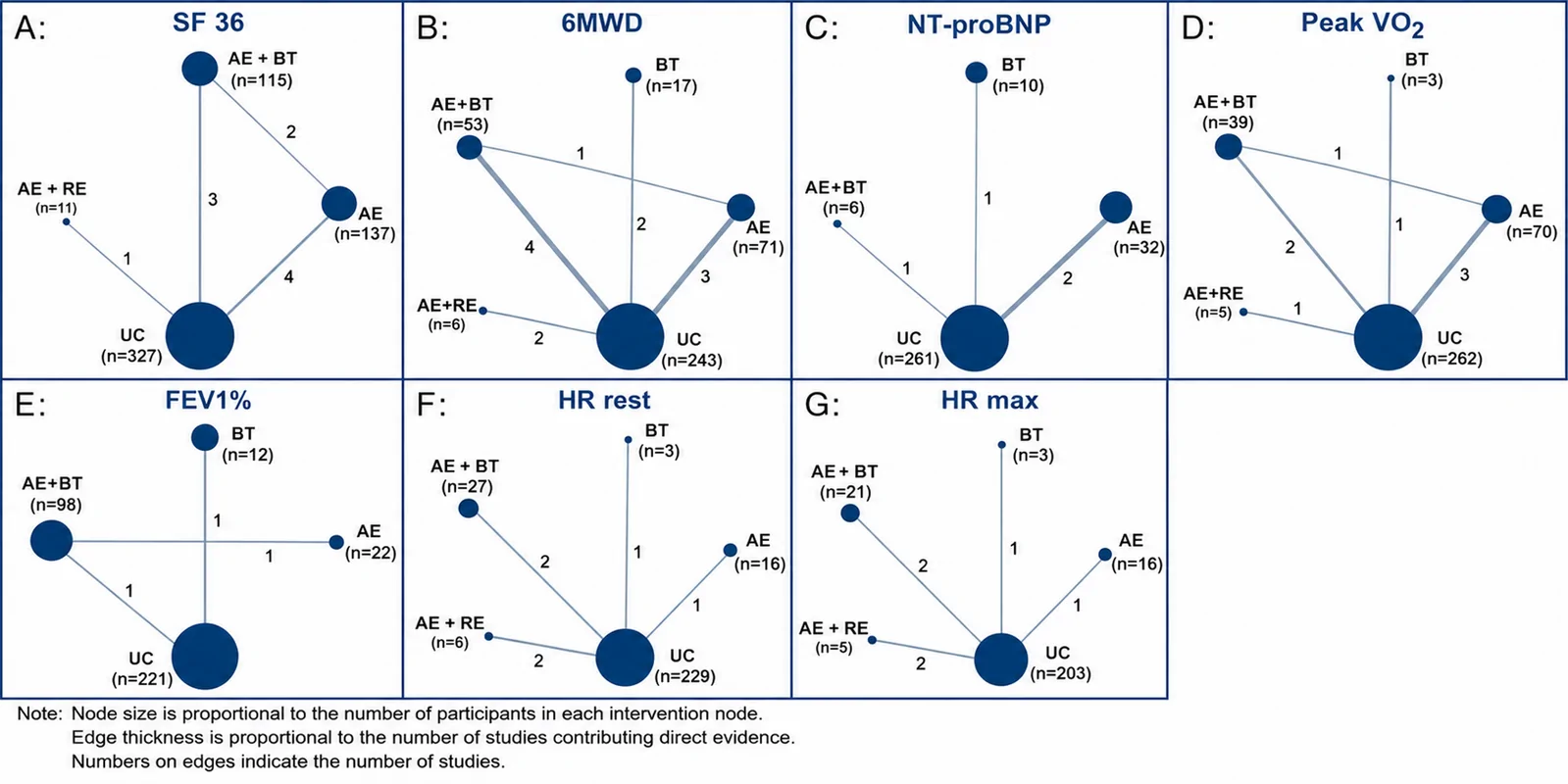
Network plots for exercise-based rehabilitation interventions across outcomes

This evidence structure has important implications for interpretation. Although the network allowed simultaneous comparison of several predefined rehabilitation strategies, the evidence base was largely star-shaped around UC and contained very limited direct evidence comparing two active interventions. Consequently, treatment rankings and active-versus-active estimates were more dependent on indirect comparisons and should be interpreted cautiously, particularly for sparse networks such as NT-proBNP, FEV_1_%, resting heart rate and maximum heart rate. Representative network plots are shown in Fig. 2. In these plots, node size reflects the number of participants, edge thickness reflects the number of studies contributing direct evidence, and edge labels show the number of studies for each direct comparison.

### Loop inconsistency assessment

Node-splitting analyses did not suggest meaningful disagreement between direct and indirect evidence for the outcomes examined. In loops that could be formally assessed, inconsistency factors were small and their 95% confidence intervals included zero, supporting the use of consistency models in the main analyses (Appendix 4, Table S4.4).

### Network meta-analysis

Across all network meta-analyses, SUCRA rankings were interpreted together with the corresponding effect estimates, credible intervals, network geometry and certainty-of-evidence assessment. Because several networks contained few studies and few direct active-versus-active comparisons, high ranking probabilities were not considered sufficient evidence of clinical superiority. Findings from sparse networks were therefore treated as exploratory and hypothesis-generating.

#### SF-36

The SF-36 network included six studies [21, 26, 28, 31, 34, 36] involving 248 participants (Fig. 2A). AE + RE had the highest ranking probability (SUCRA 76.4%), followed by AE (73.8%) (Appendix 11, Figure S11.1; Table S11.1). Compared with UC, AE + RE showed the most favourable point estimate (MD 14.65, 95% CrI −5.28 to 34.24), although the interval was wide. Among active interventions, AE + RE also tended to rank above AE and AE + BT, but these comparisons were imprecise (Appendix 10, Table S10.1).

#### 6MWD

Thirteen studies [21, 22, 24–32, 34, 36] involving 495 participants contributed to the 6MWD network (Fig. 2B). Compared with UC, BT showed the largest estimated improvement (MD 101.72 m, 95% CrI 43.65 to 166.28) and had the highest SUCRA value (83.8%) (Appendix 11, Figure S11.2; Table S11.2). AE and AE + BT also showed favourable point estimates versus UC, both around 39 m. AE + RE showed a larger but less precise estimate (MD 69.09 m, 95% CrI −25.03 to 153.20). BT ranked above AE, AE + BT, and AE + RE, although this result was based on limited direct evidence (Appendix 10, Table S10.2).

#### NT-proBNP

The NT-proBNP network included five studies [21, 24, 26, 27, 33] involving 232 participants (Fig. 2C). No intervention showed clear evidence of benefit, and all estimates were highly uncertain (Appendix 11, Figure S11.3; Table S11.3). For example, the MD for AE versus UC was −64.27 pg/mL (95% CrI −664.37 to 386.38), and the estimate for AE + BT versus UC was 24.43 pg/mL (95% CrI −819.04 to 873.75). SUCRA values were closely grouped, suggesting little separation between interventions (Appendix 10, Table S10.3). Because this network was based on few studies and the credible intervals were very wide, the analysis was underpowered to detect modest biomarker changes. The absence of a clear effect on NT-proBNP should therefore be interpreted as inconclusive rather than as evidence that exercise rehabilitation has no effect on cardiac stress or right ventricular adaptation.

#### Peak VO2

Nine studies [23–25, 27, 31, 32, 34–36] involving 295 participants provided data for the peak VO_2_ network (Fig. 2D). Compared with UC, AE + BT showed the most favourable estimate (MD 4.15 mL·kg^−1^·min^−1^, 95% CrI 1.66 to 6.51) and ranked highest (SUCRA 73.1%), followed by AE + RE (72.1%) and AE (67.2%) (Appendix 11, Figure S11.4; Table S11.4). Although the credible intervals overlapped, combined training strategies generally performed better than single-modality interventions in relative comparisons (Appendix 10, Table S10.4).

#### FEV1%

Four studies [22, 30, 34, 35] involving 113 participants were included in the FEV_1_% network (Fig. 2E). AE + BT ranked highest (SUCRA 80.8%), but the estimate versus UC was imprecise (MD 5.42, 95% CrI −13.83 to 24.53). AE also ranked relatively highly (SUCRA 66.0%) (Appendix 11, Figure S11.5; Table S11.5). Overall, the available evidence did not show a clear short-term effect on FEV_1_% (Appendix 10, Table S10.5). Given the very small number of studies and participants, the FEV_1_% findings should be considered exploratory. The ranking of AE + BT may indicate a potentially promising signal but does not provide confirmatory evidence of superiority.

#### Resting heart rate

Seven studies [24, 26–30, 32] involving 240 participants contributed to the resting heart rate network (Fig. 2F). No intervention showed a clear advantage over UC or other active strategies. AE + RE and AE had the highest SUCRA values (76.0% and 75.2%, respectively) (Appendix 11, Figure S11.6; Table S11.6), but the estimates were imprecise (Appendix 10, Table S10.6).

#### Maximum heart rate

Eight studies [23, 24, 27–29, 31, 33, 35] involving 256 participants formed the maximum heart rate network (Fig. 2G). BT ranked highest (SUCRA 75.8%), followed by AE + BT (71.4%) (Appendix 11, Figure S11.7; Table S11.7), but the estimates were too imprecise to support firm conclusions. Overall, the evidence for heart-rate outcomes was limited and uncertain (Appendix 10, Table S10.7).

### Publication bias assessment

Comparison-adjusted funnel plots were broadly symmetrical for most outcomes (Appendix 9, Figures S9.1–S9.7), although the small number of studies per comparison limits interpretation. Slight asymmetry was seen for NT-proBNP (Appendix 9, Figure S9.3) and resting heart rate (Appendix 9, Figure S9.6), but this may reflect sparse data and small-study effects rather than clear publication bias.

### Meta-regression analysis

Where data were sufficient, we performed exploratory meta-regression analyses to assess whether study-level covariates modified treatment effects. The covariates examined included mean age, intervention duration, exercise frequency and baseline 6MWD, depending on data availability for each outcome. Because few studies were available within each network, all meta-regression analyses were considered exploratory and were not used to make confirmatory subgroup claims.

Overall, the meta-regression analyses did not identify a consistent pattern of effect modification. For SF-36, the coefficients for exercise frequency, intervention duration and mean age were imprecise, and the corresponding credible intervals crossed the null value. For peak VO_2_ and other outcomes with sufficient data, the direction and magnitude of the covariate effects were also inconsistent across models, with wide credible intervals. These findings suggest that the available study-level data were insufficient to determine whether differences in age, programme duration, exercise frequency or baseline functional capacity explained the observed differences between intervention nodes. Detailed meta-regression results are provided in Supplementary Appendix 12.

### Adverse events and safety reporting

Adverse-event reporting was incomplete and heterogeneous across the included trials. Among studies that explicitly reported safety outcomes, no clear signal of serious exercise-related harm was identified. However, adverse events were not uniformly defined, actively monitored, or reported across studies, and the available sample size was too small to support a reliable pooled safety analysis. Therefore, the apparent short-term safety of supervised rehabilitation should be interpreted cautiously and in the context of clinical stability, individualized risk assessment, and professional supervision. A study-level summary of adverse-event reporting is provided in Supplementary Appendix 15, Table S15.1.

### Sensitivity analysis by pulmonary hypertension subtype

Among the 16 included trials, 10 enrolled PAH-only cohorts, three enrolled mixed PAH/CTEPH cohorts, one enrolled a CTEPH-only cohort, and two enrolled congenital heart disease-associated PAH/PH populations. When analyses were restricted to PAH-only cohorts, the overall direction of treatment effects for the better-connected outcomes, particularly 6MWD and peak VO_2_, remained broadly consistent with the primary analysis and continued to favour supervised exercise-based rehabilitation over UC. However, the PAH-only networks became substantially sparser after excluding mixed PAH/CTEPH, CTEPH-only and congenital heart disease-associated PAH/PH cohorts, which reduced precision and affected several intervention nodes, particularly BT and AE + RE.

For SF-36, NT-proBNP, FEV_1_%, resting heart rate and maximum heart rate, subtype-restricted networks were either disconnected, very sparse, or based on too few studies to support reliable separate network estimates. Separate CTEPH-only and congenital heart disease-associated PAH/PH network meta-analyses were not feasible because these populations were represented by very few trials and did not form connected outcome-specific networks. Therefore, the subtype sensitivity analyses did not provide confirmatory evidence that any exercise modality is consistently superior across clinically distinct pulmonary hypertension populations. Overall, these analyses support cautious interpretation of the primary rankings and indicate that the findings are most directly applicable to PAH-only or predominantly PAH populations. Detailed subtype classification and feasibility of sensitivity analyses are summarised in Supplementary Appendix 14, Table S14.1.

### Certainty of evidence

The formal GRADE/CINeMA assessment showed that certainty of evidence was generally limited across outcomes and comparisons. Most active-intervention comparisons were rated as low or very low certainty. The main reasons for downgrading were risk of bias, sparse networks, wide credible intervals, clinical indirectness, heterogeneity and limited direct active-versus-active evidence. For SF-36, 6MWD and peak VO_2_, some interventions showed favourable point estimates or rankings, but certainty was reduced because several estimates were imprecise and many comparisons relied partly on indirect evidence. For NT-proBNP, FEV_1_%, resting heart rate and maximum heart rate, certainty was particularly limited because the corresponding networks included few studies and participants. Overall, the certainty assessment supported interpreting SUCRA rankings as exploratory and hypothesis-generating rather than confirmatory evidence of superiority. The detailed GRADE/CINeMA assessment is shown in Supplementary Appendix 13, Table S13.1.

## Discussion

This Bayesian network meta-analysis compared five predefined supervised exercise strategies in adults with PAH and related pulmonary hypertension populations treated in rehabilitation settings. Across outcomes assessed mainly at the end of the intervention or shortly after intervention completion, AE + RE ranked favourably for health-related quality of life, BT showed the largest point estimate for improvement in 6MWD, and AE + BT produced the most favourable estimate for peak VO_2_. In contrast, effects on FEV_1_% were small and uncertain, and no exercise strategy showed clear benefit for NT-proBNP. However, these findings should be interpreted cautiously. Although the review included 16 RCTs and 644 participants overall, several outcome networks contained few studies and participants, and many active-intervention comparisons were supported mainly by indirect evidence. In addition, the certainty of evidence was generally low or very low for many active-intervention comparisons. Therefore, the observed rankings should be viewed as exploratory and hypothesis-generating rather than as confirmatory evidence that one exercise modality is superior to another.

The favourable ranking of BT for 6MWD also requires cautious interpretation. A physiological explanation is possible: breathing training may improve walking performance by strengthening inspiratory muscles, reducing the work of breathing and easing breathlessness during activity [5, 6]. However, methodological explanations should also be considered. The BT estimate was based on a sparse network with a small number of participants and limited direct evidence, making the ranking vulnerable to small-study effects and statistical instability. In addition, differences in control conditions, exercise dose, supervision, setting and possible co-interventions may have contributed to the observed effect. Because most active-versus-active comparisons involving BT relied largely on indirect evidence, the high ranking of BT for 6MWD should be interpreted as exploratory and hypothesis-generating rather than as confirmatory evidence that BT is superior to other exercise strategies. Larger trials with standardised control conditions, clearly reported co-interventions and direct comparisons between active rehabilitation strategies are needed to confirm this finding.

The SF-36 finding requires particular caution. Although AE + RE had the highest ranking probability for SF-36, the estimate versus UC was imprecise and the credible interval included no clear effect. This result should therefore be viewed as a hypothesis-generating signal for further evaluation of combined aerobic and resistance exercise, rather than evidence that AE + RE is clearly superior for quality of life.

The possible benefits of combined training are biologically plausible, because exercise limitation in PAH may involve several interacting mechanisms, including reduced cardiopulmonary reserve, right ventricular strain, skeletal muscle dysfunction, abnormal ventilatory efficiency and symptom-related avoidance of activity [2, 4, 6]. Combined programmes that include aerobic, resistance or breathing components may therefore address multiple contributors to functional limitation, and previous reviews have suggested that multicomponent rehabilitation may improve exercise capacity and cardiopulmonary outcomes in pulmonary hypertension populations [6, 9]. However, the present network meta-analysis cannot establish mechanistic superiority of any combined programme. Several estimates were imprecise, credible intervals overlapped, and many active-intervention comparisons relied partly or largely on indirect evidence. In addition, the intervention nodes were broad pragmatic categories, and programmes within the same node differed in exercise dose, intensity, frequency, supervision, setting, progression, adherence support and co-interventions. Therefore, mechanistic explanations should be considered hypothesis-generating only, and the observed rankings should not be interpreted as proof that one combined training strategy is mechanistically or clinically superior to another.

We did not find clear benefit of any exercise strategy on NT-proBNP, and this result is also informative. It may indicate that short-term rehabilitation is more likely to improve peripheral function, exercise capacity, and patient-reported outcomes before producing measurable changes in markers of cardiac stress. It is also possible that small sample sizes, limited statistical power, and differences between studies made it difficult to detect modest changes. Therefore, the lack of a clear effect on NT-proBNP should not be taken to mean that exercise rehabilitation has no effect on right heart function or adaptation to exercise. Instead, it may suggest that biomarker outcomes require longer interventions, larger samples, and more consistent study designs. Current guidelines and large training studies emphasise that biomarkers should be interpreted together with exercise capacity, functional class, and patient-reported outcomes rather than used alone to judge treatment value [2, 4]. Future studies should therefore examine the longer-term effects of rehabilitation on right ventricular function, biomarkers, and clinical outcomes.

Quality of life was mainly assessed using the generic SF-36 scale. Although SF-36 captures overall changes in health-related quality of life, it may not fully reflect the symptom burden and disease-specific impact of PAH [3]. Therefore, while AE + RE appeared more favourable on SF-36, the clinical meaning of this finding should be interpreted with the limitations of the instrument in mind.

From a clinical perspective, our findings support supervised, individualised and multicomponent rehabilitation rather than treating any single exercise mode as clearly superior. Current guidelines recommend structured rehabilitation for stable patients with PAH when supported by appropriate risk assessment and professional supervision, with exercise prescription tailored to disease stability, functional status and individual tolerance [2]. Recent reviews likewise support the overall benefit of rehabilitation but show that evidence on the best training dose, component mix, safety reporting and long-term effects remains limited [6, 7, 9]. Future research should include adequately powered PAH-only and subtype-stratified randomised trials, standardised exercise prescriptions, harmonised adverse-event definitions, PAH-specific patient-reported outcomes, objective exercise measures, right ventricular imaging or biomarker endpoints, and longer-term clinical event follow-up.

### Strengths and limitations

This study has several strengths. By including only randomised controlled trials and using a Bayesian network meta-analysis, we were able to combine direct and indirect evidence in a single framework and compare several predefined supervised rehabilitation strategies. This offers a broader evidence base than conventional pairwise meta-analysis alone. The outcomes were clinically relevant and covered multiple dimensions, including quality of life, exercise capacity, and physiological measures such as peak VO_2_, NT-proBNP, FEV_1_%, resting heart rate, and maximum heart rate. In addition, the study followed a prospectively registered protocol and was reported in line with PRISMA 2020 and the network meta-analysis extension. Risk of bias was assessed with RoB 2, and certainty of evidence was assessed using the GRADE approach adapted for network meta-analysis and the CINeMA framework. Finally, by distinguishing single-modality from combined interventions, the findings are easier to interpret in clinical rehabilitation practice and may inform individualised exercise prescription for clinically stable patients with PAH and related pulmonary hypertension populations.

Several limitations should also be considered. First, a key limitation is limited statistical power at the outcome-network level. Although the review included 16 RCTs and 644 participants overall, this total sample size may overstate the amount of evidence available for individual outcomes, because each network included only a subset of trials. This was particularly important for FEV_1_%, NT-proBNP and heart-rate outcomes, for which few studies and wide credible intervals limited the ability to detect modest effects. In addition, direct active-versus-active comparisons were scarce, so several relative effects between exercise strategies relied partly or largely on indirect evidence. These limitations reduce the certainty of the findings and support interpreting many comparisons as exploratory rather than confirmatory. In addition, some outcome networks included a mixture of change-from-baseline values and post-intervention endpoint values because not all trials reported change data. Although change-from-baseline values were prioritised and the data type used for each study-outcome combination was reported transparently, mixing data types may have introduced bias when baseline values differed across studies. Second, most included studies were single-centre trials with short follow-up, which further limited the precision and generalisability of the findings. Third, there was substantial variation across studies in training setting, supervision, frequency, intensity, session length and programme duration, which may have affected comparability between intervention groups. Although intervention nodes were defined according to the dominant planned exercise components, programmes within the same node were not identical exercise prescriptions. Differences in dose, supervision, setting, progression, adherence support and co-interventions may have influenced treatment effects independently of exercise modality. Accordingly, treatment-node rankings should be interpreted as comparisons of broad rehabilitation strategies rather than as evidence for the superiority of a specific exercise prescription, dose, setting or supervision model. Fourth, the inclusion of related pulmonary hypertension populations, including mixed PAH/CTEPH cohorts, CTEPH-only cohorts and congenital heart disease-associated PAH/PH cohorts, may have affected clinical homogeneity and transitivity. Although these populations are clinically relevant to exercise rehabilitation research, they differ in pathophysiology, treatment pathways, baseline functional status and potentially in their response to rehabilitation.

PAH-only sensitivity analyses were therefore performed where networks remained connected. However, several subtype-specific networks became sparse or disconnected after excluding non-PAH or mixed populations. As a result, reliable separate estimates for CTEPH-only and congenital heart disease-associated PAH/PH populations could not be generated, and the primary findings should not be assumed to apply equally across all pulmonary hypertension subtypes. Fifth, although the registered review question was not restricted to short-term effects, the included trials mainly reported outcomes at the end of the intervention or shortly after intervention completion. Therefore, the present network meta-analysis primarily reflects end-of-intervention effects and cannot determine whether the observed benefits are sustained over longer follow-up. Finally, adverse events were incompletely and inconsistently reported across the included trials, which limits firm conclusions about the comparative safety of different exercise strategies. These questions require larger, adequately powered, subtype-stratified randomised trials with standardised exercise prescriptions, harmonised safety reporting and longer follow-up.

## Conclusion

Supervised exercise-based rehabilitation showed potentially favourable effects on selected outcomes assessed mainly at the end of the intervention in adults with PAH and related pulmonary hypertension populations. However, the evidence remains uncertain because several outcome networks were sparse, credible intervals were wide, direct active-versus-active comparisons were limited, and certainty of evidence was generally low or very low. Some intervention nodes ranked favourably for specific outcomes, including AE + RE for SF-36, BT for 6MWD and AE + BT for peak VO_2_, but these rankings should be interpreted as exploratory signals rather than confirmatory evidence of superiority. In particular, the favourable ranking of AE + RE for SF-36 should not be interpreted as clear superiority for quality of life. Because the evidence combined PAH-only trials with a small number of mixed PAH/CTEPH, CTEPH-only and congenital heart disease-associated PAH/PH studies, the applicability of the findings across pulmonary hypertension subtypes remains uncertain. Larger, adequately powered, subtype-stratified randomised trials with standardised exercise prescriptions, harmonised adverse-event reporting and longer follow-up are needed before firm recommendations can be made regarding the optimal exercise modality for specific pulmonary hypertension populations.

## Supplementary Information

Supplementary Material 1.

Supplementary Material 2.

## Data Availability

All data relevant to this study are included in the article and its supplementary information. No additional unpublished data are available. Patient and Public Involvement statement: Patients and/or the public were not involved in the design, conduct, reporting, or dissemination plans of this research.
