# Supplementary material for: Effects of different exercise-based rehabilitation programs in adults with pulmonary arterial hypertension and related pulmonary hypertension populations: a systematic review and network meta-analysis
Source: BMC Sports Sci Med Rehabil. 2026 Jul 8;18:456. doi: 10.1186/s13102-026-01864-w (PMC13632249; doi:10.1186/s13102-026-01864-w)
Supplement: Supplementary file 1 [file 13102_2026_1864_MOESM1_ESM.pdf]

Supplementary Appendix

Effects of Different Exercise-Based Rehabilitation Programs in Adults with Pulmonary Arterial Hypertension and Related Pulmonary Hypertension Populations: A Systematic Review and Network Meta-analysis

Chen Hua[1*], BD; Wenrui Huang[2], PhD; Zhen Chen[3], MD; Xiaoyang Cai[1]; Lin Tang[1]; Juan Jia[1]; Juan Yang[1], BD; Chen Zhang[1#], PhD.

Author Affiliations:

1. The Second Affiliated Hospital Of Army Medical University: Xinqiao Hospital, Chongqing, China
2. The Fourth Clinical Medical College of Guangzhou University of Chinese Medicine, Shenzhen, Guangdong, China
3. Army Medical University Army Health Service Training Base,Chongqing, China

* **Chen Hua** (first author)

\# **Chen Zhang (**corresponding author)

Correspondence

Chen Zhang, PhD

The Second Affiliated Hospital of Army Medical University, Xinqiao Hospital, Chongqing 400037, China

E-mail: elevenzc@tmmu.edu.cn

Tel: +86-17784255269

Conflicts of interest statement: The authors have no financial or proprietary interests in any material discussed in this article.

Primary Funding Source: None.

PROSPERO: CRD420251107670, Review Completed not published.

Word Count: Abstract: 438 words. Main text: 7,708 words.

Number of tables and figures: 36 tables, 38 figures

Keywords: Pulmonary arterial hypertension; aerobic exercise; breathing training; resistance exercise; network meta-analysis; rehabilitation

Appendix 1: Search strategy

Table S1. Search strategy of PubMed

#	Searches
1	(((Hypertension, Pulmonary[MeSH Terms]) OR (Pulmonary Arterial Hypertension[Title/Abstract])) OR (PAH[Title/Abstract])) OR (Pulmonary Hypertension[Title/Abstract])
2	((((((((((((((((((((((((Exercise[MeSH Terms]) OR (Physical Activity[Title/Abstract])) OR (Activities, Physical[Title/Abstract])) OR (Activity, Physical[Title/Abstract])) OR (Physical Activities[Title/Abstract])) OR (Exercise, Physical[Title/Abstract])) OR (Exercises, Physical[Title/Abstract])) OR (Physical Exercise[Title/Abstract])) OR (Physical Exercises[Title/Abstract])) OR (Acute Exercise[Title/Abstract])) OR (Acute Exercises[Title/Abstract])) OR (Exercise, Acute[Title/Abstract])) OR (Exercises, Acute[Title/Abstract])) OR (Exercise, Isometric[Title/Abstract])) OR (Exercises, Isometric[Title/Abstract])) OR (Isometric Exercises[Title/Abstract])) OR (Isometric Exercise[Title/Abstract])) OR (Exercise, Aerobic[Title/Abstract])) OR (Aerobic Exercise[Title/Abstract])) OR (Aerobic Exercises[Title/Abstract])) OR (Exercises, Aerobic[Title/Abstract])) OR (Exercise Training[Title/Abstract])) OR (Exercise Trainings[Title/Abstract])) OR (Training, Exercise[Title/Abstract])) OR (Trainings, Exercise[Title/Abstract])
3	(((((((((((((((((((((((((((((((((Muscle Stretching Exercises[MeSH Terms]) OR (Exercise, Muscle Stretching[Title/Abstract])) OR (Muscle Stretching Exercise[Title/Abstract])) OR (Static Stretching[Title/Abstract])) OR (Stretching, Static[Title/Abstract])) OR (Active Stretching[Title/Abstract])) OR (Stretching, Active[Title/Abstract])) OR (Static-Active Stretching[Title/Abstract])) OR (Static Active Stretching[Title/Abstract])) OR (Stretching, Static-Active[Title/Abstract])) OR (Isometric Stretching[Title/Abstract])) OR (Stretching, Isometric[Title/Abstract])) OR (Ballistic Stretching[Title/Abstract])) OR (Stretching, Ballistic[Title/Abstract])) OR (Dynamic Stretching[Title/Abstract])) OR (Stretching, Dynamic[Title/Abstract])) OR (Proprioceptive Neuromuscular Facilitation (PNF) Stretching[Title/Abstract])) OR (PNF Stretching[Title/Abstract])) OR (PNF Stretchings[Title/Abstract])) OR (Stretching, PNF[Title/Abstract])) OR (PNF Stretching Exercise[Title/Abstract])) OR (Exercise, PNF Stretching[Title/Abstract])) OR (PNF Stretching Exercises[Title/Abstract])) OR (Stretching Exercise, PNF[Title/Abstract])) OR (Proprioceptive Neuromuscular Facilitation[Title/Abstract])) OR (Neuromuscular Facilitation, Proprioceptive[Title/Abstract])) OR (Proprioceptive Neuromuscular Facilitations[Title/Abstract])) OR (Passive Stretching[Title/Abstract])) OR (Stretching, Passive[Title/Abstract])) OR (Relaxed Stretching[Title/Abstract])) OR (Stretching, Relaxed[Title/Abstract])) OR (Static-Passive Stretching[Title/Abstract])) OR (Static Passive Stretching[Title/Abstract])) OR (Stretching, Static-Passive[Title/Abstract])
4	(((((Physical Conditioning, Human[MeSH Terms]) OR (Conditioning, Human Physical[Title/Abstract])) OR (Human Physical Conditioning[Title/Abstract])) OR (Physical Training, Human[Title/Abstract])) OR (Human Physical Training[Title/Abstract])) OR (Training, Human Physical[Title/Abstract])
5	((((((Circuit-Based Exercise[MeSH Terms]) OR (Circuit Based Exercise[Title/Abstract])) OR (Circuit-Based Exercises[Title/Abstract])) OR (Exercise, Circuit-Based[Title/Abstract])) OR (Exercises, Circuit-Based[Title/Abstract])) OR (Circuit Training[Title/Abstract])) OR (Training, Circuit[Title/Abstract])
6	(Endurance Training[MeSH Terms]) OR (Training, Endurance[Title/Abstract])
7	((((((((((((((((((((((((((((Plyometric Exercise[MeSH Terms]) OR (Exercise, Plyometric[Title/Abstract])) OR (Exercises, Plyometric[Title/Abstract])) OR (Plyometric Exercises[Title/Abstract])) OR (Plyometric Drill[Title/Abstract])) OR (Drill, Plyometric[Title/Abstract])) OR (Drills, Plyometric[Title/Abstract])) OR (Plyometric Drills[Title/Abstract])) OR (Plyometric Training[Title/Abstract])) OR (Plyometric Trainings[Title/Abstract])) OR (Training, Plyometric[Title/Abstract])) OR (Trainings,

	Plyometric[Title/Abstract])) OR (Stretch-Shortening Exercise[Title/Abstract])) OR (Exercise, Stretch-Shortening[Title/Abstract])) OR (Exercises, Stretch-Shortening[Title/Abstract])) OR (Stretch Shortening Exercise[Title/Abstract])) OR (Stretch-Shortening Exercises[Title/Abstract])) OR (Stretch-Shortening Cycle Exercise[Title/Abstract])) OR (Cycle Exercise, Stretch-Shortening[Title/Abstract])) OR (Cycle Exercises, Stretch-Shortening[Title/Abstract])) OR (Exercise, Stretch-Shortening Cycle[Title/Abstract])) OR (Exercises, Stretch-Shortening Cycle[Title/Abstract])) OR (Stretch Shortening Cycle Exercise[Title/Abstract])) OR (Stretch-Shortening Cycle Exercises[Title/Abstract])) OR (Stretch-Shortening Drill[Title/Abstract])) OR (Drill, Stretch-Shortening[Title/Abstract])) OR (Drills, Stretch-Shortening[Title/Abstract])) OR (Stretch Shortening Drill[Title/Abstract])) OR (Stretch-Shortening Drills[Title/Abstract])
8	(((((((((((High-Intensity Interval Training[MeSH Terms]) OR (High Intensity Interval Training[Title/Abstract])) OR (High-Intensity Interval Trainings[Title/Abstract])) OR (Interval Training, High-Intensity[Title/Abstract])) OR (Interval Trainings, High-Intensity[Title/Abstract])) OR (Training, High-Intensity Interval[Title/Abstract])) OR (Trainings, High-Intensity Interval[Title/Abstract])) OR (High-Intensity Intermittent Exercise[Title/Abstract])) OR (Exercise, High-Intensity Intermittent[Title/Abstract])) OR (Exercises, High-Intensity Intermittent[Title/Abstract])) OR (High-Intensity Intermittent Exercises[Title/Abstract])) OR (Sprint Interval Training[Title/Abstract])) OR (Sprint Interval Trainings[Title/Abstract])
9	((((((((((((((((((((((Resistance Training[MeSH Terms]) OR (Training, Resistance[Title/Abstract])) OR (Strength Training[Title/Abstract])) OR (Training,Strength[Title/Abstract])) OR (Weight-Lifting Strengthening Program[Title/Abstract])) OR (Strengthening Program, Weight-Lifting[Title/Abstract])) OR (Strengthening Programs, Weight-Lifting[Title/Abstract])) OR (Weight Lifting Strengthening Program[Title/Abstract])) OR (Weight-Lifting Strengthening Programs[Title/Abstract])) OR (Weight-Lifting Exercise Program[Title/Abstract])) OR (Exercise Program, Weight-Lifting[Title/Abstract])) OR (Exercise Programs, Weight-Lifting[Title/Abstract])) OR (Weight Lifting Exercise Program[Title/Abstract])) OR (Weight-Lifting Exercise Programs[Title/Abstract])) OR (Weight-Bearing Strengthening Program[Title/Abstract])) OR (Strengthening Program, Weight-Bearing[Title/Abstract])) OR (Strengthening Programs, Weight-Bearing[Title/Abstract])) OR (Weight Bearing Strengthening Program[Title/Abstract])) OR (Weight-Bearing Strengthening Programs[Title/Abstract])) OR (Weight-Bearing Exercise Program[Title/Abstract])) OR (Exercise Program, Weight-Bearing[Title/Abstract])) OR (Exercise Programs, Weight-Bearing[Title/Abstract])) OR (Weight Bearing Exercise Program[Title/Abstract])) OR (Weight-Bearing Exercise Programs[Title/Abstract])

10	(Running[MeSH Terms]) OR (Runnings[Title/Abstract])
11	(Jogging[MeSH Terms]) OR (joggings[Title/Abstract])
12	(((((Marathon Running[MeSH Terms]) OR (Running, Marathon[Title/Abstract])) OR (Marathons[Title/Abstract])) OR (Marathon[Title/Abstract])) OR (Ultramarathon Running[Title/Abstract])) OR (Running, Ultramarathon[Title/Abstract])
13	Swimming[MeSH Terms]
14	(Walking[MeSH Terms]) OR (Ambulation[[Title/Abstract])
15	(((Nordic Walking[MeSH Terms]) OR (Walking, Nordic[Title/Abstract])) OR (Pole Walking[Title/Abstract])) OR (Walking, Pole[Title/Abstract])
16	((((((((((Exercise Therapy[MeSH Terms]) OR (Remedial Exercise[MeSH Terms])) OR (Exercise, Remedial[MeSH Terms])) OR (Exercises, Remedial[MeSH Terms])) OR (Remedial Exercises[MeSH Terms])) OR (Therapy, Exercise[MeSH Terms])) OR (Exercise Therapies[MeSH Terms])) OR (Therapies, Exercise[MeSH Terms])) OR (Rehabilitation Exercise[MeSH Terms])) OR (Exercise, Rehabilitation[MeSH Terms])) OR (Exercises, Rehabilitation[MeSH Terms])) OR (Rehabilitation Exercises[MeSH Terms])
17	(((Sports[MeSH Terms]) OR (Sport[Title/Abstract])) OR (Athletics[Title/Abstract])) OR (Athletic[Title/Abstract])
18	(((((((((((Martial Arts[MeSH Terms]) OR (Arts, Martial[Title/Abstract])) OR (Hap Ki Do[Title/Abstract])) OR (Judo[Title/Abstract])) OR (Karate[Title/Abstract])) OR (Jujitsu[Title/Abstract])) OR (Tae Kwon Do[Title/Abstract])) OR (Aikido[Title/Abstract])) OR (Wushu[Title/Abstract])) OR (Kung Fu[Title/Abstract])) OR (Gong Fu[Title/Abstract])) OR (Fu, Gong[Title/Abstract])) OR (Gongfu[Title/Abstract])
19	((((((((((Tai Ji[MeSH Terms]) OR (Tai-ji[Title/Abstract])) OR (Tai Chi[Title/Abstract])) OR (Chi, Tai[Title/Abstract])) OR (Tai Ji Quan[Title/Abstract])) OR (Ji Quan, Tai[Title/Abstract])) OR (Quan, Tai Ji[Title/Abstract])) OR (Taiji[Title/Abstract])) OR (Taijiquan[Title/Abstract])) OR (T'ai Chi[Title/Abstract])) OR (Tai Chi Chuan[Title/Abstract])
20	(((((((Mind-Body Therapies[MeSH Terms]) OR (Mind Body Therapies[Title/Abstract])) OR (Mind-Body Therapy[Title/Abstract])) OR (Therapies, Mind-Body[Title/Abstract])) OR (Therapy, Mind-Body[Title/Abstract])) OR (Mind-Body Medicine[Title/Abstract])) OR (Mind Body Medicine[Title/Abstract])) OR (mind-body exercise*[Title/Abstract])
21	((((Breathing Exercises[MeSH Terms]) OR (Exercise, Breathing[Title/Abstract])) OR (Respiratory Muscle Training[Title/Abstract])) OR (Muscle Training, Respiratory[Title/Abstract])) OR (Training, Respiratory Muscle[Title/Abstract])

22	((Qigong[MeSH Terms]) OR (Qi Gong[Title/Abstract])) OR (Ch'i Kung[Title/Abstract])
23	Yoga[MeSH Terms]
24	((Bicycle[Title/Abstract]) OR (cycling[Title/Abstract])) OR (aquagym[Title/Abstract])) OR (brisk walking[Title/Abstract])) OR (setting-up exercise[Title/Abstract])) OR (square dance[Title/Abstract])) OR (indoor cycle ergometer[Title/Abstract])) OR (treadmill exercise[Title/Abstract])) OR (brisk walking[Title/Abstract])) OR (equipment training[Title/Abstract])) OR (dumbbell exercise[Title/Abstract])) OR (resistance band[Title/Abstract])) OR (elastic resistance band[Title/Abstract])) OR (elastic band[Title/Abstract])) OR (Thera-band[Title/Abstract])) OR (bodyweight training[Title/Abstract])) OR (combined strength training[Title/Abstract])) OR (total resistance exercise[Title/Abstract])) OR (Aggravate exercise[Title/Abstract])) OR (Pilates[Title/Abstract])) OR (Traditional Chinese exercises[Title/Abstract])) OR (TCE[Title/Abstract])) OR (TCEs[Title/Abstract])) OR (transitional exercises[Title/Abstract])) OR (Traditional Chinese exercise[Title/Abstract])) OR (traditional Chinese health exercise[Title/Abstract])) OR (traditional Chinese medical skills[Title/Abstract])) OR (Chinese traditional sports[Title/Abstract])) OR (traditional Kungfu[Title/Abstract])) OR (Kungfu[Title/Abstract])) OR (Gong[Title/Abstract])) OR (Shadowboxing[Title/Abstract])) OR (Taijiquan[Title/Abstract])) OR (Five-animal exercises[Title/Abstract])) OR (Wuqinxi[Title/Abstract])) OR (Five mimic-animal boxing[Title/Abstract])) OR (Five-animal boxing[Title/Abstract])) OR (Baduanjin[Title/Abstract])) OR (Baduanjin exercise[Title/Abstract])) OR (Yijinjing[Title/Abstract])) OR (Shaolin Neigong[Title/Abstract])) OR (Yijinjing[Title/Abstract])) OR (tendon exercise[Title/Abstract])) OR (Liuzijue[Title/Abstract])
25	((((((((((((((((((((continuous training[Title/Abstract]) OR (continuous exercise[Title/Abstract])) OR (continuous-type exercise[Title/Abstract])) OR (continuous-type training[Title/Abstract])) OR (continuous training[Title/Abstract])) OR (moderate intensity continuous training[Title/Abstract])) OR (continuous medium intensity training[Title/Abstract])) OR (continuous moderate intensity exercise[Title/Abstract])) OR (continuous moderate intensity training[Title/Abstract])) OR (medium intensity continuous training[Title/Abstract])) OR (MICT (training)[tiab] OR moderate intensity continuous exercise)) OR (moderate intensity continuous training[Title/Abstract])) OR (moderate intensity exercise[Title/Abstract])) OR (medium intensity exercise[Title/Abstract])) OR (medium intensity physical activity[Title/Abstract])) OR (medium intensity training[Title/Abstract])) OR (medium intensity work-out[Title/Abstract])) OR (medium intensity workout[Title/Abstract])) OR (moderate intensity physical activity[Title/Abstract])) OR (moderate intensity training[Title/Abstract])) OR (moderate intensity work-out'[Title/Abstract])) OR (moderate intensity workout[Title/Abstract])) OR (moderate intensity exercise[Title/Abstract])

26	OR/#2-#25
27	((((Randomized Controlled Trials as Topic[MeSH Terms]) OR (Clinical Trials, Randomized[Title/Abstract])) OR (Trials, Randomized Clinical[Title/Abstract])) OR (Controlled Clinical Trials, Randomized[Title/Abstract])) OR (RCT[Title/Abstract])
28	#1 AND #26 AND #27

Table S2. Search strategy of Web of Science

#	Searches
1	TS = ("Pulmonary Arterial Hypertension" OR "Pulmonary Hypertension" OR "PAH")
2	MH=Exercises OR TS=(Physical Activity OR Activities, Physical OR Activity, Physical OR Physical Activities OR Exercise, Physical OR Exercises, Physical OR Physical Exercise OR Physical Exercises OR Acute Exercise OR Acute Exercises OR Exercise, Acute OR Exercises, Acute OR Exercise, Isometric OR Exercises, Isometric OR Isometric Exercises OR Isometric Exercise OR Exercise, Aerobic OR Aerobic Exercise OR Aerobic Exercises OR Exercises, Aerobic OR Exercise Training OR Exercise Trainings OR Training, Exercise OR Trainings, Exercise OR Endurance Training OR Training, Endurance)
3	MH=Muscle Stretching Exercises OR TS=(Exercise, Muscle Stretching OR Muscle Stretching Exercise OR Static Stretching OR Stretching, Static OR Active Stretching OR Stretching, Active OR Static-Active Stretching OR Static Active Stretching OR Stretching, Static-Active OR Isometric Stretching OR Stretching, Isometric OR Ballistic Stretching OR Stretching, Ballistic OR Dynamic Stretching OR Stretching, Dynamic OR Proprioceptive Neuromuscular Facilitation (PNF) Stretching OR PNF Stretching OR PNF Stretchings OR Stretching, PNF OR PNF Stretching Exercise OR Exercise, PNF Stretching OR PNF Stretching Exercises OR Stretching Exercise, PNF OR Proprioceptive Neuromuscular Facilitation OR Neuromuscular Facilitation, Proprioceptive OR Proprioceptive Neuromuscular Facilitations OR Passive Stretching OR Stretching, Passive OR Relaxed Stretching OR Stretching, Relaxed OR Static-Passive Stretching OR Static Passive Stretching OR Stretching, Static Passive)
4	OR/#2-#3
5	TS= clinical trial* OR TS=research design OR TS=comparative stud* OR TS=evaluation stud* OR TS=controlled trial* OR TS=follow-up stud* OR TS=prospective stud* OR TS=random* OR TS=placebo* OR TS=(single blind*) OR TS=(double blind*)
6	#1 AND #4 AND #5

Table S3. Search strategy of Cochrane Library

#	Searches
1	MeSH descriptor: [Hypertension, Pulmonary] explode all trees
2	("Pulmonary Arterial Hypertension" OR "Pulmonary Hypertension" OR "PAH"):ti,ab,kw

3	#1 OR #2
4	MeSH descriptor: [Exercise] explode all trees
5	(Isometric Exercise; Exercise, Isometric; Exercises, Isometric; Isometric Exercises; Exercise Trainings; Training, Exercise; Exercise Training; Trainings, Exercise; Physical Exercises; Exercises, Physical; Physical Activity; Activity, Physical; Exercise, Physical; Exercises; Activities, Physical; Physical Exercise; Physical Activities; Exercise, Aerobic; Aerobic Exercise; Exercises, Aerobic; Aerobic Exercises; Acute Exercises; Exercise, Acute; Exercises, Acute; Acute Exercise):ti,ab,kw
6	OR/#4-#5
7	(#3 AND #6) AND (Trials)filter

Table S4. Search strategy of Embase

#	Searches
1	'pulmonary hypertension'/exp OR 'pulmonary arterial hypertension':ti,ab,kw OR 'PAH':ti,ab,kw
2	‘exercise’/exp OR ('biometric exercise' OR 'effort' OR 'exercise capacity' OR 'exercise performance' OR 'exercise training' OR 'exertion' OR 'fitness training' OR 'fitness workout' OR 'physical conditioning, human' OR 'physical effort' OR 'physical exercise' OR 'physical exertion' OR 'physical work-out' OR 'physical workout' OR 'exercise'):ti,ab,kw
3	('randomized controlled trial'/exp OR 'clinical trial, randomized',ab OR 'trials, randomized clinical',ab OR 'controlled clinical trial, randomized',ab OR 'rct',ab)
4	#1 AND #2 AND #3

Table S5. Search strategy of CNKI

#	Searches
1	主题 = “肺动脉高压” OR “肺高血压” OR “肺性高血压” OR “pulmonary arterial hypertension” OR “pulmonary hypertension” OR “PAH”
2	主题 =“运动” OR “运动训练” OR “运动疗法” OR “运动康复” OR “康复训练” OR “有氧运动” OR “抗阻训练” OR “阻力训练” OR “力量训练” OR “呼吸训练” OR “呼吸肌训练” OR “吸气肌训练” OR “呼吸功能训练” OR “五禽戏” OR “太极” OR “八段锦” OR “气功”
3	主题 = “随机” OR “随机对照试验” OR “随机临床试验” OR “临床试验” OR “对照试验” OR “RCT”
4	#1 AND #2 AND #3

Table S6. Search strategy of VIP

#	Searches
1	题名/关键词/摘要 ＝ “肺动脉高压” OR “肺高血压” OR “肺性高血压” OR “pulmonary arterial hypertension” OR “pulmonary hypertension” OR “PAH”
2	题名/关键词/摘要 ＝“运动” OR “运动训练” OR “运动疗法” OR “运动康复” OR “康复训练” OR “有氧运动” OR “抗阻训练” OR “阻力训练” OR “力量训练” OR “呼吸训练” OR “呼吸肌训练” OR “吸气肌训练” OR “呼吸功能训练” OR “五禽戏” OR “太极” OR “八段锦” OR “气功”
3	题名/关键词/摘要 ＝ “随机” OR “随机对照试验” OR “随机临床试验” OR “临床试验” OR “对照试验” OR “RCT”
4	#1 AND #2 AND #3

Table S7. Search strategy of Wanfang

#	Searches
1	主题 ＝ “肺动脉高压” OR “肺高血压” OR “肺性高血压” OR “pulmonary arterial hypertension” OR “pulmonary hypertension” OR “PAH”
2	主题 ＝“运动” OR “运动训练” OR “运动疗法” OR “运动康复” OR “康复训练” OR “有氧运动” OR “抗阻训练” OR “阻力训练” OR “力量训练” OR “呼吸训练” OR “呼吸肌训练” OR “吸气肌训练” OR “呼吸功能训练” OR “五禽戏” OR “太极” OR “八段锦” OR “气功”
3	主题 ＝ “随机” OR “随机对照试验” OR “随机临床试验” OR “临床试验” OR “对照试验” OR “RCT”
4	#1 AND #2 AND #3

Table S8. Search strategy of trial registries

Registry	Search terms	Search date	Purpose
ClinicalTrials.gov	“pulmonary arterial hypertension” OR “pulmonary hypertension” OR PAH; exercise OR rehabilitation OR training OR “breathing training” OR “inspiratory muscle training”	2 June 2026	To identify ongoing, unpublished or terminated trials
WHO International Clinical Trials Registry Platform	“pulmonary arterial hypertension” OR “pulmonary hypertension” OR PAH; exercise OR rehabilitation OR training OR “breathing training” OR “inspiratory muscle training”	2 June 2026	To identify ongoing, unpublished or terminated trials
Chinese Clinical Trial Registry	肺动脉高压” OR “肺高血压”; “运动” OR “康复” OR “运动训练” OR “呼吸训练” OR “呼吸肌训练”	2 June 2026	To identify ongoing, unpublished or terminated trials

Appendix 2: Studies excluded after full-text review and primary reasons for exclusion

Table S2.1. Studies excluded after full-text review and primary reasons for exclusion

First author	Year	Title	Primary reason for exclusion
Savarese	2013	Haemodynamics, exercise capacity and clinical events in pulmonary arterial hypertension	Ineligible intervention
Morris	2023	Exercise-based rehabilitation programmes for pulmonary hypertension	Not an RCT
Ennis	2023	Development of a complex exercise rehabilitation intervention for people with pulmonary hypertension: the supervised pulmonary hypertension exercise rehabilitation (SPHERe) trial	Not an RCT
Mathai	2016	Quality of life in patients with chronic thromboembolic pulmonary hypertension	Not an RCT
Arena	2015	The Effect of Exercise Training on the Pulmonary Arterial System in Patients With Pulmonary Hypertension	Not an RCT
Ganderton	2011	Short term effects of exercise training on exercise capacity and quality of life in patients with pulmonary arterial hypertension	Not an RCT
Inagaki	2014	Home-based pulmonary rehabilitation in patients with inoperable or residual chronic thromboembolic pulmonary hypertension: A preliminary study	Not an RCT
Mainguy	2010	Effects of a Rehabilitation Program on Skeletal Muscle Function in Idiopathic Pulmonary Arterial Hypertension	Not an RCT
de Man	2009	Effects of exercise training in patients with idiopathic pulmonary arterial hypertension	Not an RCT
Grünig	2011	Effect of Exercise and Respiratory Training on Clinical Progression and Survival in Patients with Severe Chronic Pulmonary Hypertension	Not an RCT
Nagel	2012	Exercise Training Improves Exercise Capacity and Quality of Life in Patients with Inoperable or Residual Chronic Thromboembolic Pulmonary Hypertension	Not an RCT
Becker-Grünig	2012	Efficacy of exercise training in pulmonary arterial hypertension associated with congenital heart disease	Not an RCT
Kabitz	2014	The Combination of Exercise and Respiratory Training Improves Respiratory Muscle Function in Pulmonary Hypertension	Not an RCT
Nagel	2020	Supervised Exercise Training in Patients with Chronic Thromboembolic Pulmonary Hypertension as Early Follow-Up Treatment after Pulmonary Endarterectomy: A Prospective Cohort Study	Not an RCT

First author	Year	Title	Primary reason for exclusion
Spruijt	2015	The effects of exercise on right ventricular contractility and right ventricular-arterial coupling in pulmonary hypertension	Not an RCT
Cícero	2012	Lack of Tight Association Between Quality of Life and Exercise Capacity in Pulmonary Arterial Hypertension	Not an RCT
Ley	2013	Magnetic resonance imaging to assess the effect of exercise training on pulmonary perfusion and blood flow in patients with pulmonary hypertension	Not an RCT
Weinstein	2013	Effect of aerobic exercise training on fatigue and physical activity in patients with pulmonary arterial hypertension	Not an RCT
Woolstenhulme	2019	Left Ventricular Function Before and After Aerobic Exercise Training in Women With Pulmonary Arterial Hypertension	Not an RCT
Santos-Lozano	2019	Exercise Benefits in Pulmonary Hypertension	Not an RCT
Nagel	2021	Effect of Supervised Training Therapy on Pulmonary Arterial Compliance and Stroke Volume in Severe Pulmonary Arterial Hypertension and Inoperable or Persistent Chronic Thromboembolic Pulmonary Hypertension	Not an RCT
Ehlken	2014	Economic Evaluation of Exercise Training in Patients with Pulmonary Hypertension	Not an RCT
Glöckl	2019	Exercise Training in Patients with Pulmonary Hypertension: A Systematic Review and Meta-analysis	Not an RCT
Yan	2021	The benefit of exercise-based rehabilitation programmes in patients with pulmonary hypertension: a systematic review and meta-analysis of randomized controlled trials	Not an RCT
An	2021	Effectiveness and safety of exercise training and rehabilitation in chronic thromboembolic pulmonary hypertension: a systematic review and meta-analysis	Not an RCT
Sarzyńska	2021	Quality of life of patients with pulmonary arterial hypertension: a meta-analysis	Not an RCT
Dong	2022	Exercise Rehabilitation Training in Patients with Pulmonary Hypertension: A Review	Not an RCT
Satyamurthy	2023	Exercise Training in Pulmonary Hypertension: An Updated Systematic Review With Meta-analysis	Not an RCT
VanAken	2025	Exercise Training in Patients with Chronic Thromboembolic Pulmonary Hypertension and Pulmonary Arterial Hypertension: A Systematic Review and Meta-Analysis of Randomized Controlled Trials	Not an RCT

First author	Year	Title	Primary reason for exclusion
Jiang	2022	肺动脉高压患者采取呼吸功能锻炼干预的效果分析	Ineligible population
Ma	2024	呼吸肌训练对高海拔肺动脉高压患者的运动耐量与心功能影响的研究	Ineligible population
Wang	2025	呼吸康复对慢性阻塞性肺疾病相关肺高血压患者运动能力及生活质量的影响	Ineligible population

Appendix 3: Characteristics of included studies

Table S3.1: Baseline characteristics and pulmonary hypertension subtype of included studies

Study ID	Country	Study setting	Design	Sample size (T/C)	Age (Year, T/C)	Sex distribution, n (%)	PH subtype / population	Reason if excluded from PAH-only analysis	Included in PAH-only sensitivity analysis	Baseline functional status	Outcomes
Jia 2024[15]	China	Single center	Parallel-group RCT	30/30	-/-	-	PAH	Not applicable	Yes	WHO FC II-III	①②③
Jia 2019[16]	China	Single center	Parallel-group RCT	23/26	56.7 ± 24.4/55.3±25.2	T 11/12, C 12/14 (M/F)	PAH	Not applicable	Yes	WHO FC II-III	②⑤
Zhang 2025[17]	China	Single center	Parallel-group RCT	26/28	47.6±6.8/50.3± 10.6	T 7/19, C 5/23 (M/F)	PAH	Not applicable	Yes	WHO FC I-II	④⑦
Ehlken 2016[18]	Germany	Single center	Parallel-group RCT	38/41	55±15/57±15	T 20/26, C 20/21 (M/F)	PAH	Not applicable	Yes	/	②③④⑥⑦
Chan 2013[19]	USA	Single center	Parallel-group RCT	10/13	53 ±13/55.5± 8.5	T 0/10, C 0/13 (M/F)	PAH	Not applicable	Yes	WHO FC I-IV	②④
Mereles 2006[20]	Germany	Single center	Parallel-group RCT	15/15	47±12/53±14	T 5/10, C 5/10 (M/F)	PAH+CTEPH	Mixed PAH/CTEPH population	No	WHO FC II-IV	②④⑥⑦
González-Saiz 2017[21]	Spain	Single center	Parallel-group RCT	20/20	46±11/45±12	T 8/12, C 8/12 (M/F)	PAH+CTEPH	Mixed PAH/CTEPH population	No	WHO FC I-III	①②③
Yılmaz 2020[22]	Turkey	Single center	Parallel-group RCT	11/11	35±13.68/40.18±14.47	T 1/10, C 1/10 (M/F)	PAH	Not applicable	Yes	/	②③④⑥⑦

Hemnes 2021[23]	USA	Multicenter	Parallel-group RCT	20/22	47±16/47±10	T 5/15, C 1/21 (M/F)	PAH	Not applicable	Yes	WHO FC I-III	①②⑥
Martinez-Quintana 2010[24]	Spain	Single center	Parallel-group RCT	4/4	22.7±6.9/32.7±5.5	T 2/2, C 3/1 (M/F)	PAH	CHD-associated PAH/PH population	No	WHO FC II-IV	②⑥⑦
Saglam 2015[25]	Turkey	Single center	Parallel-group RCT	14/15	46.8±15.6/52.2±8.8	T 3/11, C 3/14 (M/F)	PAH	Not applicable	Yes	WHO FC II-III	②⑤⑥
Kagioglou 2021[26]	Greece	Single center	Parallel-group RCT	12/10	54.7±15.6/53.1±12.1	Overall 9/13 (M/F); group-specific NR	PAH+CTEPH	Mixed PAH/CTEPH population	No	WHO FC I-III	①②④⑦
Rakhmawati 2020[27]	Indonesia	Multicenter	Parallel-group RCT	20/18	37.5±8.8/35.5±10.4	T 1/19, C 2/16 (M/F)	PAH	CHD-associated PAH/PH population	No	WHO FC I-II	③
Karapolat 2019[28]	Turkey	Single center	Parallel-group RCT	12/12	44±9 /47±13.5	T 3/9, C 8/4 (M/F)	PAH	Not applicable	Yes	WHO FC II-III	①②④⑤
Tran 2021[29]	Australia	Multicenter	Parallel-group RCT	5/5	55±17/66±10	T 1/4, C 1/4 (M/F)	CTEPH	CTEPH-only population	No	WHO FC II-III	④⑤⑦
Babu 2019[30]	Indian	Single center	Parallel-group RCT	42/42	51.45±13.7/47.28±15.6	T 22/20, C 29/13 (M/F)	PAH	Not applicable	Yes	WHO FC II-IV	①②

NOTE: ①SF-36；②6MWD；③NT-proBNP；④Peak VO_2；⑤FEV_1%；⑥HR rest；⑦HR max. PAH, pulmonary arterial hypertension; PH, pulmonary hypertension; CTEPH, chronic thromboembolic pulmonary hypertension; CHD, congenital heart disease; RCT, randomised controlled trial; T, intervention group; C, control group; WHO FC, World Health Organization functional class; NMA, network meta-analysis; SF-36, Short Form-36; 6MWD, 6-minute walk distance; NT-proBNP, N-terminal pro-B-type natriuretic peptide; VO2, oxygen uptake; FEV1%, forced expiratory volume in 1 second percentage predicted; HR, heart rate; M/F, male/female; NR, not reported. PAH-only sensitivity analyses excluded mixed PAH/CTEPH, CTEPH-only and congenital heart disease-associated PAH/PH studies.

Appendix 3: Characteristics of included studies

Table S3.2: Intervention dose, delivery characteristics and node classification of included studies

Study ID	Intervention node	Comparator	Setting	Supervision	Frequency	Session duration	Programme duration	Intensity	Main components
Jia 2024[15]	AE	UC	-	Supervised/structured practice, as reported	7/week	~60 min	3 months	Low	Wuqinxi (bird-play) exercise
Jia 2019[16]	AE+BT	UC	Hospital instruction then home-based	Partially supervised/home-based after teaching	7/week	30 min	3 months	Low	Breathing exercises + whole-body breathing exercises
Zhang 2025[17]	AE	UC	Hospital	Supervised	3/week	45 min	3 months	50–80% heart rate reserve	Cycle ergometer training

Ehlken 2016[18]	AE+BT	UC	3-week rehabilitation clinic + 12-week home programme	Supervised then home-based	3/week (plus structured home training)	~60 min	3.75 months	Low	Exercise training + respiratory training
Chan 2013[19]	AE	UC	Outpatient	Supervised	3/week	30–45 min	2.5 months	70–80% heart rate reserve	Treadmill walking
Mereles 2006[20]	AE+BT	UC	3-week in-hospital training + home continuation	Supervised then home-based	5/week	15–30 min	3.75 months	High	Exercise training + respiratory training
González-Saiz 2017[21]	AE+BT	UC	Hospital-based	Supervised	5/week	20–40 min	2 months	-	Aerobic exercise + inspiratory muscle training
Yılmaz 2020[22]	AE	UC	Hospital-based	Supervised	7/week	20 min	1.5 months	High (50 − 80%)	Upper-extremity aerobic exercise
Hemnes 2021[23]	AE	UC	Home-based/mobile health	Remote/technology-supported	7/week	30 min	3 months	Low	Walking-based physical activity intervention
Martinez-Quintana 2010[24]	AE+RE	UC	Home-based with supervision	Supervised	2/week	~30 min	3 months	-	Interval aerobic training + resistance exercises
Saglam 2015[25]	BT	UC	Hospital-based	Supervised	7/week	30 min	1.5 months	30% maximal inspiratory pressure	Inspiratory muscle training
Kagioglou 2021[26]	AE+RE	UC	Hospital-based	Supervised	3/week	30–40 min	1.5 months	Moderate (60–80% peak VO_2 HR)	Aerobic + resistance exercise, stretching and breathing/chest expansion exercises
Rakhmawati 2020[27]	AE	UC	Hospital + home-based	Partially supervised	3/week	30 min	3 months	Moderate (60–70%)	Walking/cycle-based exercise programme
Karapolat 2019[28]	AE+BT	AE	Hospital-based vs home-based	Supervised vs home programme	3/week	45–60 min	3 months	50–70% peak VO_2	Flexibility + aerobic exercise + breathing exercises
Tran 2021[29]	BT	UC	Hospital/home as reported	Supervised	5/week	~20 min	2 months	30–40%	Inspiratory muscle training
Babu 2019[30]	AE+BT	UC	Hospital/home-based	Supervised/home-based programme	3/week	30 min	3 months	Low	Home-based exercise training with aerobic and

					breathing components

Note: Intervention nodes were assigned according to the dominant planned exercise components. However, exercise frequency, session duration, programme duration, intensity, setting, supervision, progression, adherence support and co-interventions varied across trials. Therefore, node-level comparisons should be interpreted as comparisons of broad rehabilitation strategies rather than as isolated effects of exercise modality alone.

Appendix 3: Characteristics of included studies

Table S3.3: Study-level overview of RoB 2 judgements for included trials

Study ID	Randomization process	Deviations from intended interventions	Missing outcome data	Measurement of the outcome	Selection of reported result	Overall
Jia 2024[15]	Low risk	Low risk	Low risk	Low risk	Low risk	Low risk
Jia 2019[16]	Low risk	Low risk	Low risk	Low risk	Low risk	Low risk
Zhang 2025[17]	Low risk	Low risk	Low risk	High risk	Some concerns	High risk
Ehlken 2016[18]	Low risk	Low risk	Low risk	High risk	Some concerns	High risk
Chan 2013[19]	High risk	Low risk	Low risk	High risk	Low risk	High risk
Mereles 2006[20]	Low risk	Low risk	Low risk	Low risk	Some concerns	Low risk
González-Saiz 2017[21]	Some concerns	Low risk	Low risk	Low risk	Some concerns	Low risk
Yılmaz 2020[22]	Low risk	Low risk	Low risk	Low risk	Low risk	Low risk
Hemnes 2021[23]	Low risk	Low risk	Low risk	Low risk	Low risk	Low risk
Martinez-Quintana 2010[24]	High risk	Low risk	Low risk	Low risk	Some concerns	High risk
Saglam 2015[25]	Low risk	Low risk	Low risk	Low risk	Some concerns	Some concerns

Kagioglou 2021[26]	Low risk	Low risk	Low risk	Low risk	Some concerns	Some concerns
Rakhmawati 2020[27]	Low risk	Low risk	Some concerns	Low risk	Some concerns	Some concerns
Karapolat 2019[28]	Low risk	Low risk	Some concerns	Some concerns	Some concerns	Some concerns
Tran 2021[29]	Low risk	Some concerns	Low risk	Some concerns	Some concerns	Some concerns
Babu 2019[30]	Low risk	Some concerns	Low risk	Low risk	Some concerns	Some concerns

Note: This table provides a descriptive study-level overview only. The primary RoB 2 assessment was performed at the outcome level. Outcome-level judgements used for certainty assessment and interpretation are reported in Supplementary Appendix 4, Table S4.1.

Table S3.4: Loop inconsistency test

**O**	**Loop**	**IF**	**P value**	**95%CI down,up**
SF-36	A-B-D	0.279	> 0.05	(0.00,1.68)
Peak VO_2	A-C-E	0.103	> 0.05	(0.00,1.81)

Appendix 4: Risk of bias of randomized clinical trials

Table S4.1. Outcome-level RoB 2 judgements by study and outcome

This table reports the overall RoB 2 judgement for each study-outcome combination contributing data to the network meta-analysis. Cells marked "No data" indicate that the study did not contribute data for that outcome and therefore no outcome-level RoB 2 judgement was required for that outcome.

Study ID	SF-36	6MWD	NT-proBNP	Peak VO_2	FEV_1%	Resting HR	Maximum HR	Main basis for judgement
Jia 2024[15]	Low	Low	Low	-	-	-	-	Low risk across reported RoB 2 domains.
Jia 2019[16]	-	Low	-	-	Low	-	-	Low risk across reported RoB 2 domains.
Zhang 2025[17]	-	-	-	High	-	-	High	High risk in outcome measurement; some concerns in selection of the reported result.
Ehlken 2016[18]	-	High	High	High	-	High risk	High	High risk in outcome measurement; some concerns in selection of the reported result.
Chan 2013[19]	-	High	-	High	-	-	-	High risk in randomisation process and outcome measurement.
Mereles 2006[20]	Low	Low	Low	-	-	Low	-	Low overall judgement in the current RoB 2 summary.
González-Saiz 2017[21]	-	Low	Low	Low	-	Low	Low	Low overall judgement in the current RoB 2 summary.
Yılmaz 2020[22]	Low	Low	-	-	-	Low	Low	Low risk across reported RoB 2 domains.
Hemnes 2021[23]	-	Low	-	-	-	Low	Low	Low risk across reported RoB 2 domains.
Martinez-Quintana 2010[24]	-	High	-	-	High	High	-	High risk in randomisation process; some concerns in selection of the reported result.
Saglam 2015[25]	Some concerns	Some concerns	-	Some concerns	-	-	Some concerns	Some concerns in selection of the reported result.
Kagioglou 2021[26]	-	Some concerns	-	Some concerns	-	Some concerns	-	Some concerns in selection of the reported result.
Rakhmawati 2020[27]	-	-	Some concerns	-	-	-	Some concerns	Some concerns in missing outcome data and selection of the reported result.
Karapolat 2019[28]	Some concerns	Some concerns	-	Some concerns	Some concerns	-	-	Some concerns in missing outcome data, outcome measurement and selection of the reported result.

Tran 2021[29]	-	-	-	Some concerns	Some concerns	-	Some concerns	Some concerns in deviations from intended interventions, outcome measurement and selection of the reported result.
Babu 2019[30]	Some concerns	Some concerns	-	Some concerns	-	-	-	Some concerns in deviations from intended interventions and selection of the reported result.

Note: RoB 2, Cochrane Risk of Bias 2 tool; SF-36, Short Form-36; 6MWD, 6-minute walk distance; NT-proBNP, N-terminal pro-B-type natriuretic peptide; peak VO_2, peak oxygen uptake; FEV_1%, forced expiratory volume in 1 second percentage predicted; HR, heart rate. The study-level overview in Table S3.3 is descriptive only. Outcome-level judgements in this table should be used for outcome-specific certainty assessment and interpretation. If a study contributed multiple outcomes and the RoB 2 judgement differed by outcome in the original RoB 2 files, the outcome-specific judgement should take precedence.

Appendix 4: Risk of bias of randomized clinical trials

Figure S4.2: Overall risk of bias presented as percentage of each risk-of-bias item across all included studies.

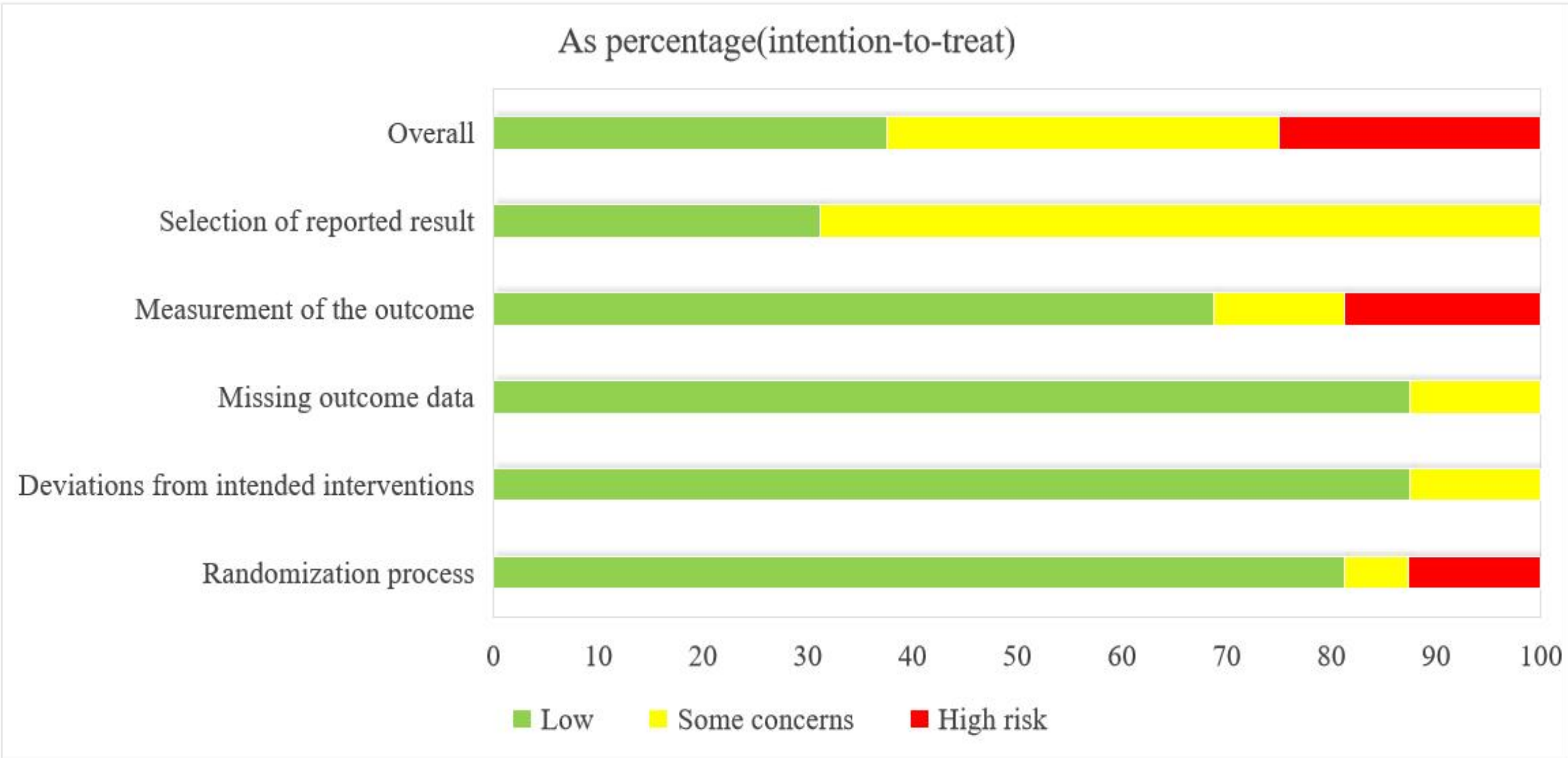

Appendix 5: Pairwise meta-analysis forest plots

Figure S5.1. Pairwise meta-analysis forest plot for overall/composite SF-36 score

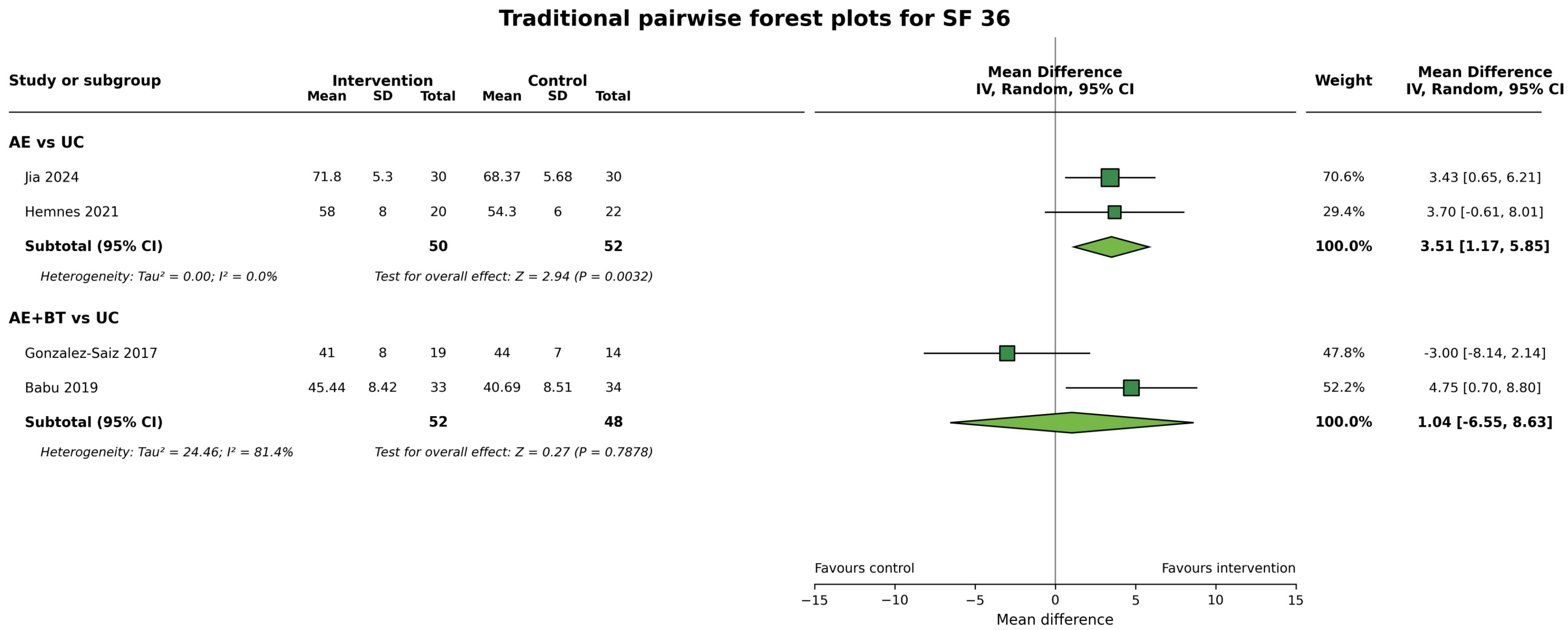

Figure S5.2. Pairwise meta-analysis forest plot for 6MWD

Traditional pairwise forest plots for 6MWD

Study or subgroup	Intervention Mean	Intervention SD	Intervention Total	Control Mean	Control SD	Control Total	Weight	Mean Difference IV, Random, 95% CI
**AE vs UC**								
Jia 2024	414.57	59.4	30	388.07	7.14	30	53.4%	26.50 [5.09, 47.91]
Chan 2013	467	86	10	389	107	13	3.9%	78.00 [-0.90, 156.90]
Yilmaz 2020	431.93	103.87	11	402.25	133.87	11	2.4%	29.68 [-70.45, 129.81]
Hemnes 2021	431	31.5	20	399	49	22	40.2%	32.00 [7.30, 56.70]
**Subtotal (95% CI)**			**71**			**76**	**100.0%**	**30.81 [15.16, 46.47]**
Heterogeneity: Tau² = 0.00; I² = 0.0%	*Test for overall effect: Z = 3.86 (P = 0.0001)*							
**BT vs UC**								
Ehlken 2016	482	105	38	401	106	41	63.3%	81.00 [34.45, 127.55]
Saglam 2015	476.43	90.11	14	334	121.6	15	36.7%	142.43 [64.87, 219.99]
**Subtotal (95% CI)**			**52**			**56**	**100.0%**	**103.56 [45.52, 161.61]**
Heterogeneity: Tau² = 821.85; I² = 43.6%	*Test for overall effect: Z = 3.50 (P = 0.0005)*							
**AE+BT vs UC**								
Jia 2019	425	33.5	23	380.58	50.56	26	31.6%	44.42 [20.65, 68.19]
Mereles 2006	85	56	15	12	37	15	26.9%	73.00 [39.03, 106.97]
Gonzalez-Saiz 2017	528	68	20	551	99	20	19.0%	-23.00 [-75.64, 29.64]
Babu 2019	334.1	88.4	34	275.3	93.9	33	22.5%	58.80 [15.10, 102.50]
**Subtotal (95% CI)**			**92**			**94**	**100.0%**	**42.52 [10.07, 74.96]**
Heterogeneity: Tau² = 719.76; I² = 68.0%	*Test for overall effect: Z = 2.57 (P = 0.0102)*							
**AE+RE vs UC**								
Martinez-Quintana 2010	308.2	93.1	4	376.5	209.5	4	26.7%	-68.30 [-292.97, 156.37]
Kagioglou 2021	525	112	12	435	64	10	73.3%	90.00 [15.24, 164.76]
**Subtotal (95% CI)**			**16**			**14**	**100.0%**	**47.76 [-89.48, 184.99]**
Heterogeneity: Tau² = 5232.25; I² = 41.8%	*Test for overall effect: Z = 0.68 (P = 0.4952)*							

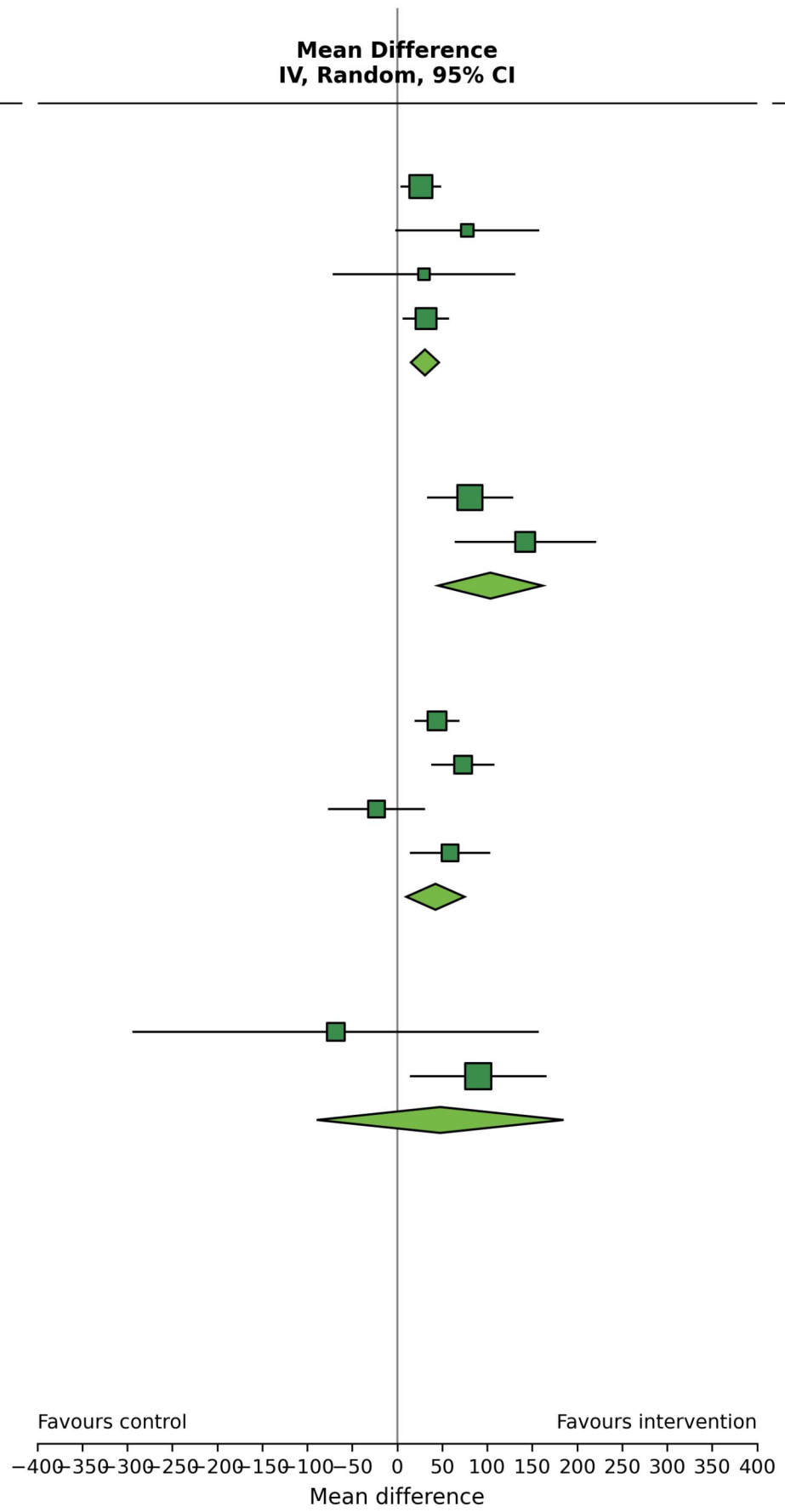

Figure S5.3. Pairwise meta-analysis forest plot for NT-proBNP

Traditional pairwise forest plots for NT-proBNP

Study or subgroup	Intervention Mean	Intervention SD	Intervention Total	Control Mean	Control SD	Control Total	Weight	Mean Difference IV, Random, 95% CI
**AE vs UC**								
Jia 2024	155.5	54.08	30	283.47	42.65	30	48.3%	-127.97 [-152.62, -103.32]
Yilmaz 2020	474.61	871.1	11	1181.03	1689	11	3.7%	-706.42 [-1829.49, 416.65]
Rakhmawati 2020	401.9	59.8	19	295.6	54	16	48.0%	106.30 [68.58, 144.02]
**Subtotal (95% CI)**			**60**			**57**	**100.0%**	**-37.15 [-262.59, 188.28]**
Heterogeneity: Tau² = 27216.47; I² = 98.1%	*Test for overall effect: Z = -0.32 (P = 0.7467)*							

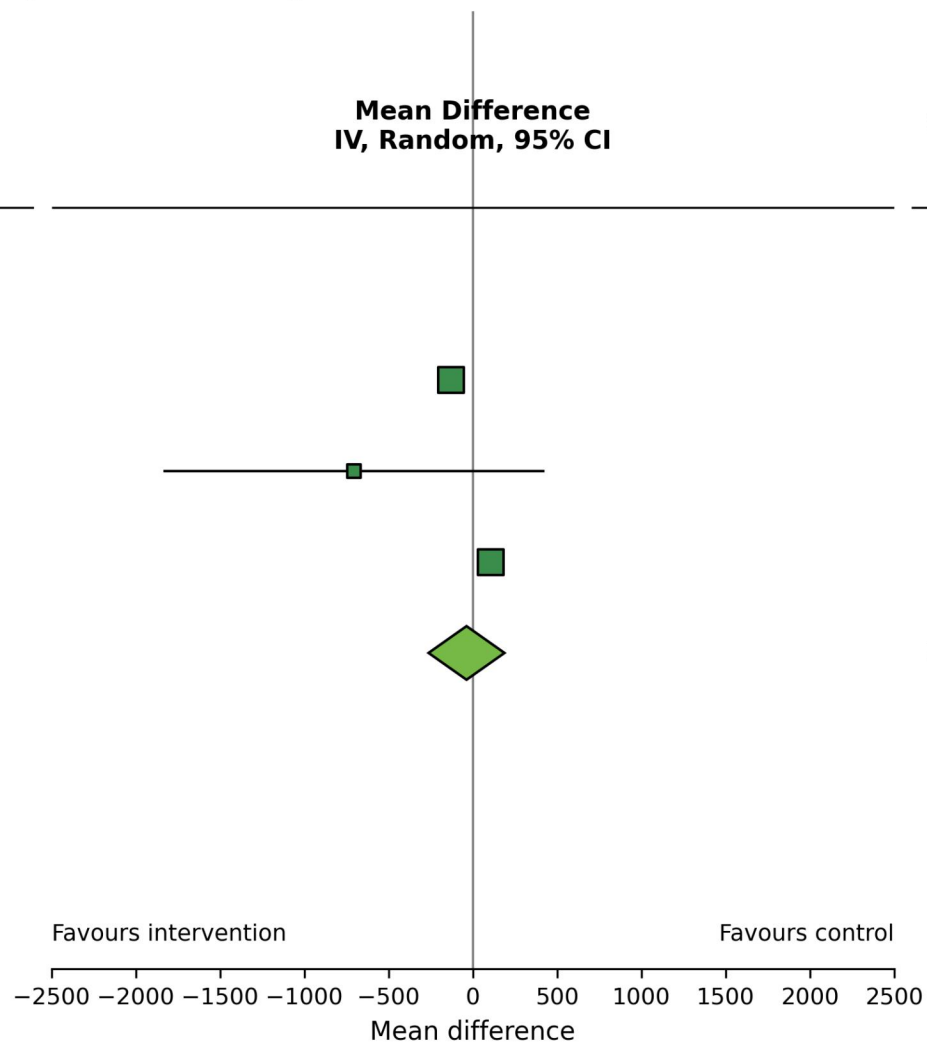

Figure S5.4. Pairwise meta-analysis forest plot for Peak VO_2

Traditional pairwise forest plots for Peak VO2

Study or subgroup	Intervention Mean	Intervention SD	Intervention Total	Control Mean	Control SD	Control Total	Weight	Mean Difference IV, Random, 95% CI
**AE vs UC**								
Zhang 2025	22.7	3	26	17.2	3.3	28	54.2%	5.50 [3.82, 7.18]
Chan 2013	18.9	10.3	10	15.1	5.7	13	12.9%	3.80 [-3.30, 10.90]
Yilmaz 2020	14.38	4.25	11	12.8	4.15	11	33.0%	1.58 [-1.93, 5.09]
**Subtotal (95% CI)**			**47**			**52**	**100.0%**	**3.99 [1.16, 6.82]**
Heterogeneity: Tau² = 3.12; I² = 49.6%		*Test for overall effect: Z = 2.76 (P = 0.0057)*						
**AE+BT vs UC**								
Ehlken 2016	16.4	4.5	38	12.5	4.6	41	61.0%	3.90 [1.89, 5.91]
Mereles 2006	15.4	3.7	15	11.4	3.3	15	39.0%	4.00 [1.49, 6.51]
**Subtotal (95% CI)**			**53**			**56**	**100.0%**	**3.94 [2.37, 5.51]**
Heterogeneity: Tau² = 0.00; I² = 0.0%		*Test for overall effect: Z = 4.93 (P = <0.0001)*						
**AE+RE vs UC**								
Kagioglou 2021	18.4	5.6	12	14.5	2.6	10	73.6%	3.90 [0.35, 7.45]
Tran 2021	16.3	5	5	15.3	5	6	26.4%	1.00 [-4.93, 6.93]
**Subtotal (95% CI)**			**17**			**16**	**100.0%**	**3.13 [0.08, 6.18]**
Heterogeneity: Tau² = 0.00; I² = 0.0%		*Test for overall effect: Z = 2.01 (P = 0.044)*						

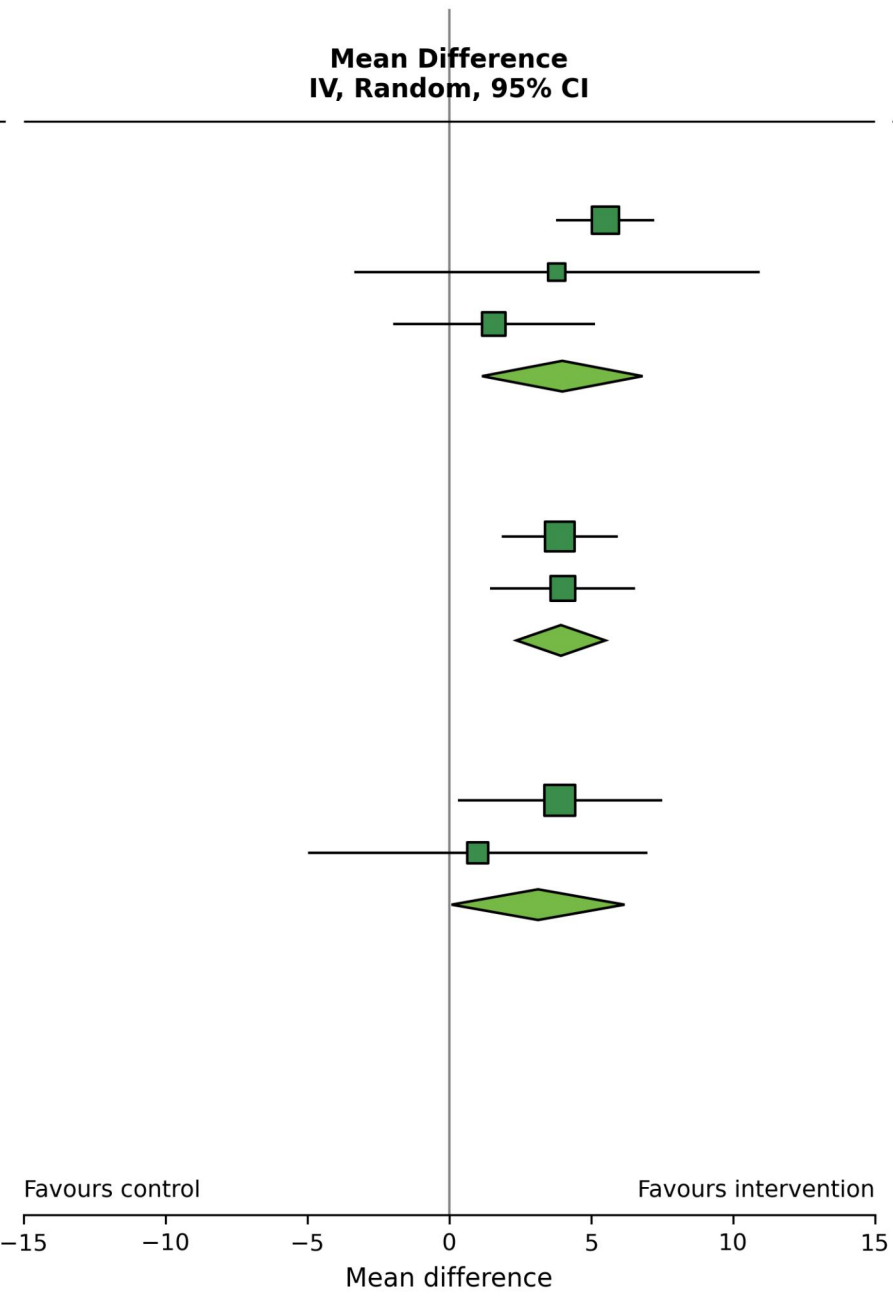

Figure S5.5. Pairwise meta-analysis forest plot for $FEV_1\%$

Traditional pairwise forest plots for FEV1%

Study or subgroup	Intervention Mean	Intervention SD	Intervention Total	Control Mean	Control SD	Control Total	Weight	Mean Difference IV, Random, 95% CI
**Breathing-focused intervention vs UC**								
Jia 2019	78.53	5.9	23	73.12	5.37	26	90.7%	5.41 [2.24, 8.58]
Saglam 2015	80.43	21.26	14	65.67	24.97	15	9.3%	14.76 [-2.08, 31.60]
**Subtotal (95% CI)**			**37**			**41**	**100.0%**	**6.28 [0.96, 11.59]**
Heterogeneity: Tau² = 5.47; I² = 12.5%		*Test for overall effect: Z = 2.32 (P = 0.0206)*						

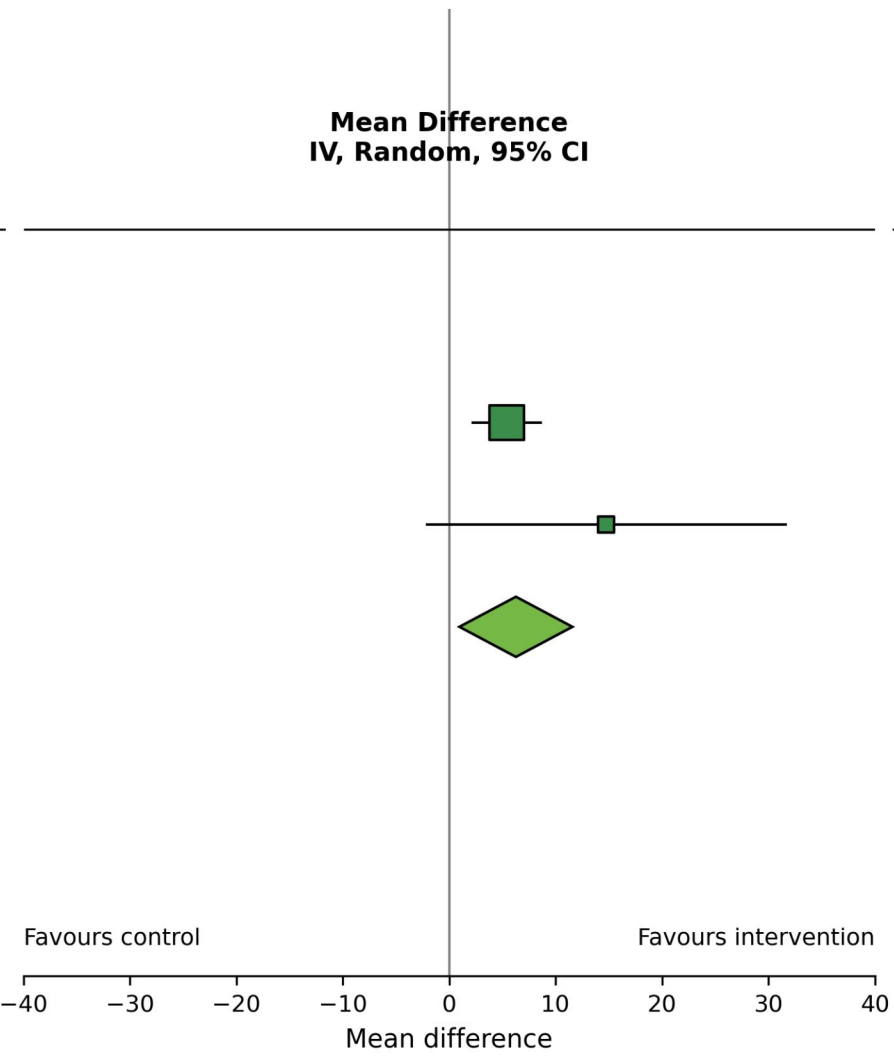

Figure S5.6. Pairwise meta-analysis forest plot for HR rest

Traditional pairwise forest plots for HR rest

Study or subgroup	Intervention Mean	Intervention SD	Intervention Total	Control Mean	Control SD	Control Total	Weight	Mean Difference IV, Random, 95% CI
**AE vs UC**								
Yilmaz 2020	92.93	8.84	11	83.36	19.3	11	24.8%	9.57 [-2.98, 22.12]
Hemnes 2021	70	4.5	20	69	8	22	75.2%	1.00 [-2.88, 4.88]
**Subtotal (95% CI)**			**31**			**33**	**100.0%**	**3.12 [-4.13, 10.38]**
Heterogeneity: Tau² = 14.28; I² = 38.9%	*Test for overall effect: Z = 0.84 (P = 0.3985)*							
**AE+BT vs UC**								
Ehlken 2016	76	15	38	76	16	41	57.0%	0.00 [-6.84, 6.84]
Mereles 2006	75	11	15	72	11	15	43.0%	3.00 [-4.87, 10.87]
**Subtotal (95% CI)**			**53**			**56**	**100.0%**	**1.29 [-3.87, 6.45]**
Heterogeneity: Tau² = 0.00; I² = 0.0%	*Test for overall effect: Z = 0.49 (P = 0.6243)*							

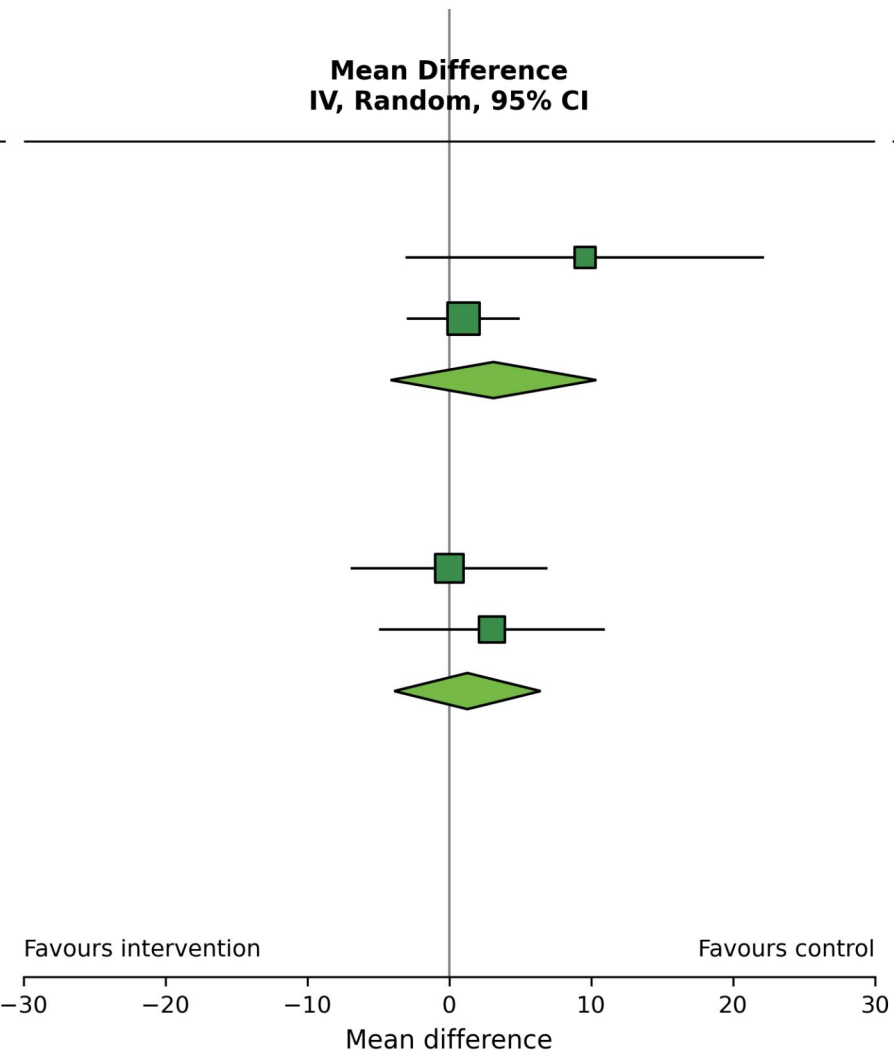

Figure S5.7. Pairwise meta-analysis forest plot for HR max

Traditional pairwise forest plots for HR max

Study or subgroup	Intervention Mean	Intervention SD	Intervention Total	Control Mean	Control SD	Control Total	Weight	Mean Difference IV, Random, 95% CI
**AE vs UC**								
Zhang 2025	161.2	8.3	26	156.9	7.3	28	77.5%	4.30 [0.12, 8.48]
Yilmaz 2020	145.81	21.46	11	127.66	27.39	11	22.5%	18.15 [-2.41, 38.71]
**Subtotal (95% CI)**			**37**			**39**	**100.0%**	**7.42 [-3.92, 18.75]**
Heterogeneity: Tau² = 38.60; I² = 40.2%	*Test for overall effect: Z = 1.28 (P = 0.1997)*							
**AE+BT vs UC**								
Ehlken 2016	136	26	38	121	21	41	61.7%	15.00 [4.53, 25.47]
Mereles 2006	132	17	15	110	20	15	38.3%	22.00 [8.72, 35.28]
**Subtotal (95% CI)**			**53**			**56**	**100.0%**	**17.68 [9.46, 25.91]**
Heterogeneity: Tau² = 0.00; I² = 0.0%	*Test for overall effect: Z = 4.21 (P = <0.0001)*							
**AE+RE vs UC**								
Martinez-Quintana 2010	98.7	12.1	4	95.5	20.9	4	5.4%	3.20 [-20.47, 26.87]
Kagioglou 2021	153.3	19.7	12	159.5	25	10	8.3%	-6.20 [-25.29, 12.89]
Tran 2021	132	5	5	126	5	6	86.2%	6.00 [0.07, 11.93]
**Subtotal (95% CI)**			**21**			**20**	**100.0%**	**4.83 [-0.68, 10.34]**
Heterogeneity: Tau² = 0.00; I² = 0.0%	*Test for overall effect: Z = 1.72 (P = 0.0857)*							

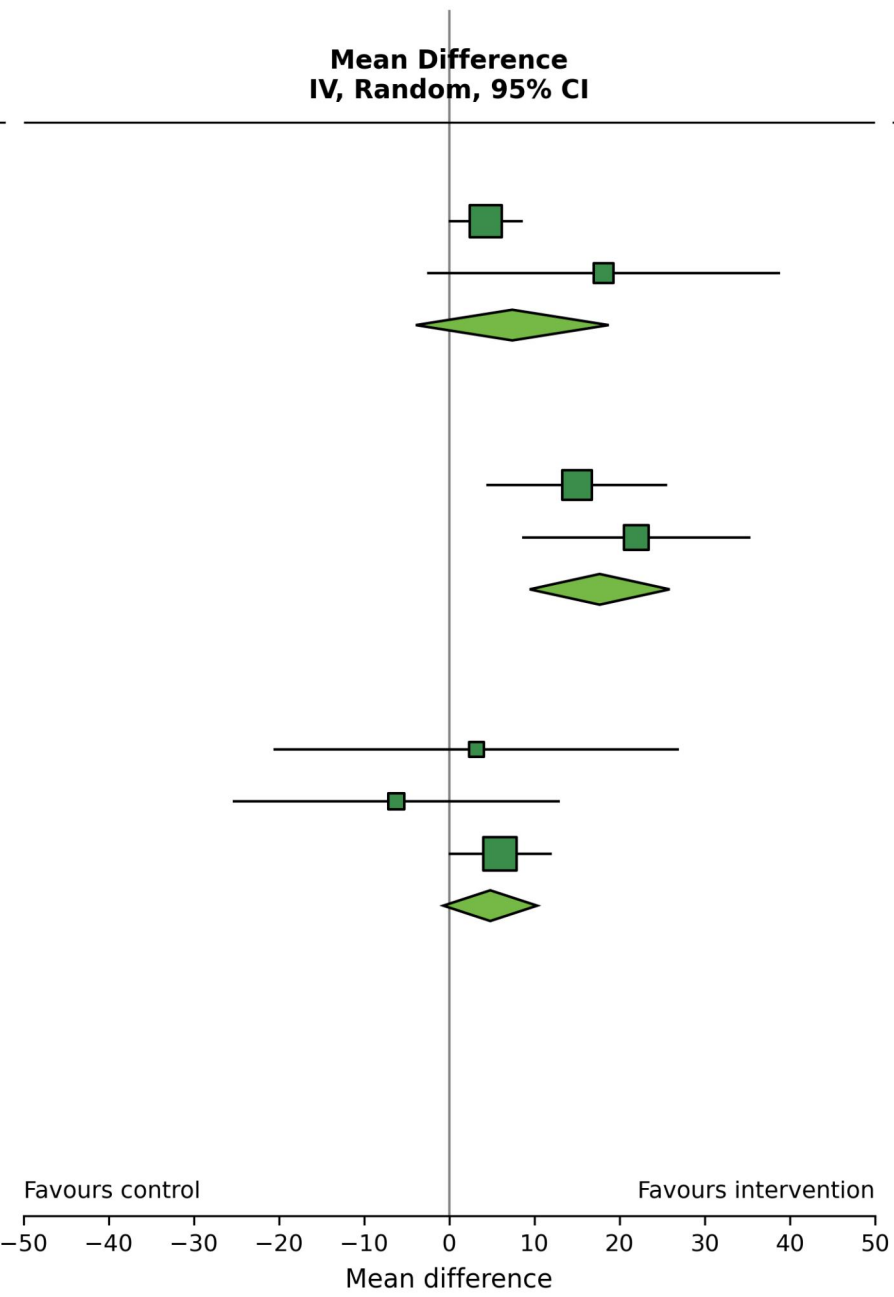

Appendix 6: Evaluation of consistency and heterogeneity

Table S6.1: Evaluation of consistency and heterogeneity

Parameters	Outcomes	Study	Consistency
			I^2
Indicators	High intensity	3 studies, n = 99	3%
	Medium intensity	4 studies, n =113	6%
	Low intensity	5 studies, n = 264	7%
	＜3 times/week	1 studies, n =8	9%
	3-5 times/week	9 studies, n = 325	3%
	＞5 times/week	6 studies, n =281	4%

Table S6.2

Parameters	Outcomes	Study	Consistency				
			Totresdev	pD	DIC	I^2	τ^2
Indicators	SF-36	6 studies, n = 248	30.14521	27.81234	57.95755	6%	0.003
	6MWD	13 studies, n = 495	46.20984	42.10358	88.47619	5%	0.004
	NT-proBNP	5 studies, n = 232	17.59321	16.43219	34.20976	8%	0.006
	Peak VO_2	9 studies, n = 295	7.95162	7.40215	15.20988	6%	0.005

Note: τ^2 represents the between-study variance estimated under the random-effects model for each outcome.

Appendix 7: Trace plots and density plots of each outcome

Figure S7.1: Density plots and Trajectory plots of **SF-36**

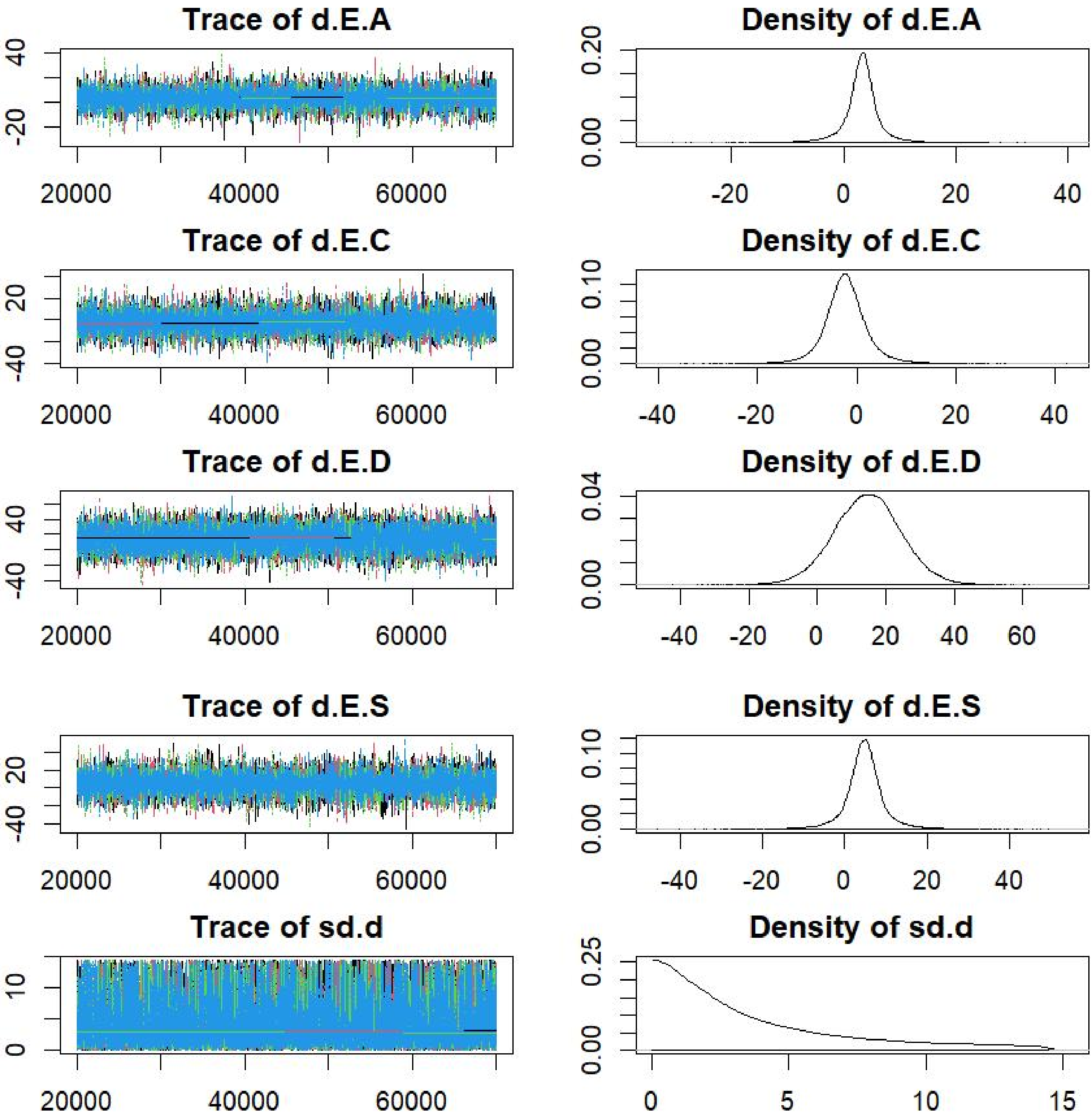

Figure S7.2: Density plots and Trajectory plots of **6MWD**

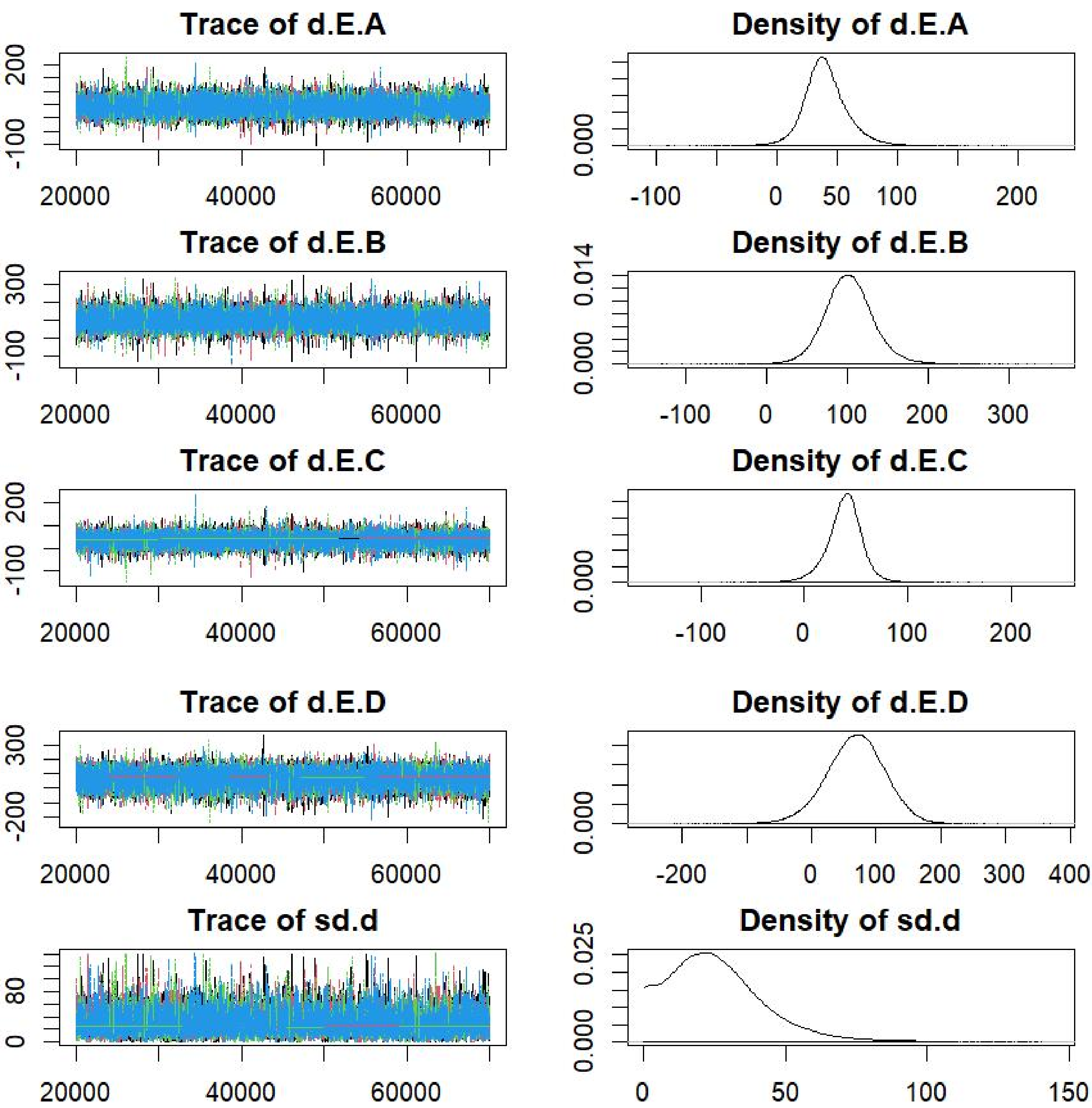

Figure S7.3: Density plots and Trajectory plots of **NT-proBNP**

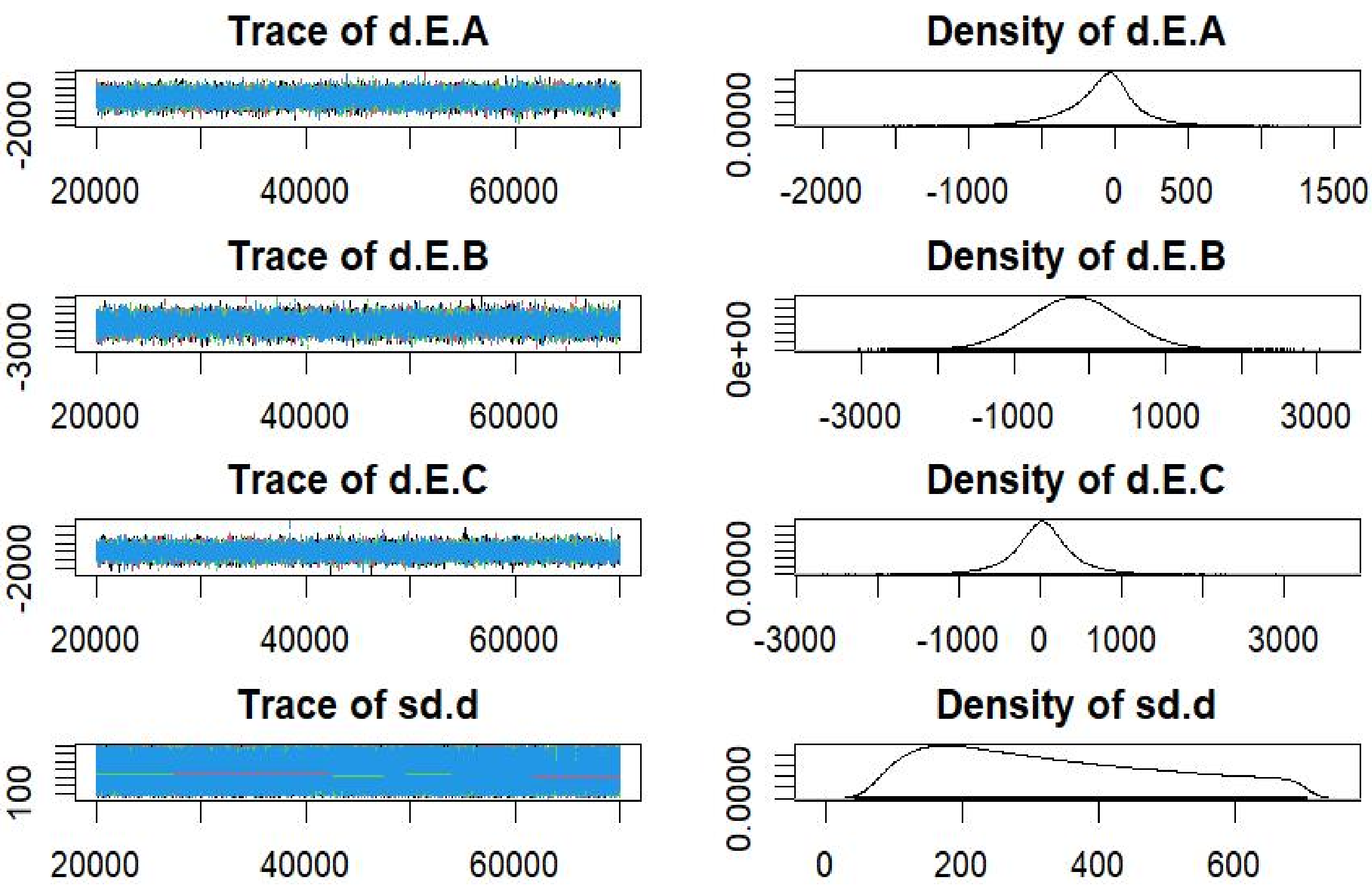

Figure S7.4: Density plots and Trajectory plots of **Peak VO_2**

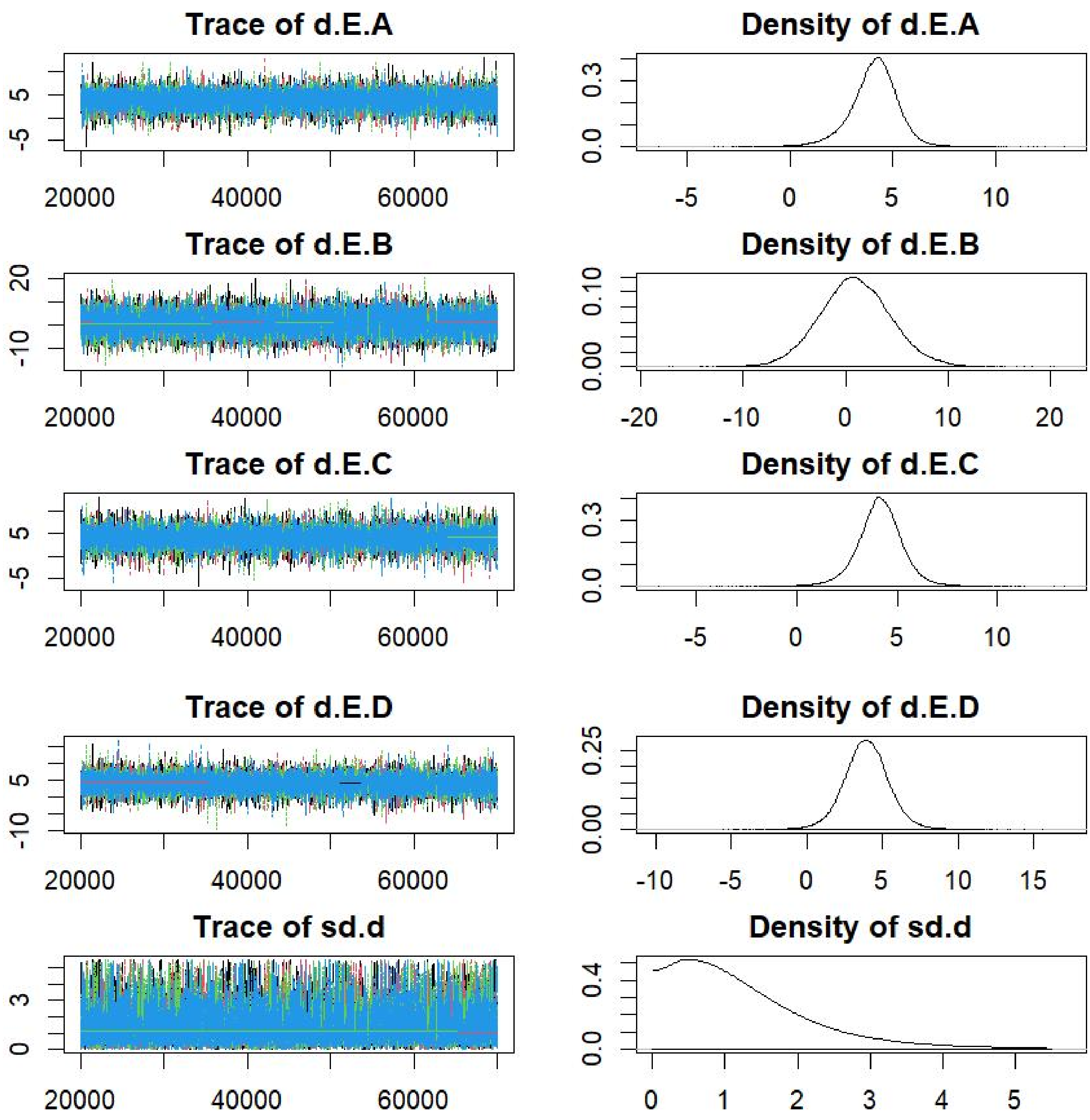

Figure S7.5: Density plots and Trajectory plots of $\mathbf{FEV_1\%}$

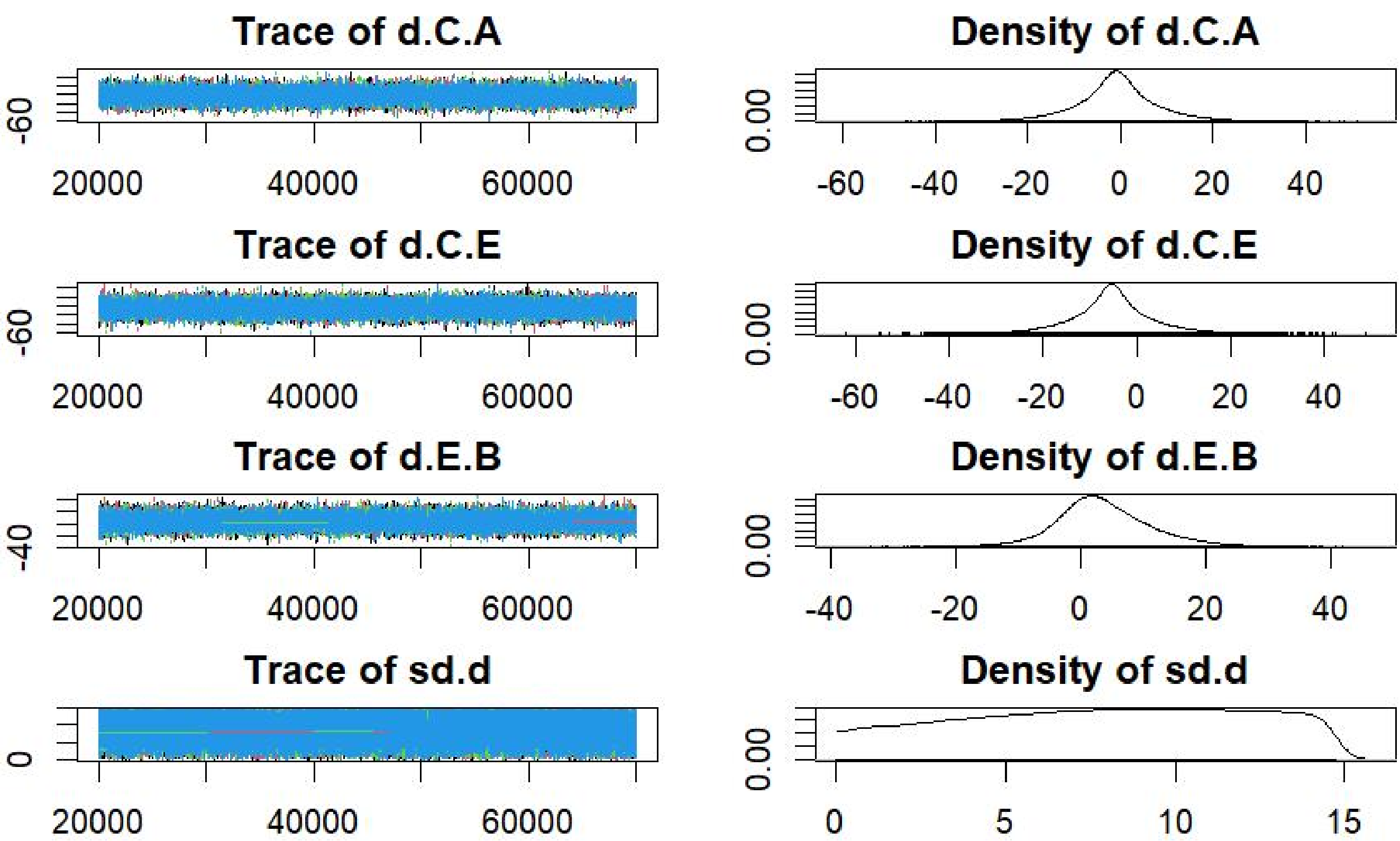

Figure S7.6: Density plots and Trajectory plots of **HR rest**

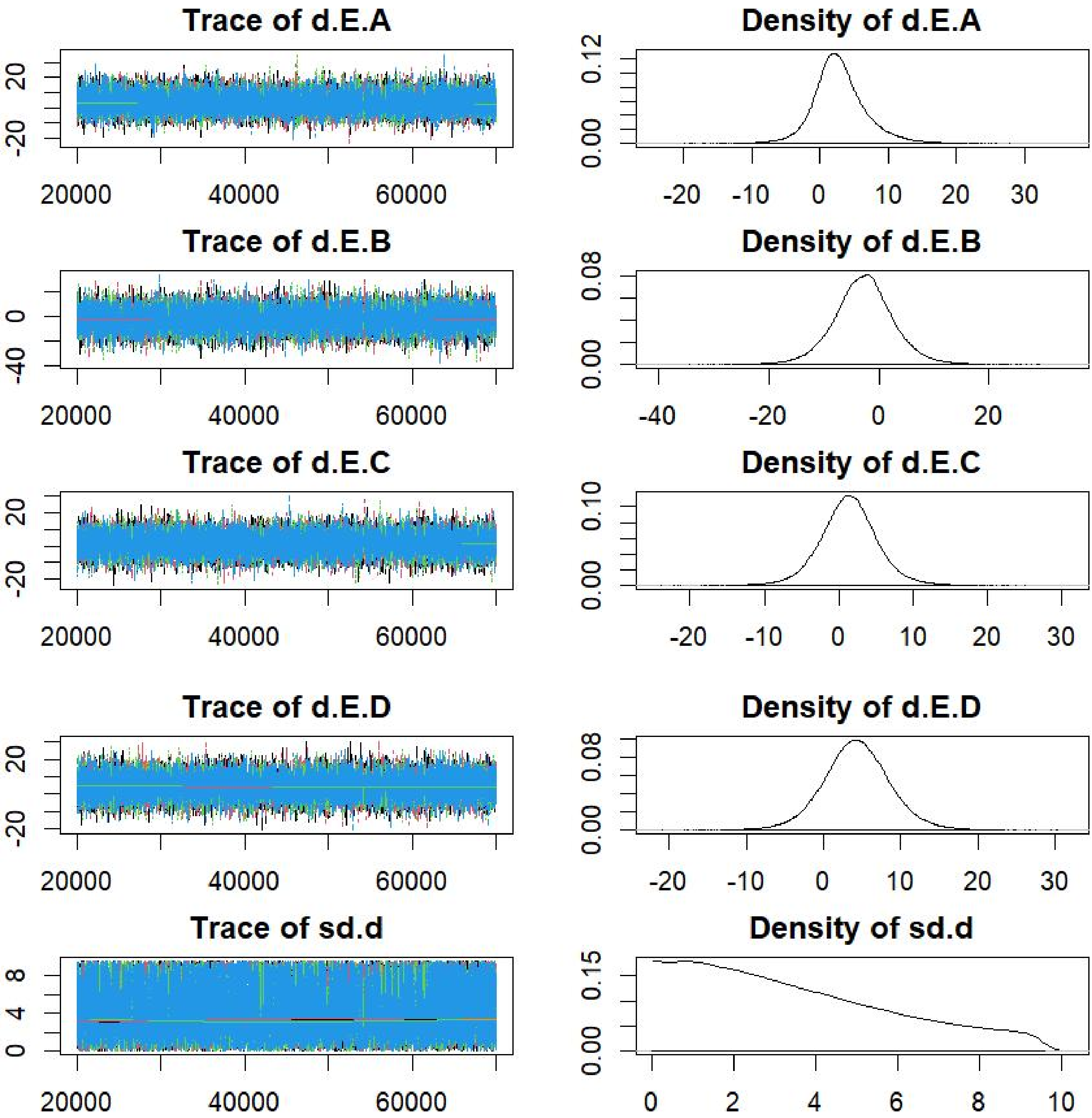

Figure S7.7: Density plots and Trajectory plots of **HR max**

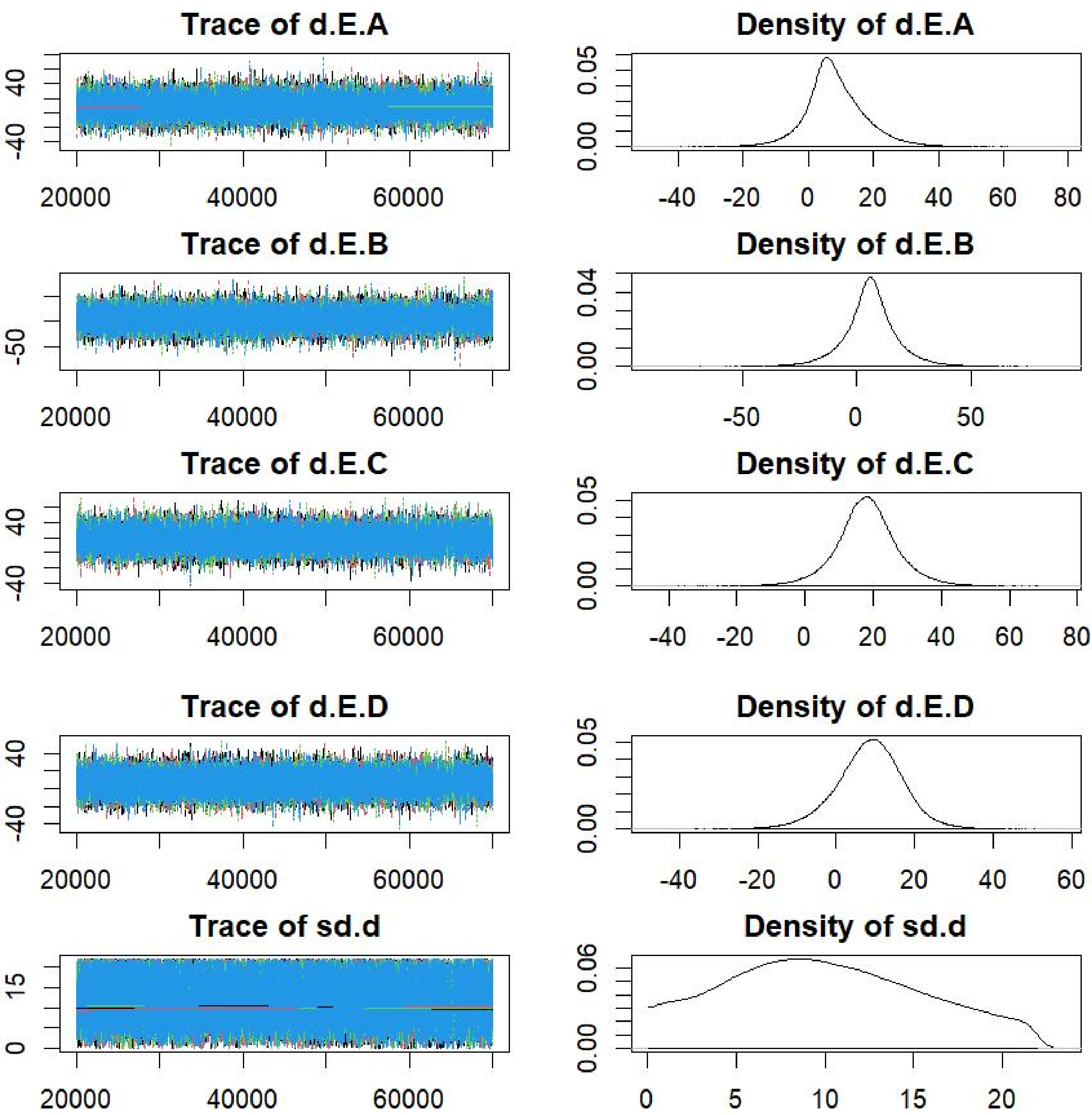

Appendix 8: Gelman-Rubin and autocorrelation diagnostic plots of each outcome

Figure S8.1: Gelman-Rubin and autocorrelation diagnostic plots for **SF-36**

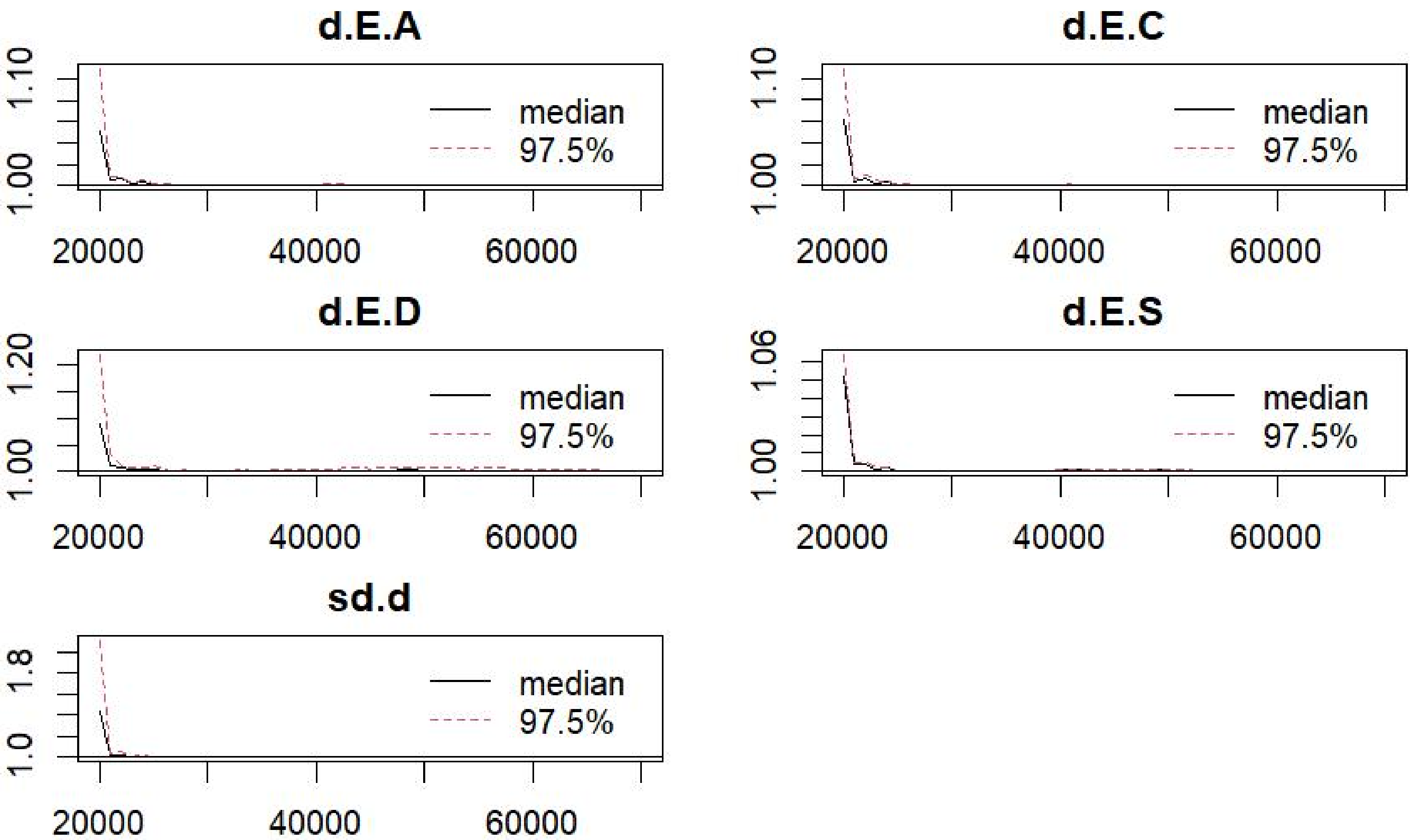

Figure S8.2: Gelman-Rubin and autocorrelation diagnostic plots for **6MWD**

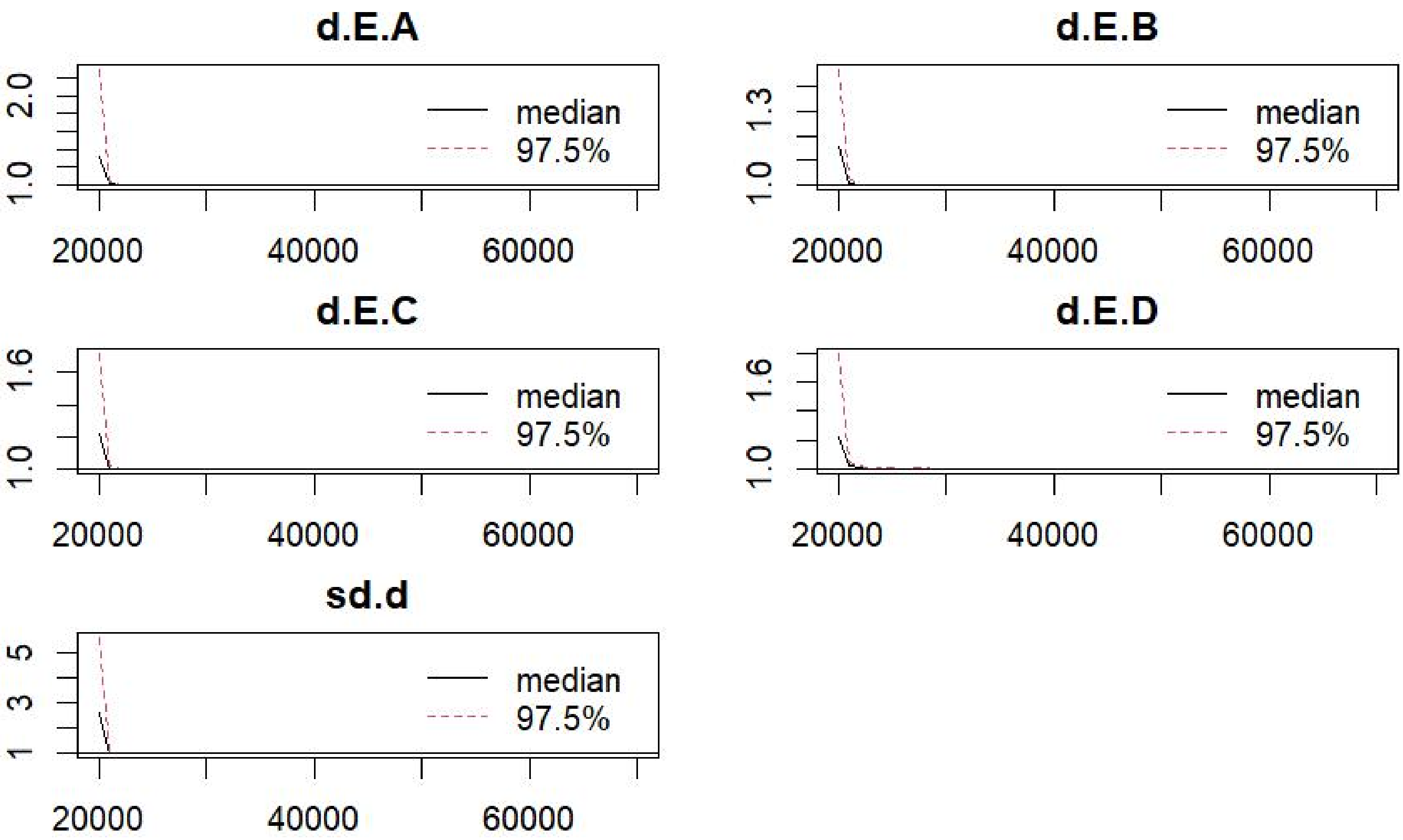

Figure S8.3: Gelman-Rubin and autocorrelation diagnostic plots for **NT-proBNP**

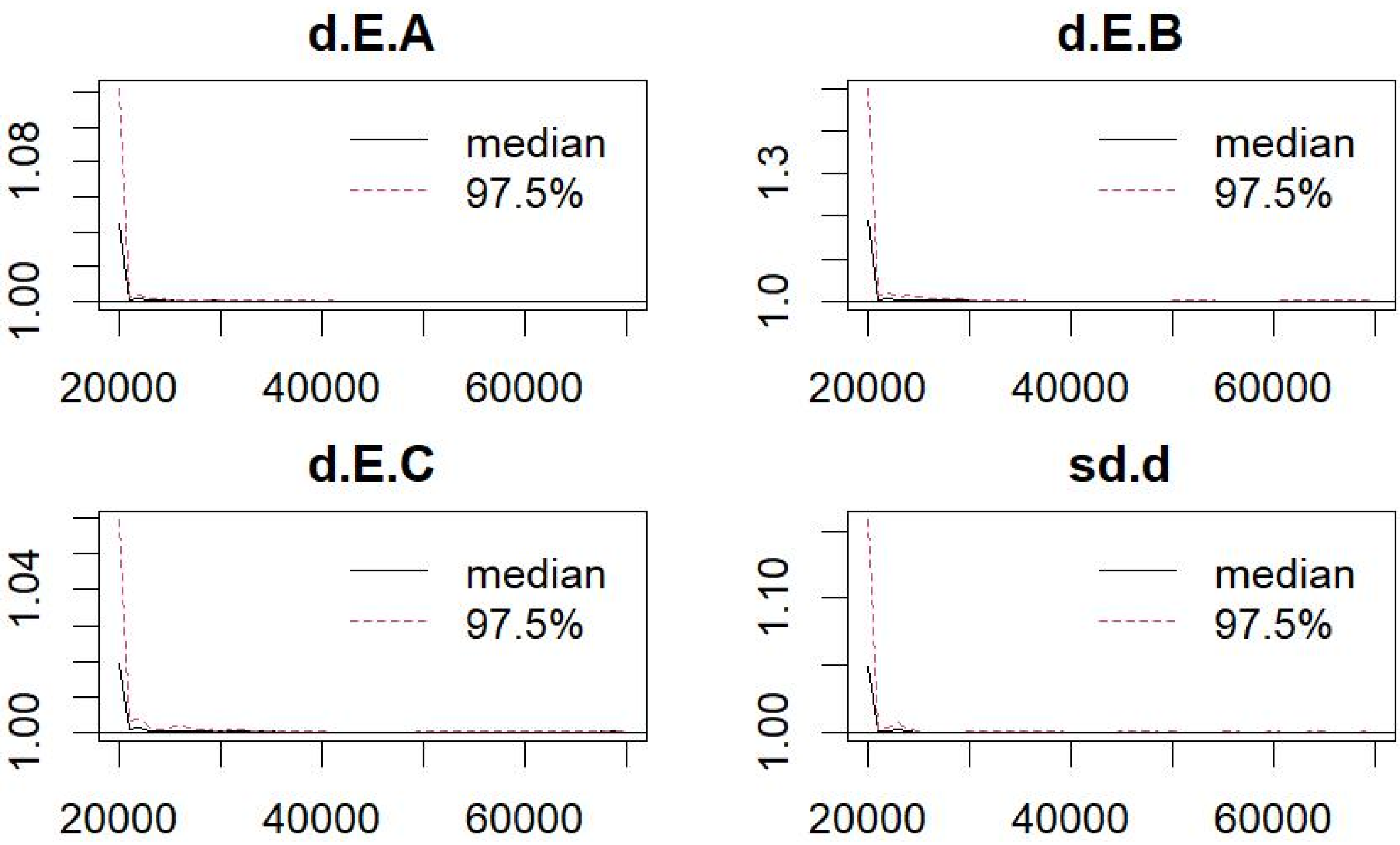

Figure S8.4: Gelman-Rubin and autocorrelation diagnostic plots for **Peak VO_2**

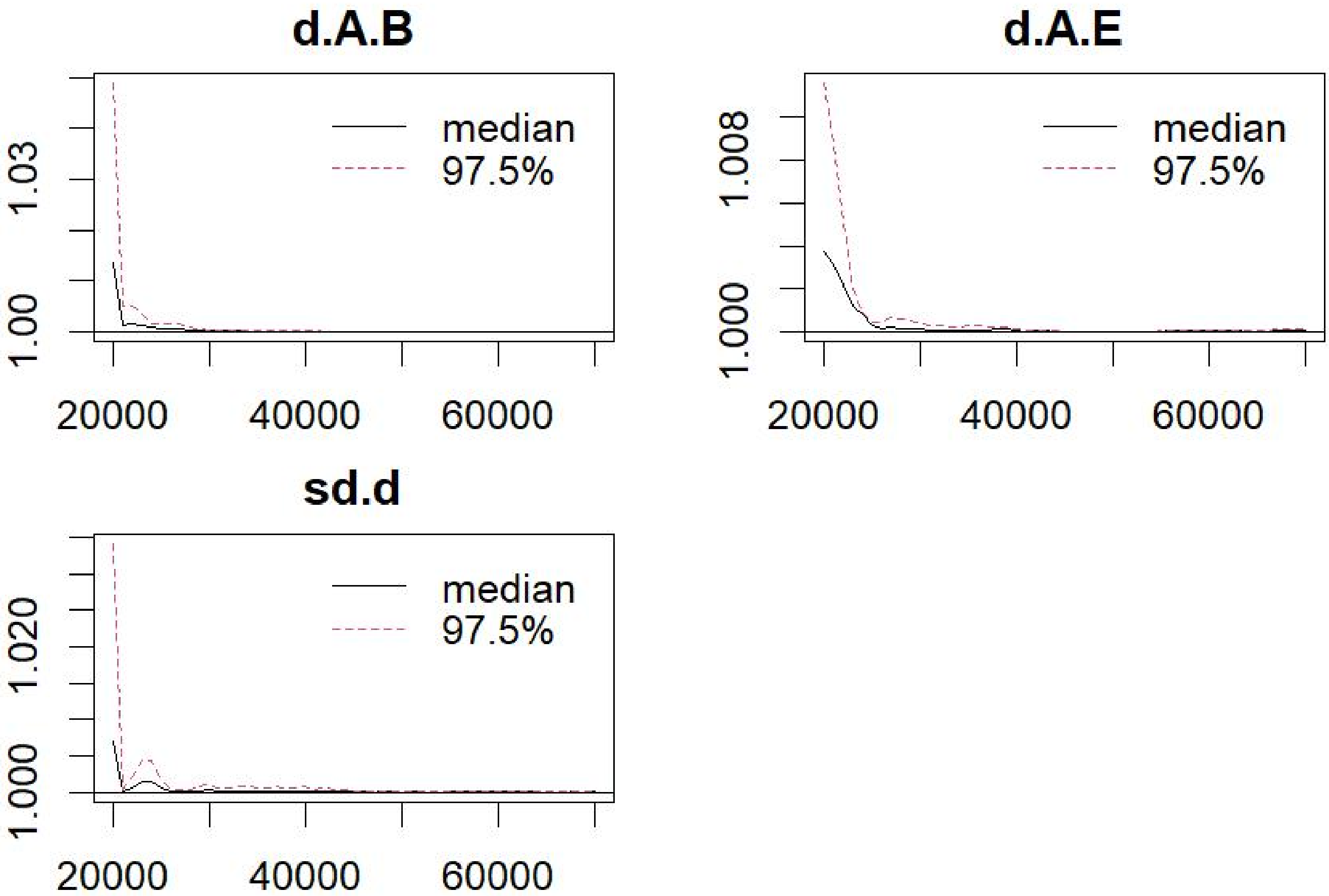

Figure S8.5: Gelman-Rubin and autocorrelation diagnostic plots for **FEV$_1$%**

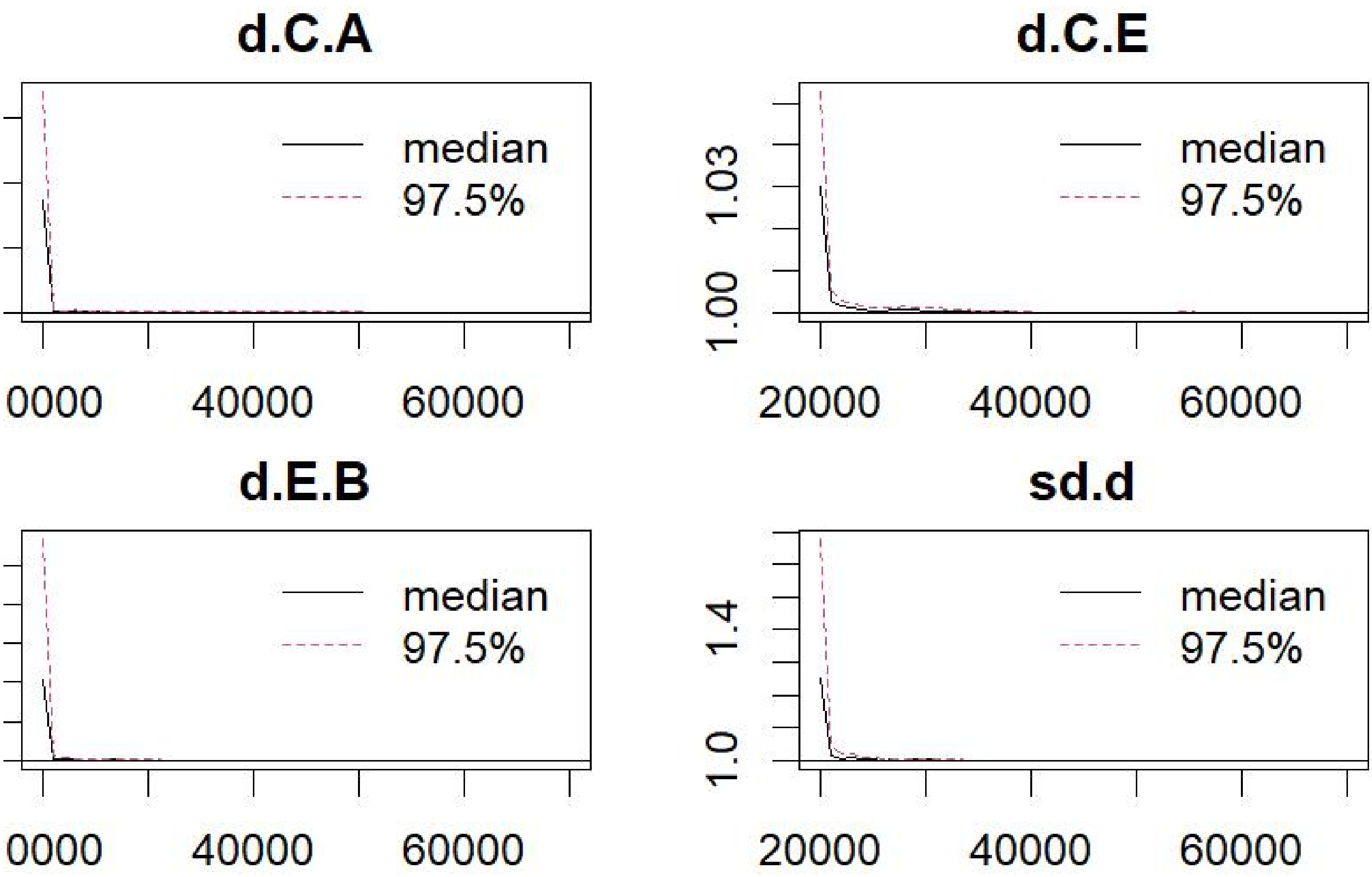

Figure S8.6: Gelman-Rubin and autocorrelation diagnostic plots for **HR rest**

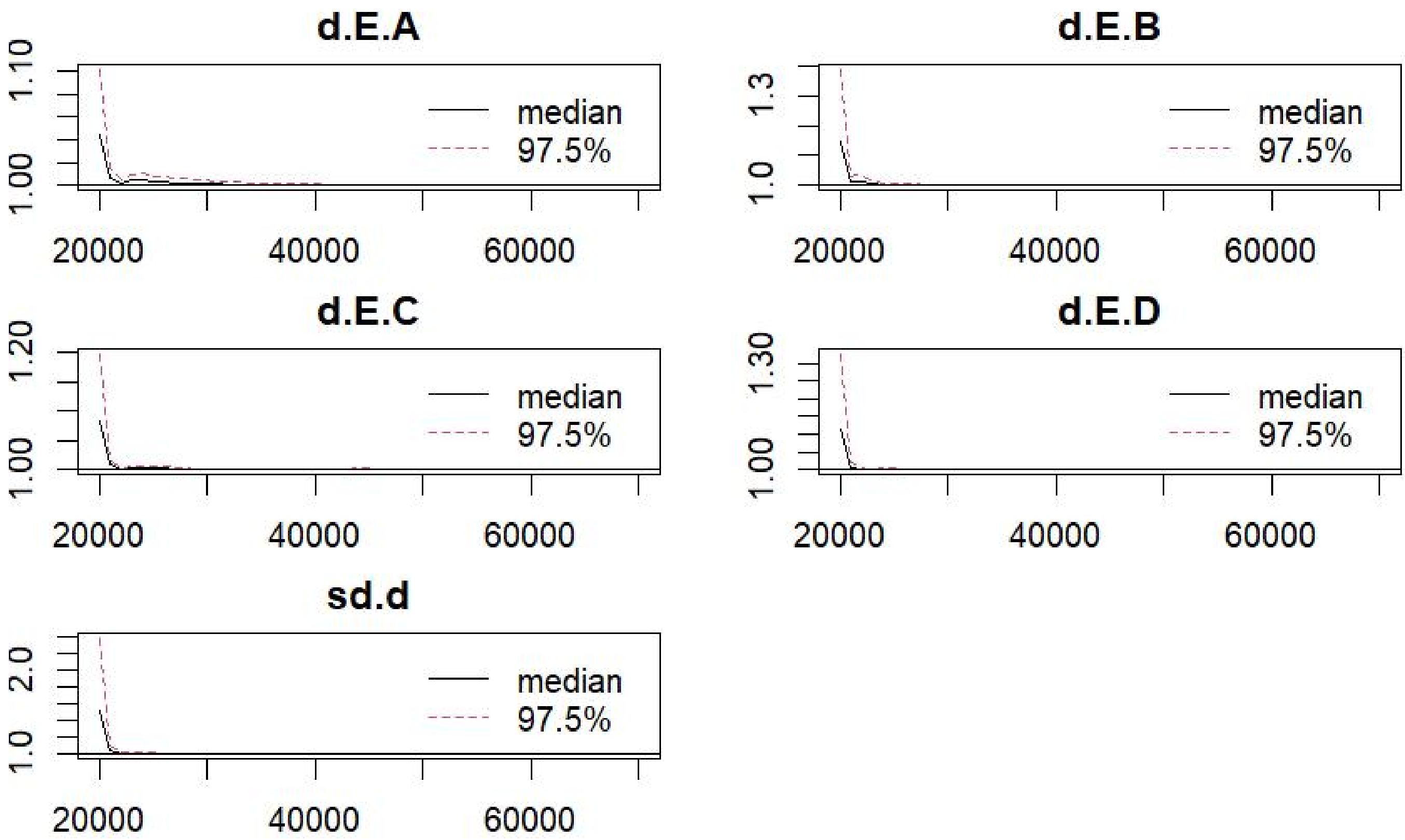

Figure S8.7: Gelman-Rubin and autocorrelation diagnostic plots for **HR max**

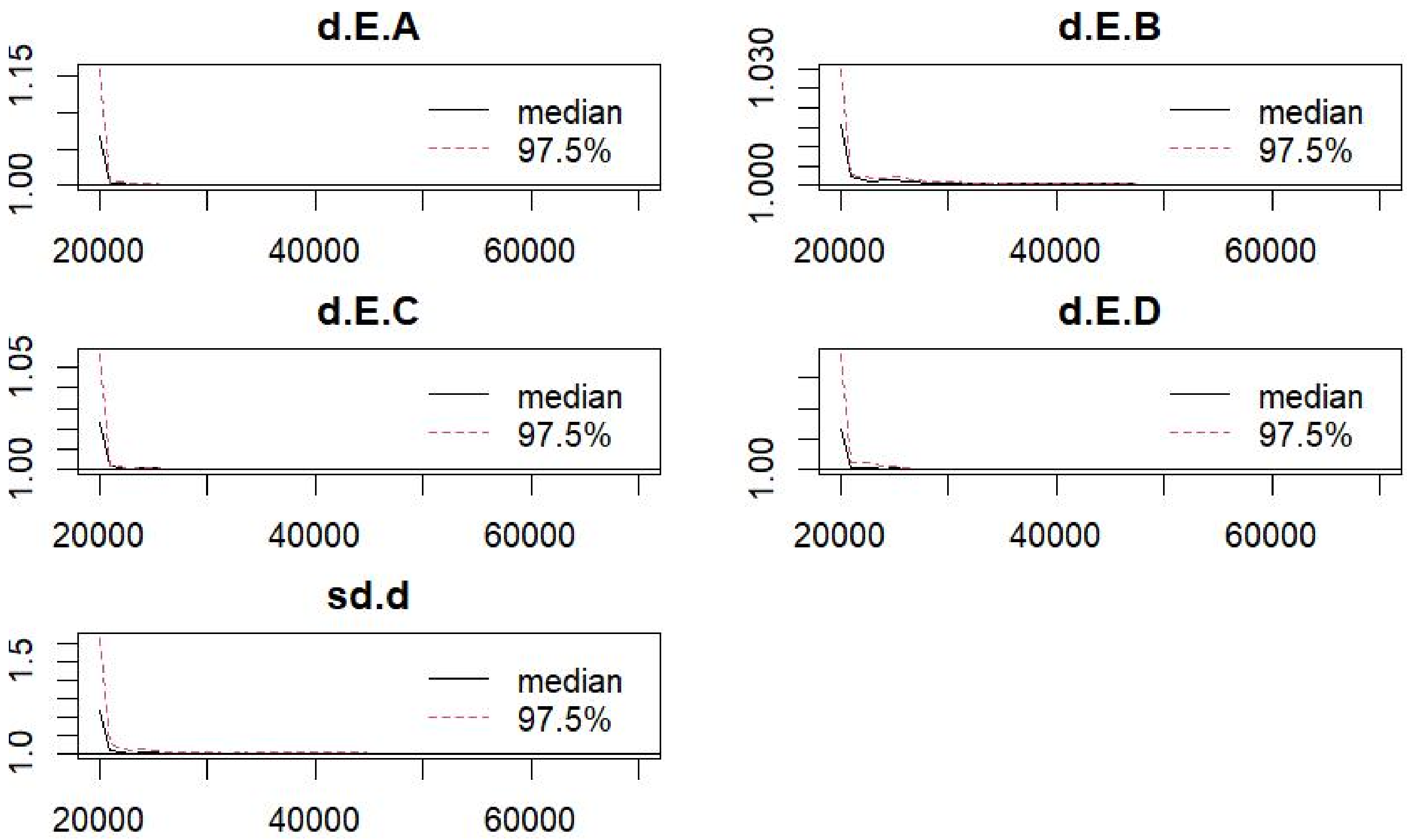

Appendix 9: Funnel plots

Figure S9.1: Funnel plot of **SF-36**

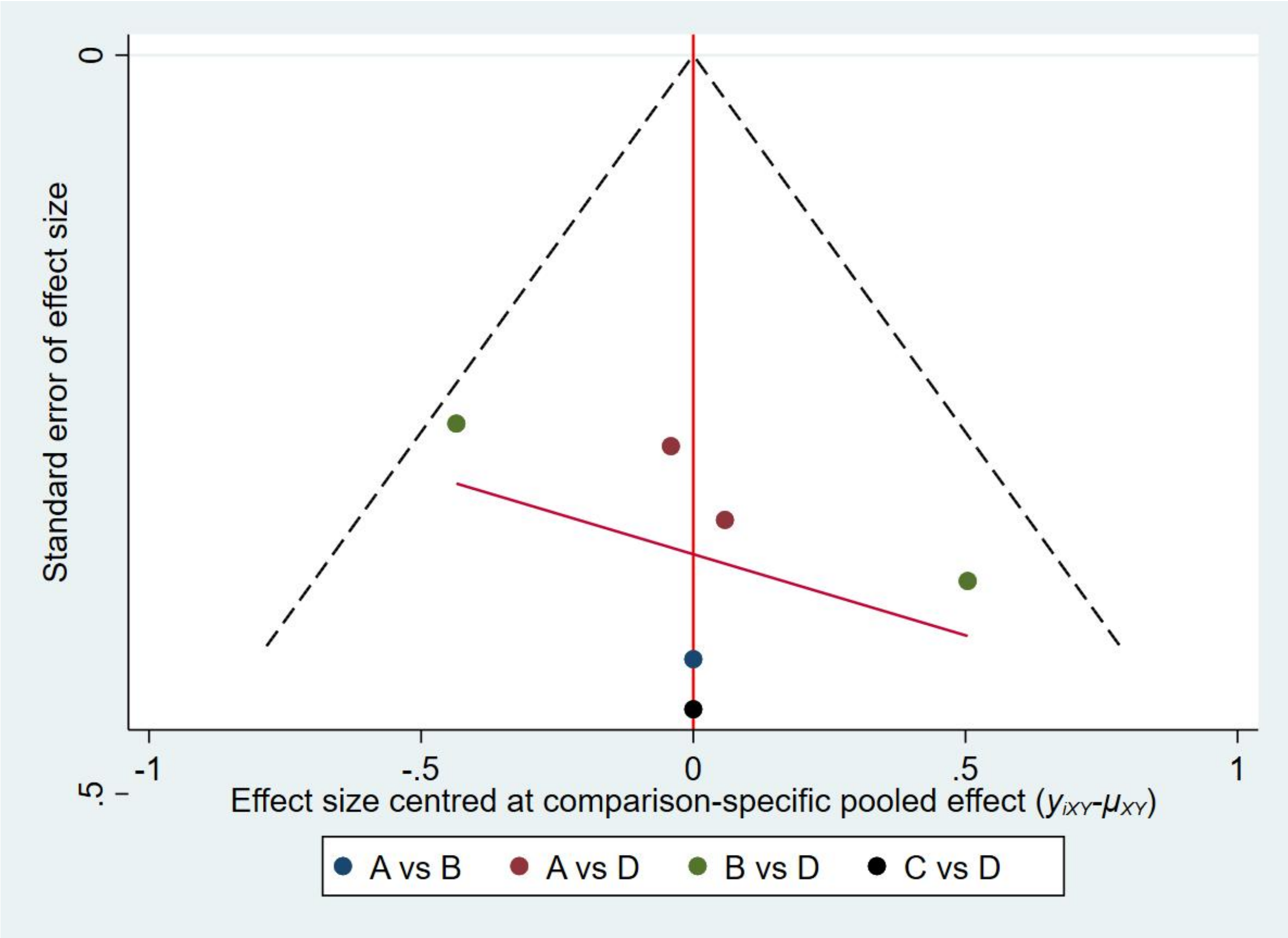

Figure S9.2: Funnel plot of **6MWD**

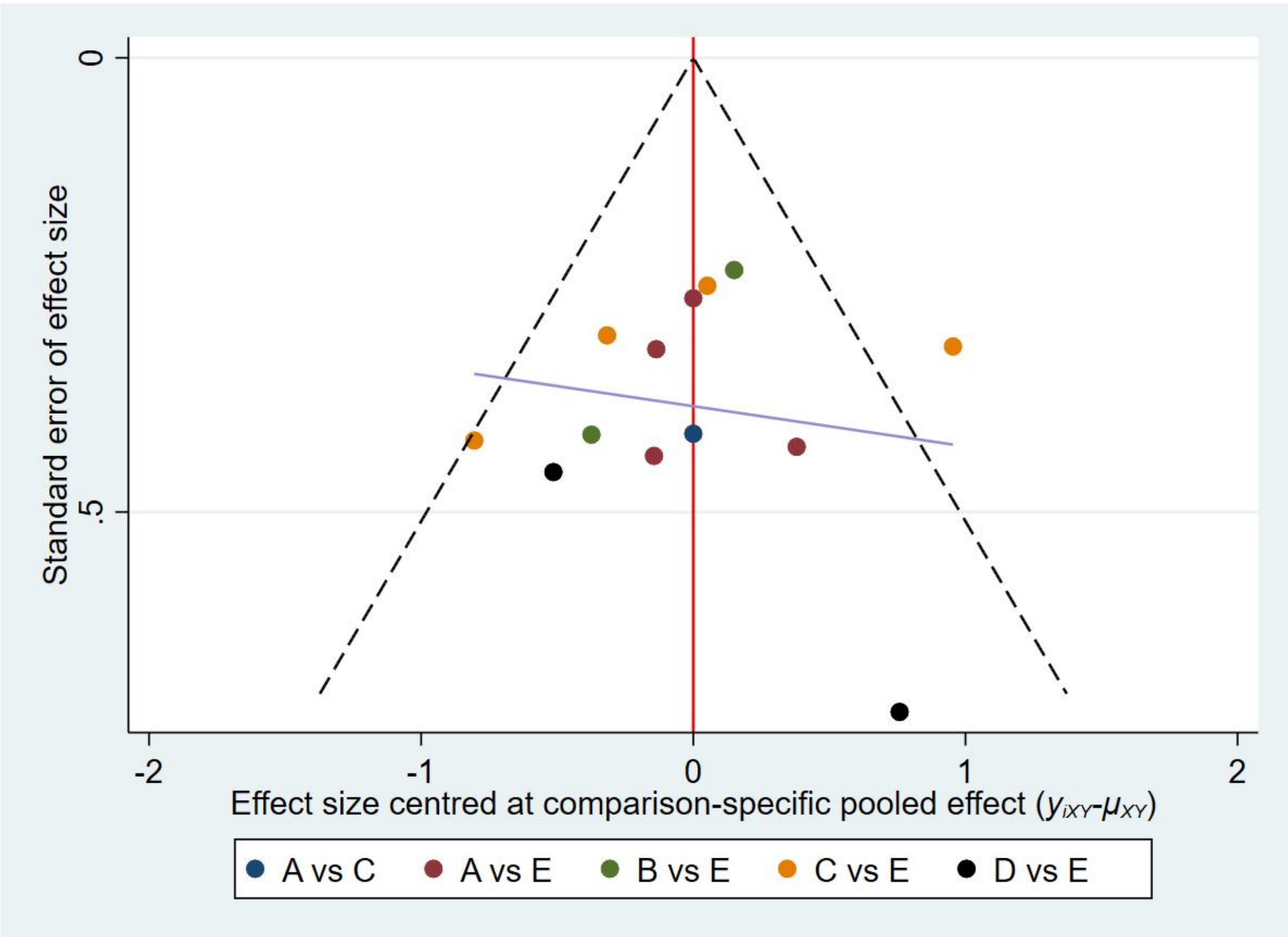

Figure S9.3: Funnel plot of **NT-proBNP**

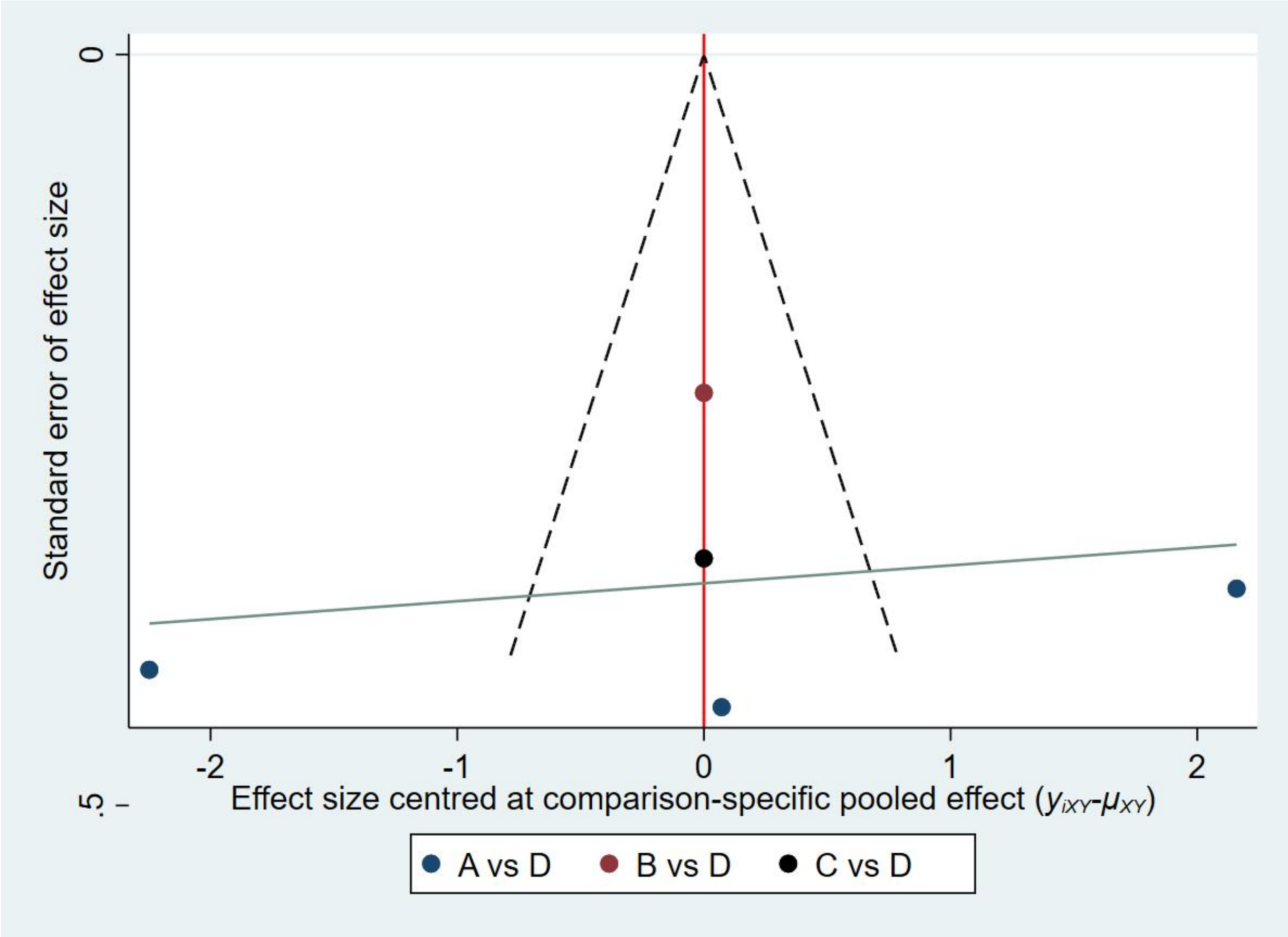

Figure S9.4: Funnel plot of **Peak VO_2**

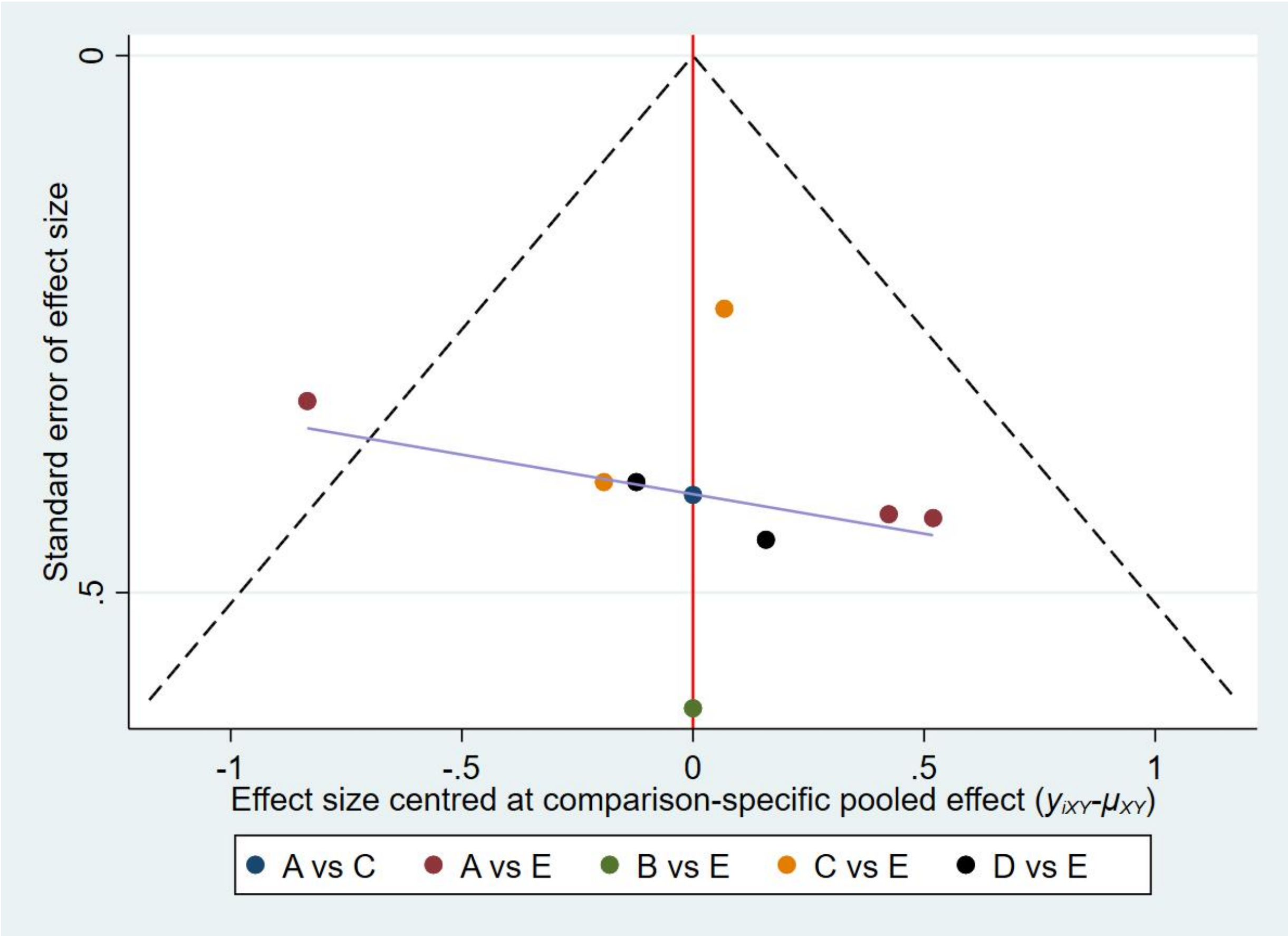

Figure S9.5: Funnel plot of **FEV$_1$%**

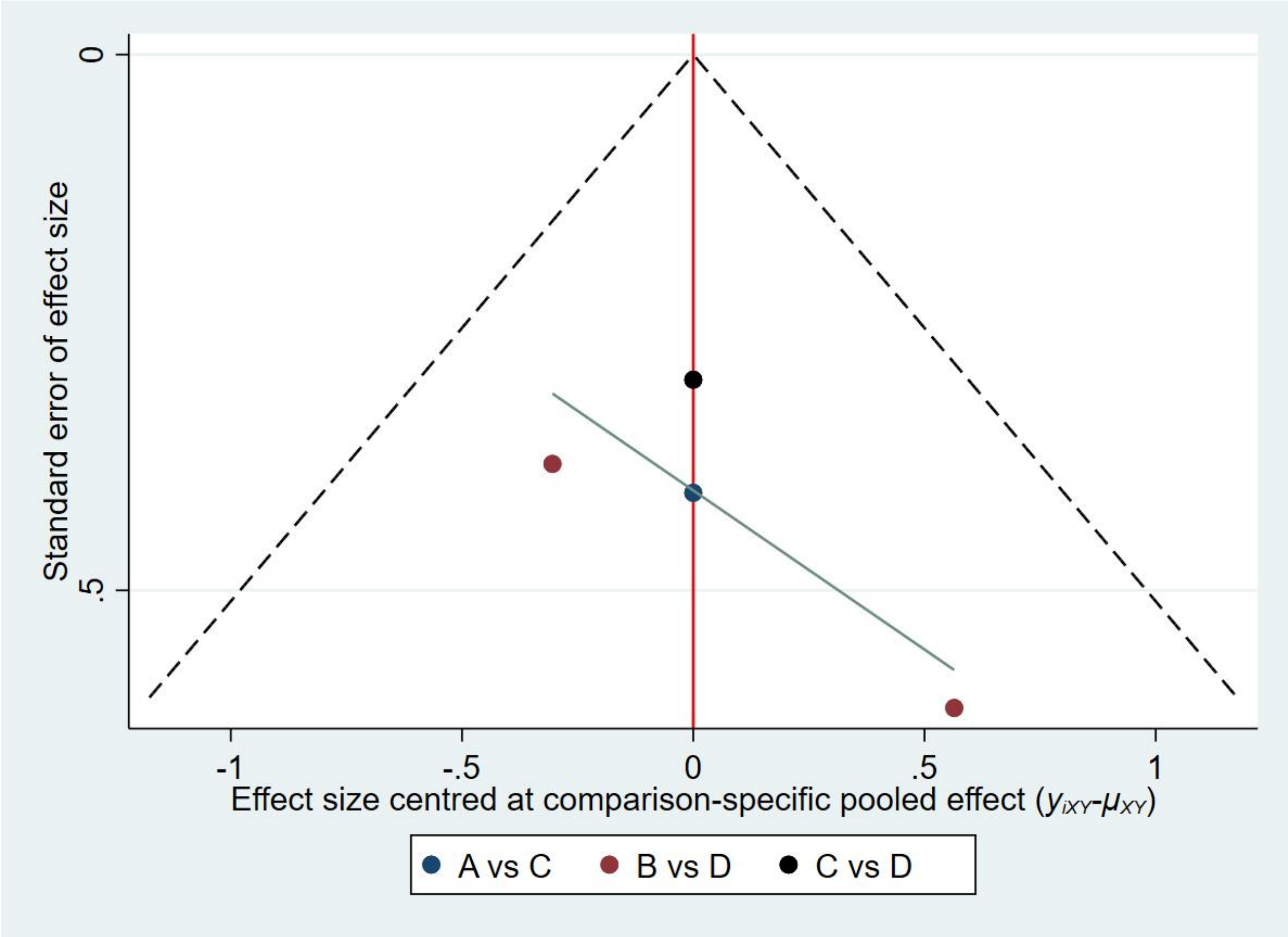

Figure S9.6: Funnel plot of **HR rest**

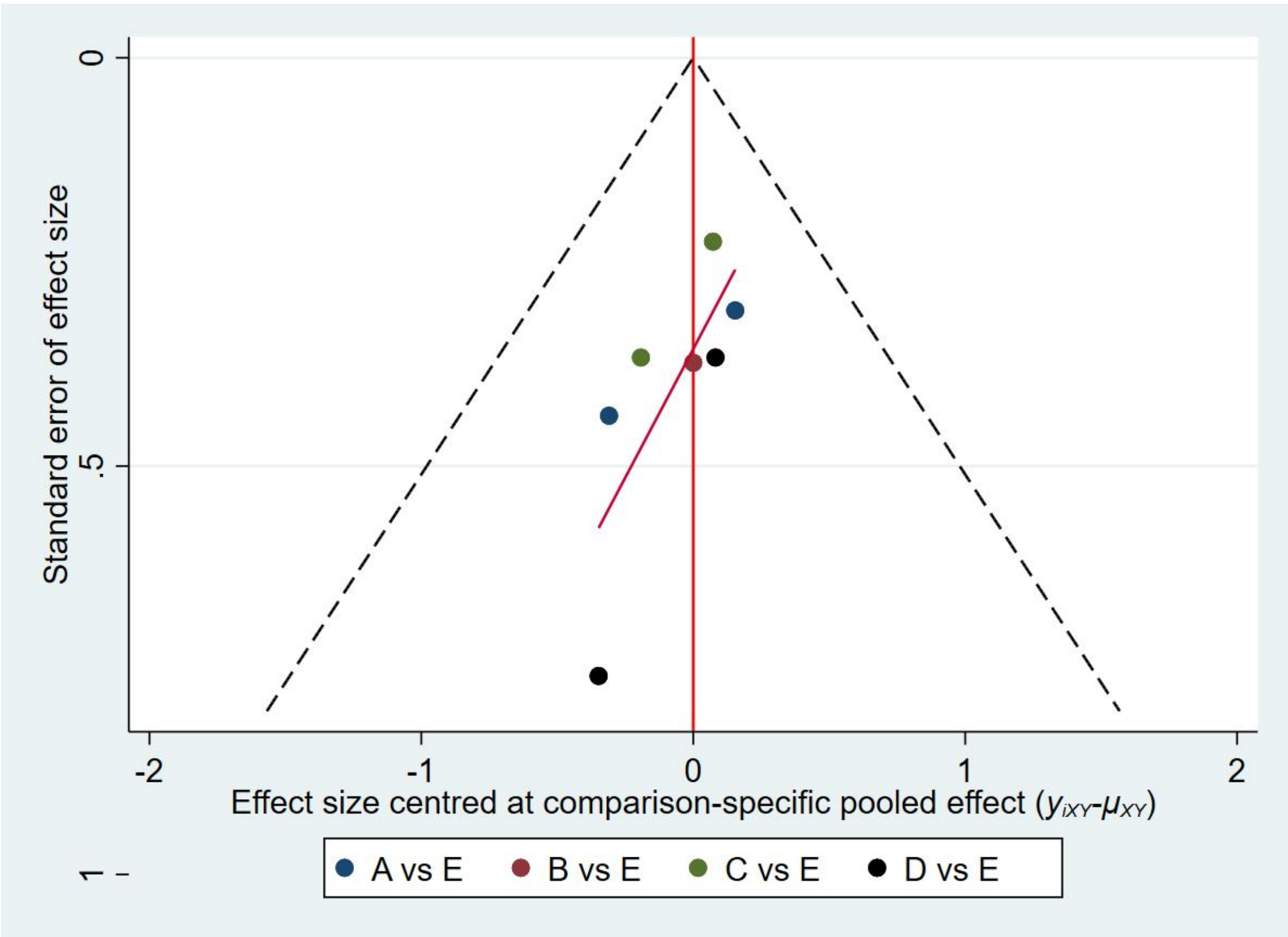

Figure S9.7: Funnel plot of **HR max**

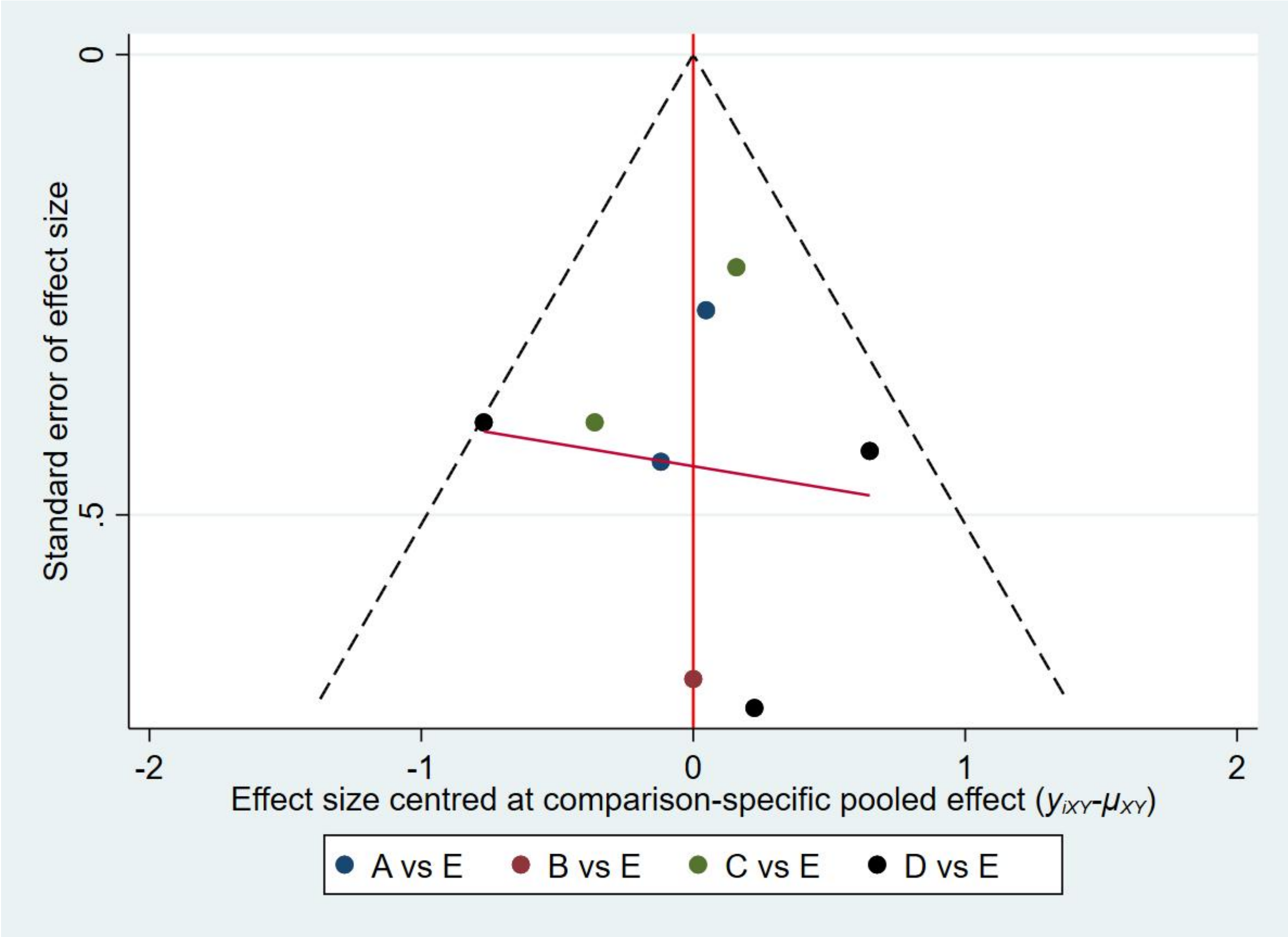

Appendix 10: league table of Summary

Table S10.1: SF-36

AE			
5.59 (-5.74, 15.31)	AE＋BT		
-11.42 (-32.09, 9.48)	-16.95 (-38.23, 5.33)	AE＋RE	
3.29 (-4.47, 10.35)	-2.32 (-11.63, 7.89)	14.65 (-5.28, 34.24)	UC

Table S10.2: 6MWD

AE				
-62.1 (-132.94, 9.78)	BT			
-0.35 (-40.53, 55.94)	62.12 (-2.84, 139.86)	AE+BT		
-29.29 (-119.2, 76.47)	32.54 (-68.61, 149.63)	-29.89 (-121.87, 68.23)	AE+RE	
**39.16 (6.29, 81.68)**	**101.72 (43.65, 166.28)**	39.8 (0.78, 71.2)	69.09 (-25.03, 153.2)	UC

Table S10.3: NT-proBNP

AE			
99.65 (-1318.77, 1444.73)	BT		
-91.71 (-1154.44, 835.03)	-207.51 (-1714.41, 1331.94)	AE+BT	
-64.27 (-664.37, 386.38)	-180.98 (-1449.61, 1106.33)	24.43 (-819.04, 873.75)	UC

Table S10.4: Peak VO_2

AE				
3.2 (-4.3, 10.15)	BT			
0.01 (-3.09, 2.61)	-3.23 (-10.27, 4.18)	AE+BT		
0.2 (-3.97, 3.78)	-3.06 (-10.42, 4.56)	0.2 (-3.77, 4.04)	AE+RE	
**4.16 (1.48, 6.23)**	0.9 (-5.76, 7.94)	**4.15 (1.66, 6.51)**	3.95 (0.88, 7.02)	UC

Table S10.5: FEV_1%

AE			
1.82 (-31.69, 30.73)	BT		
-0.83 (-20.11, 18.52)	-2.6 (-24.91, 24.17)	AE+BT	
4.58 (-22.6, 31.93)	3.07 (-10.5, 20.36)	5.42 (-13.83, 24.53)	UC

Table S10.6: HR rest

AE				
5.38 (-7.58, 20.57)	BT			
1.24 (-9.19, 13.84)	-4.13 (-18.14, 9.89)	AE+BT		
-1.63 (-12.81, 11.3)	-7.05 (-21.51, 7.3)	-2.93 (-14.93, 9)	AE+RE	
2.54 (-4.44, 12.02)	-2.73 (-14.07, 8.66)	1.38 (-6.68, 9.52)	4.27 (-4.47, 13.28)	UC

Table S10.7: HR max

AE				
1.37 (-27.85, 35.6)	BT			
-10.39 (-35.84, 18.69)	-12.04 (-43.76, 19.19)	AE+BT		
-1.15 (-23.72, 28.11)	-2.9 (-31.64, 28.9)	9.24 (-14.82, 36.5)	AE+RE	
7.46 (-9.59, 29.12)	6.02 (-19, 31.16)	18.08 (-0.81, 37.28)	8.78 (-9.72, 24.92)	UC

Appendix 11: SUCRA and cumulative probability plots

Figure S11.1: Cumulative ranking curve plots of exercise types for **SF-36** in range network. Higher surface under the curve reflects higher probability of worse with **SF-36**.

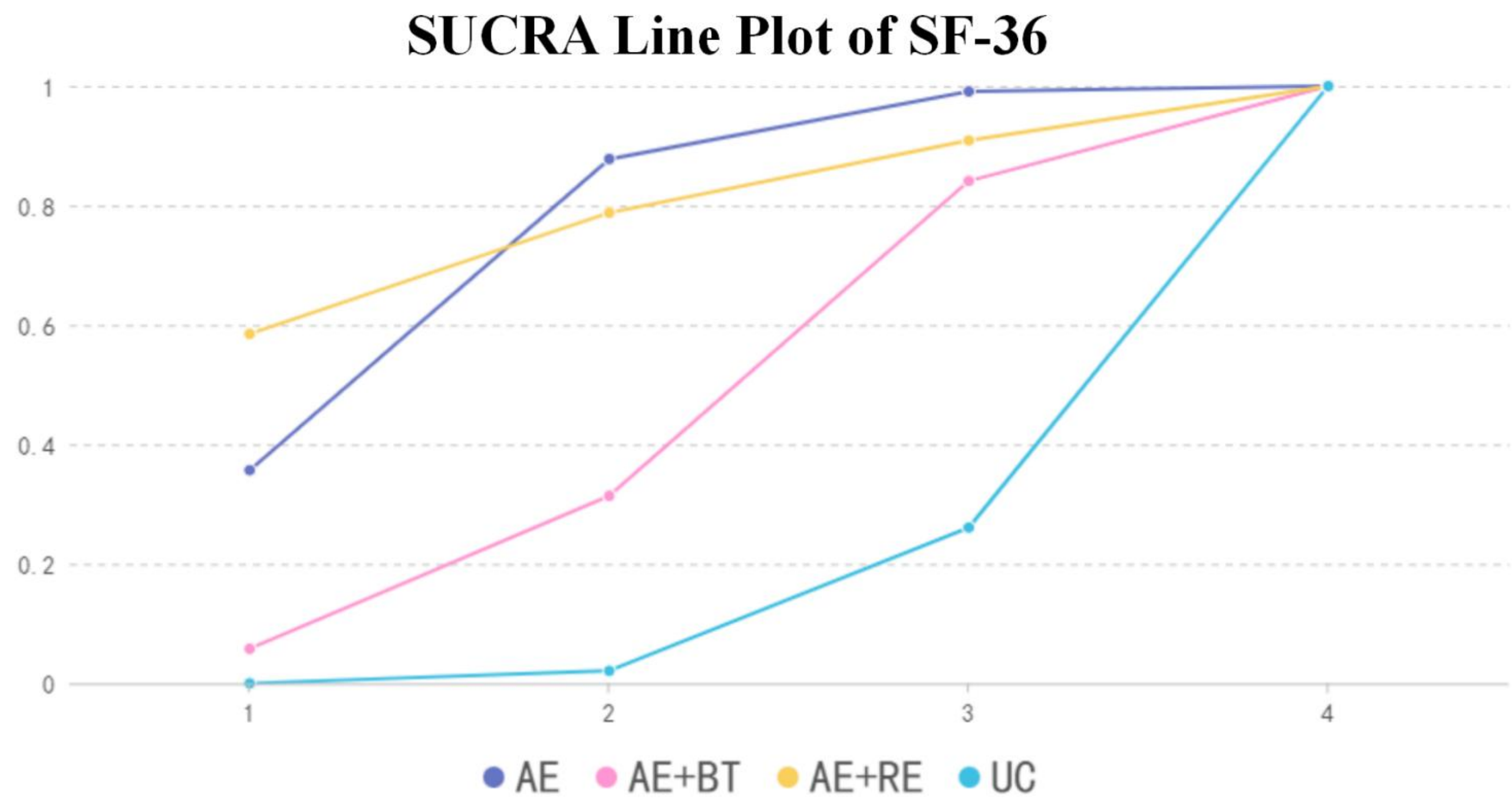

Table S11.1: SUCRA of the effects of various exercise types on **SF-36**

Treatment	SUCRA	PrBest	MeanRank
AE	73.8	35.4	1.8
AE+BT	40.2	5.6	2.8
AE+RE	76.4	58.9	1.7
UC	9.6	0.1	3.7

NOTE: Aerobic Exercise(AE)、Breathing Training(BT)、Aerobic Exercise + Breathing Training(AE+BT)、Combined Aerobic and Resistance Exercise(AE+RE)、Usual Care(UC)

Figure S11.2: Cumulative ranking curve plots of exercise types for **6MWD** in range network. Higher surface under the curve reflects higher probability of worse with **6MWD**.

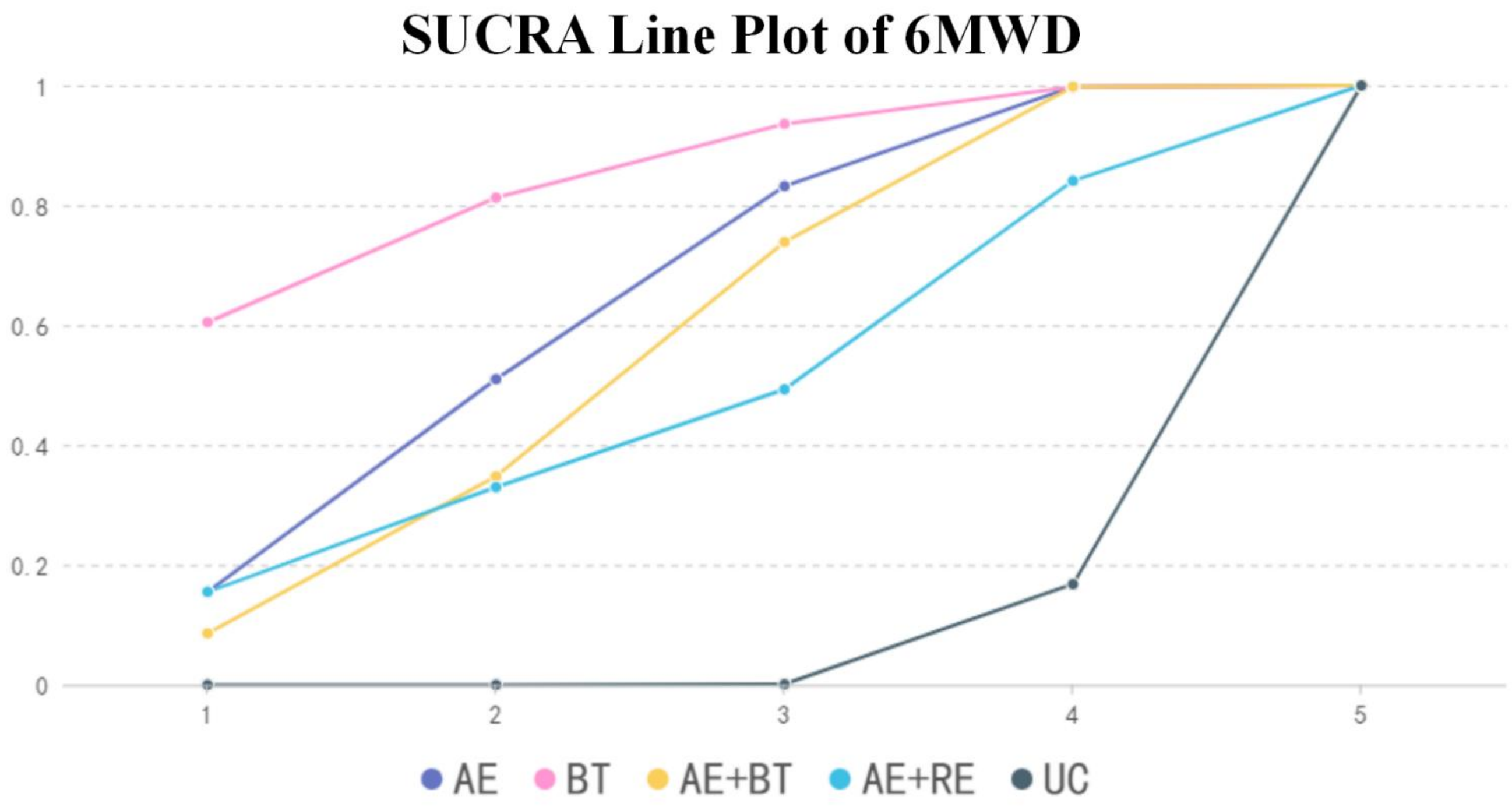

Table S11.2: SUCRA of the effects of various exercise types on **6MWD**

Treatment	SUCRA	PrBest	MeanRank
AE	62.4	15.5	2.5
BT	83.8	60.5	1.6
AE+BT	54.2	8.6	2.8
AE+RE	45.4	15.5	3.2
UC	4.2	0	4.8

NOTE: Aerobic Exercise(AE)、Breathing Training(BT)、Aerobic Exercise + Breathing Training(AE+BT)、Combined Aerobic and Resistance Exercise(AE+RE)、Usual Care(UC)

Figure S11.3: Cumulative ranking curve plots of exercise types for **NT-proBNP** in range network. Higher surface under the curve reflects higher probability of worse with **NT-proBNP**.

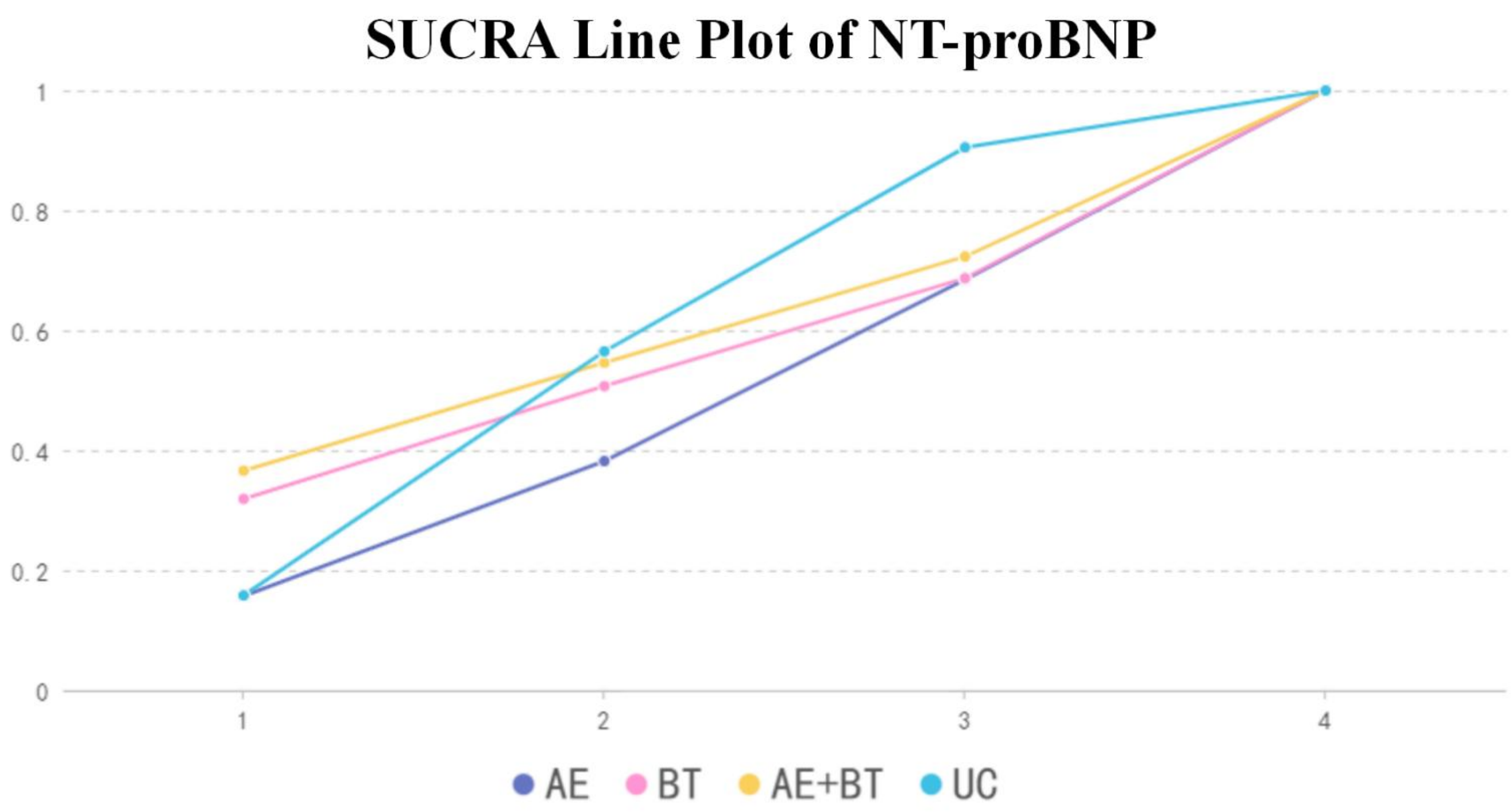

Table S11.3: SUCRA of the effects of various exercise types on **NT-proBNP**

Treatment	SUCRA	PrBest	MeanRank
AE	40.8	15.7	2.8
BT	50.4	31.9	2.5
AE+BT	54.5	36.6	2.4
UC	54.2	15.8	2.4

NOTE: Aerobic Exercise(AE)、Breathing Training(BT)、Aerobic Exercise + Breathing Training(AE+BT)、Combined Aerobic and Resistance Exercise(AE+RE)、Usual Care(UC)

Figure S11.4: Cumulative ranking curve plots of exercise types for **Peak VO_2** in range network. Higher surface under the curve reflects higher probability of worse with **Peak VO_2**.

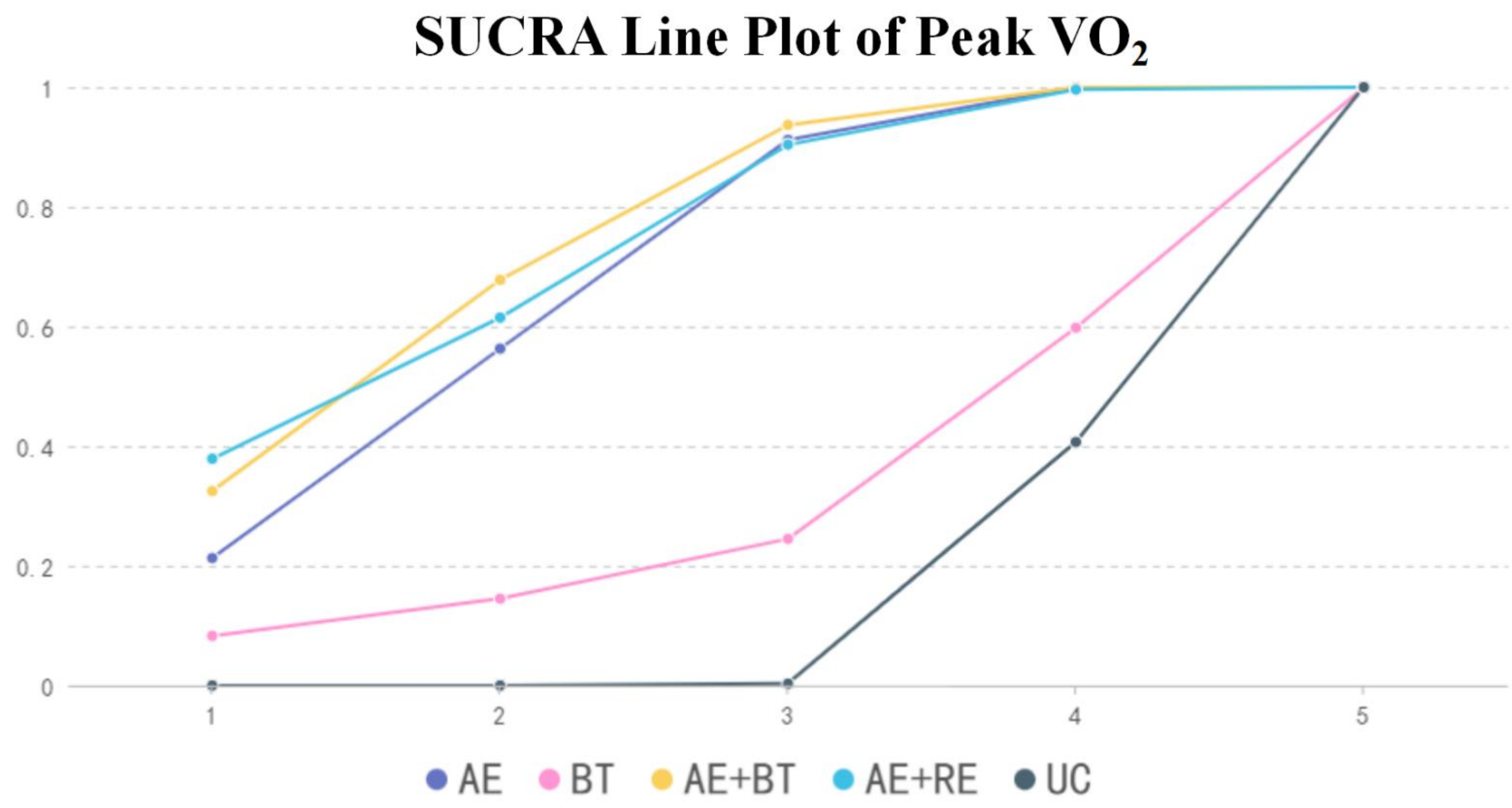

Table S11.4: SUCRA of the effects of various exercise types on **Peak VO_2**

Treatment	SUCRA	PrBest	MeanRank
AE	67.2	21.7	2.3
BT	27.4	8.6	3.9
AE+BT	73.1	31.8	2.1
AE+RE	72.1	37.9	2.1
UC	10.1	0	4.6

NOTE: Aerobic Exercise(AE)、Breathing Training(BT)、Aerobic Exercise + Breathing Training(AE+BT)、Combined Aerobic and Resistance Exercise(AE+RE)、Usual Care(UC)

Figure S11.5: Cumulative ranking curve plots of exercise types for **FEV_1%** in range network. Higher surface under the curve reflects higher probability of worse with **FEV_1%**.

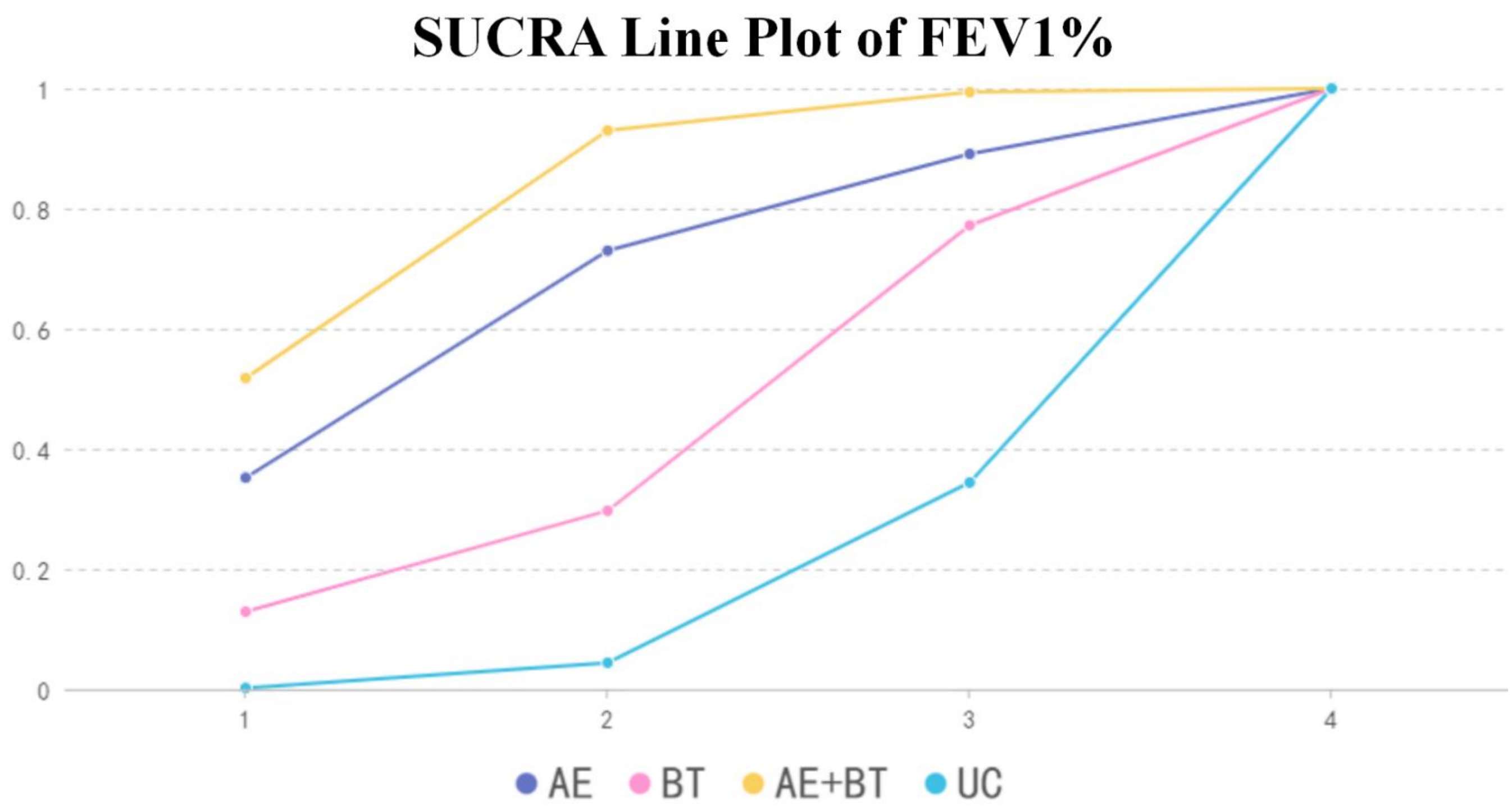

Table S11.5: SUCRA of the effects of various exercise types on **FEV_1%**

Treatment	SUCRA	PrBest	MeanRank
AE	66	36.3	2
BT	40.1	12.9	2.8
AE+BT	80.8	50.5	1.6
UC	13.1	0.3	3.6

NOTE: Aerobic Exercise(AE)、Breathing Training(BT)、Aerobic Exercise + Breathing Training(AE+BT)、Combined Aerobic and Resistance Exercise(AE+RE)、Usual Care(UC)

Figure S11.6: Cumulative ranking curve plots of exercise types for **HR rest** in range network. Higher surface under the curve reflects higher probability of worse with **HR rest**.

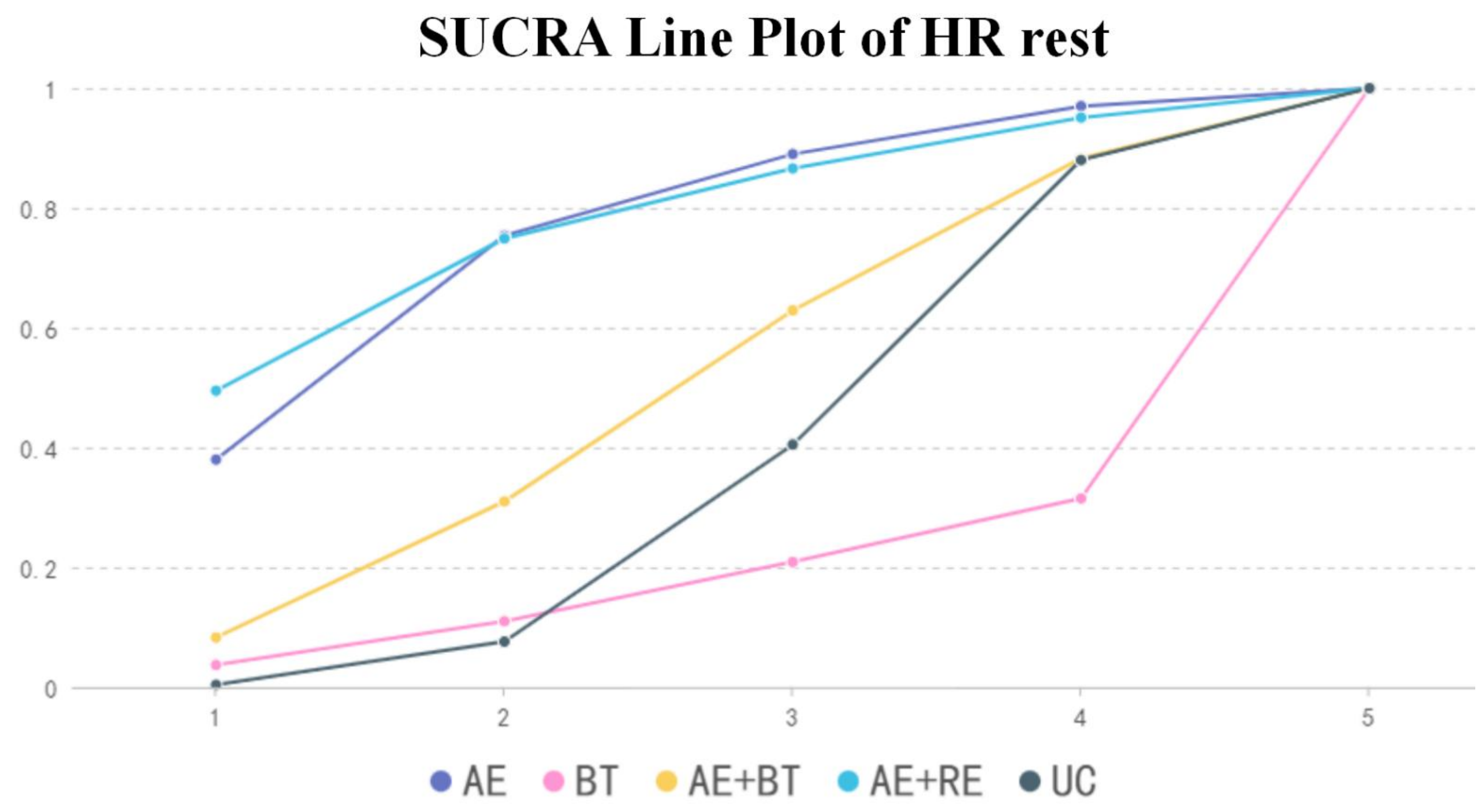

Table S11.6: SUCRA of the effects of various exercise types on **HR rest**

Treatment	SUCRA	PrBest	MeanRank
AE	75.2	39.2	2
BT	16.4	3.6	4.3
AE+BT	48.2	8.4	3.1
AE+RE	76	48.3	2
UC	34.2	0.4	3.6

NOTE: Aerobic Exercise(AE)、Breathing Training(BT)、Aerobic Exercise + Breathing Training(AE+BT)、Combined Aerobic and Resistance Exercise(AE+RE)、Usual Care(UC)

Figure S11.7: Cumulative ranking curve plots of exercise types for **HR max** in range network. Higher surface under the curve reflects higher probability of worse with **HR max**.

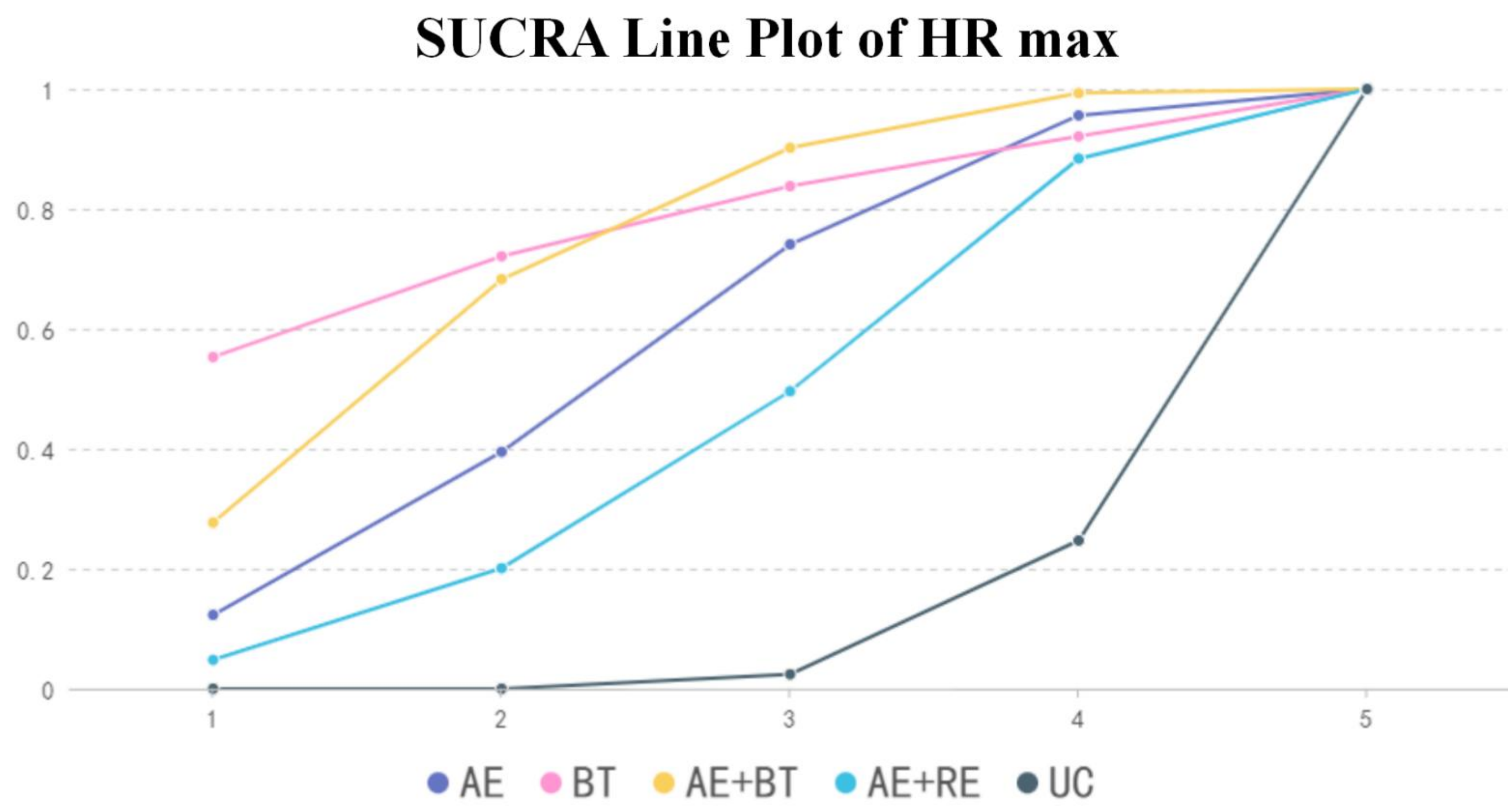

Table S11.7: SUCRA of the effects of various exercise types on **HR max**

Treatment	SUCRA	PrBest	MeanRank
AE	55.3	12.3	2.8
BT	75.8	55.3	2
AE+BT	71.4	27.7	2.1
AE+RE	40.7	4.8	3.4
UC	6.8	0	4.7

NOTE: Aerobic Exercise(AE)、Breathing Training(BT)、Aerobic Exercise + Breathing Training(AE+BT)、Combined Aerobic and Resistance Exercise(AE+RE)、Usual Care(UC)

Appendix 12: The meta-regression of the factors that may lead to differences to the main outcome indicators

Factors	SF-36				
	Coefficient	std err	t	P value	95% CrI
**Exercise duration**	0.0451	0.263	0.171	0.870	-0.481, 0.571
**Exercise frequency**	-0.6123	0.284	-2.156	0.098	-1.399, 0.174
**Age**	-0.0152	0.168	-0.090	0.931	-0.353, 0.322

Factors	Peak VO_2				
	Coefficient	std err	t	P value	95% CrI
**Exercise duration**	0.055	0.295	0.186	0.865	-0.545, 0.655
**Exercise frequency**	-0.472	0.442	-1.068	0.258	-1.471, 0.527
**Age**	-0.018	0.198	-0.091	0.930	-0.408, 0.372

Appendix 13: Certainty of evidence assessment using GRADE/CINeMA

Table S13.1. Summary of certainty-of-evidence assessment for key network comparisons

Outcome	Key comparison	Effect estimate	Main reasons for downgrading	Overall certainty	Interpretation
SF-36	AE+RE vs UC	MD 14.65, 95% CrI -5.28 to 34.24	Imprecision, sparse network, limited direct evidence, clinical indirectness	Low	AE+RE ranked favourably, but the evidence is uncertain and should be interpreted as exploratory.
SF-36	AE+BT vs UC	MD -2.32, 95% CrI -11.63 to 7.89	Imprecision, sparse network, risk of bias, clinical indirectness	Low to very low	The estimate was uncertain; no confirmatory conclusion can be drawn.
6MWD	BT vs UC	MD 101.72 m, 95% CrI 43.65 to 166.28	Sparse network, limited direct evidence, imprecision, potential small-study effects	Low	BT showed a favourable point estimate, but the evidence is exploratory rather than confirmatory.
6MWD	AE+BT vs UC	MD 39.80 m, 95% CrI 0.78 to 71.20	Risk of bias, indirectness, heterogeneity and imprecision	Low	AE+BT may improve 6MWD, but certainty is limited.
6MWD	AE vs UC	MD 39.16 m, 95% CrI 6.29 to 81.68	Risk of bias, heterogeneity and imprecision	Low	AE may improve functional capacity, but the certainty of evidence is limited.
Peak VO_2	AE+BT vs UC	MD 4.15 $mL \cdot kg^{-1} \cdot min^{-1}$, 95% CrI 1.66 to 6.51	Sparse network, imprecision and limited direct active-comparator evidence	Low	AE+BT showed a favourable estimate, but superiority over other active interventions is not confirmed.
Peak VO_2	AE+RE vs UC	MD 3.95 $mL \cdot kg^{-1} \cdot min^{-1}$, 95% CrI 0.88 to 7.02	Sparse network, imprecision and indirectness	Low	AE+RE may be beneficial, but evidence remains uncertain.
NT-proBNP	AE vs UC	MD -64.27 pg/mL, 95% CrI -664.37 to 386.38	Very wide credible intervals, sparse network and imprecision	Very low	Evidence is insufficient to determine effects on NT-proBNP.
NT-proBNP	AE+BT vs UC	MD 24.43 pg/mL, 95% CrI -819.04 to 873.75	Very wide credible intervals, sparse network and	Very low	No reliable conclusion can be drawn.

			imprecision		
FEV_1%	AE+BT vs UC	MD 5.42%, 95% CrI -13.83 to 24.53	Very small network, imprecision and indirectness	Very low	The ranking signal is exploratory and not confirmatory.
Resting HR	AE+RE vs UC	MD 4.27 bpm, 95% CrI -4.47 to 13.28	Sparse network, imprecision and limited direct evidence	Low to very low	Evidence is insufficient for a firm conclusion.
Maximum HR	BT vs UC	MD 6.02 bpm, 95% CrI -19.00 to 31.16	Sparse network, imprecision and limited direct evidence	Low to very low	Evidence is insufficient for a firm conclusion.

Note: AE, aerobic exercise; BT, breathing training; RE, resistance exercise; UC, usual care; 6MWD, 6-minute walk distance; NT-proBNP, N-terminal pro-B-type natriuretic peptide; FEV_1%, forced expiratory volume in 1 second percentage predicted; HR, heart rate; VO_2, oxygen uptake. Certainty was assessed using the GRADE approach adapted for network meta-analysis and the CINeMA framework. The main downgrading domains included within-study bias, reporting bias, indirectness, imprecision, heterogeneity and incoherence. Because many networks were sparse and direct active-versus-active comparisons were limited, most findings should be interpreted as exploratory rather than confirmatory. Effect estimates are network estimates expressed as mean differences with 95% credible intervals. Positive values indicate higher values in the active intervention than in UC, except where clinical interpretation depends on the direction of the outcome. Full league-table estimates are provided in Supplementary Appendix 10.

Appendix 14: Sensitivity analysis by pulmonary hypertension subtype

Appendix 14. Sensitivity analysis by pulmonary hypertension subtype

Outcome	Primary network evidence	PAH-only sensitivity analysis	CTEPH-only analysis	CHD-associated PAH/PH analysis	Interpretation
SF-36	6 studies; included PAH-only and related PH populations	Network became sparse; estimates imprecise	Not feasible; insufficient CTEPH-only trials	Not feasible; insufficient CHD-associated trials	Subtype-specific certainty limited; primary ranking should be interpreted cautiously.
6MWD	13 studies; best-connected network	Feasible where connected; direction broadly consistent with primary analysis	Not feasible as separate NMA; CTEPH evidence limited	Not feasible as separate NMA; CHD-associated evidence limited	PAH-only analysis did not materially change the overall direction, but precision decreased.
NT-proBNP	5 studies	Sparse and imprecise after subtype restriction	Not feasible	Not feasible	No reliable subtype-specific conclusion.
Peak VO_2	9 studies	Feasible where connected; direction broadly consistent with primary analysis	Not feasible as separate NMA	Not feasible as separate NMA	Findings generally supported exercise rehabilitation, but superiority between active nodes remains exploratory.
FEV_1%	4 studies	Very sparse/disconnected after subtype restriction	Not feasible	Not feasible	Evidence insufficient for subtype-specific inference.
Resting HR	7 studies	Sparse and imprecise after subtype restriction	Not feasible	Not feasible	Evidence insufficient for subtype-specific inference.
Maximum HR	8 studies	Sparse and imprecise after subtype restriction	Not feasible	Not feasible	Evidence insufficient for subtype-specific inference.

Note: PAH, pulmonary arterial hypertension; PH, pulmonary hypertension; CTEPH, chronic thromboembolic pulmonary hypertension; CHD, congenital heart disease; NMA, network meta-analysis; 6MWD, 6-minute walk distance; NT-proBNP, N-terminal pro-B-type natriuretic peptide; FEV_1%, forced expiratory volume in 1 second percentage predicted; HR, heart rate; VO_2, oxygen uptake. PAH-only sensitivity analyses were performed only when the network remained connected and data were sufficient. Separate CTEPH-only and CHD-associated PAH/PH network meta-analyses were not feasible because these populations were represented by very few trials and did not form connected outcome-specific networks.

Appendix 15: Adverse events and safety reporting

Study ID	Intervention node	Safety/adverse-event reporting in published report	Exercise-related serious adverse events	Implications for safety assessment
Jia 2024[15]	AE	Adverse reactions were specifically assessed; both groups had no adverse reactions during the intervention.	None reported.	Supports short-term tolerability, but adverse-event definitions and monitoring details were limited.
Jia 2019[16]	AE+BT	Adverse reactions were explicitly observed; no adverse reactions occurred in either group during the intervention.	None reported.	Supports short-term tolerability under supervised/partly supervised respiratory-function training.
Zhang 2025[17]	AE	The study stated that no serious adverse events occurred throughout the intervention period and that tolerance/safety were good.	No serious adverse events reported.	Supports feasibility in stable PAH, but detailed event definitions were limited.
Ehlken 2016[18]	AE+BT	Adverse events were recorded whenever they occurred; oxygen saturation and heart rate were continuously monitored during training. The report stated that the examinations had no complications.	No exercise-related serious adverse event explicitly reported in the available main text.	Safety was actively monitored, but the main text provides limited event-level reporting.
Chan 2013[19]	AE	The report stated that no adverse events were noted in either group.	None reported.	Supports short-term safety of medically supervised treadmill walking in the trial setting.
Mereles 2006[20]	AE+BT	A dedicated Safety section reported that no adverse effects or complications of low-dose physical training occurred, although close supervision was considered necessary.	None reported.	Supports cautious supervised use in clinically stable severe PH; authors emphasized hospital initiation and close monitoring.
González-Saiz 2017[21]	AE+BT	Safety was a secondary endpoint. The report stated that no major adverse event was noted due to exercise; one atrioventricular nodal re-entrant tachycardia episode during post-intervention CPET and one dizziness episode during training attributed to hypoglycaemia were noted.	No major exercise-related adverse event reported.	Supports supervised feasibility, while minor/non-attributable events were observed and should be described.
Yılmaz 2020[22]	AE	The report stated that upper-extremity aerobic exercise and comparator exercises were well tolerated, and that no adverse events or signs of clinical worsening of right heart failure were detected during or after sessions.	None reported.	Supports short-term tolerability of upper-extremity aerobic exercise in stable PAH.
Hemnes 2021[23]	AE	The study was monitored by an independent Data and Safety Monitoring Board. It reported one unrelated serious adverse event before randomization and no serious adverse events attributable to the intervention; incident adverse events common in PAH were similar between groups.	No intervention-attributable serious adverse events reported.	Supports feasibility of the mobile health physical-activity intervention; safety events were not primarily exercise-session adverse events.

Martinez-Quintana 2010[24]	AE+RE	Two participants initially had severe cyanosis and poor exercise tolerance requiring protocol changes, but no adverse events such as symptom progression or heart failure were observed.	None reported.	Supports carefully adapted rehabilitation in CHD-associated PH, but sample size was extremely small.
Saglam 2015[25]	BT	The report stated that inspiratory muscle training was well tolerated by all patients; no syncope/presyncope episodes, arrhythmias, or respiratory complications occurred. One patient had muscle soreness during the first session.	None reported.	Supports short-term tolerability of IMT; minor muscle soreness was reported.
Kagioglou 2021[26]	AE+RE	The abstract/report stated that no exercise-induced complications were observed throughout the study.	None reported.	Supports supervised long-term exercise feasibility, but event definitions were not detailed in the abstract-level statement.
Rakhmawati 2020[27]	AE	Adverse events were recorded if they occurred. All subjects tolerated the exercise programme, with no adverse events such as chest pain, cold sweats, shortness of breath, leg cramps, dizziness, intolerable fatigue, or symptomatic oxygen desaturation ≥10%. One control-group subject died from causes unrelated to exercise.	No exercise-related serious adverse events reported.	Supports hospital-plus-home exercise feasibility in ASD-associated PAH; one unrelated death occurred in the control group.
Karapolat 2019[28]	AE+BT	No adverse events attributed to cardiopulmonary rehabilitation, including syncope, arrhythmia, severe hypotension, or haemoptysis, were reported; no treatment modification was performed.	None reported.	Supports short-term safety of the rehabilitation protocols in the trial setting.
Tran 2021[29]	BT	The main text available for extraction did not provide a dedicated adverse-event summary or report exercise-related serious adverse events.	Not clearly reported.	Insufficient safety detail for this review; absence of a reported event should not be interpreted as systematic safety confirmation.
Babu 2019[30]	AE+BT	The report stated that no adverse events or fatalities were observed during the study; non-exercise-related adverse events occurred in both groups, including breathlessness, vertigo, haemoptysis, lower respiratory infection and warfarin-induced bleeding. One control participant with warfarin-induced bleeding died; authors stated that two fatalities were not exercise-related.	No exercise-related serious adverse events reported; non-exercise-related events and fatalities occurred.	Supports cautious interpretation: no exercise-related harm signal, but unrelated clinical events occurred and supervision was emphasized.

Abbreviations: AE, aerobic exercise; BT, breathing training; RE, resistance exercise; UC, usual care; PAH, pulmonary arterial hypertension; PH, pulmonary hypertension; CTEPH, chronic thromboembolic pulmonary hypertension; CHD, congenital heart disease; IMT, inspiratory muscle training; CPET, cardiopulmonary exercise testing; CPR, cardiopulmonary rehabilitation; ASD, atrial septal defect.

Overall safety conclusion: No clear signal of serious exercise-related harm was identified among studies that explicitly reported safety information. However, adverse-event definitions, monitoring procedures and reporting completeness were heterogeneous, and several trials provided limited safety detail. Therefore, the safety of rehabilitation in PAH and related PH populations should be interpreted in the context of clinical stability, individualized risk assessment and professional supervision.

Appendix 16: Data type used for each study and outcome

Because mixing change-from-baseline values and post-intervention endpoint values within the same outcome network may introduce bias when baseline values differ across studies, we explicitly reported the data type used for each study-outcome combination. These classifications were also considered when assessing transitivity, indirectness and certainty of evidence.

Table S16.1. Data type used in the quantitative synthesis for each included study and outcome

The table specifies whether the network meta-analysis used change-from-baseline values or post-intervention endpoint values for each study-outcome combination. When both types were available, change-from-baseline values were prioritised. When change data were not available, post-intervention endpoint values were used if the measurement scale was comparable. Study-outcome combinations that did not contribute data to a specific network are marked as NI.

Study ID	Population	Node	SF-36	6MWD	NT-proBNP	Peak VO_2	FEV_1%	Resting HR	Maximum HR	Notes
Jia 2024[15]	PAH-only	AE	EP	EP	EP	NI	NI	NI	NI	Endpoint data extracted for reported outcomes.
Jia 2019[16]	PAH-only	AE+BT	NI	DER-EP	NI	NI	DER-EP	NI	NI	Median/IQR-like endpoint values converted when required.
Zhang 2025[17]	PAH-only	AE	NI	NI	NI	EP	NI	NI	EP	Endpoint data extracted from the published report/supplement.
Ehlken 2016[18]	PAH/CTE PH mixed	AE+BT	NI	CFB	CFB	CFB	NI	CFB	CFB	Change-from-baseline data were used where reported.
Chan 2013[19]	PAH-only	AE	NI	DER-EP	NI	DER-EP	NI	NI	NI	Mean(SD) endpoint values extracted/converted from report.

Mereles 2006[20]	PAH/CTEPH mixed	AE+BT	CFB	CFB	CFB	CFB	NI	CFB	CFB	Change-from-baseline data were used where reported.
González-Saiz 2017[21]	PAH/CTEPH mixed	AE+BT	NI	EP	EP	EP	NI	EP	EP	Post-intervention endpoint values were used.
Yılmaz 2020[22]	PAH-only	AE	EP	EP	NI	NI	NI	EP	EP	Post-intervention endpoint values were used.
Hemnes 2021[23]	PAH-only	AE	NI	EP	NI	NI	NI	EP	EP	Post-intervention endpoint values were used.
Martinez-Quintana 2010[24]	CHD-associated PAH/PH	AE+RE	NI	EP	NI	NI	EP	EP	NIv	Post-intervention endpoint values were used.
Saglam 2015[25]	PAH-only	BT	EP	EP	NI	EP	NI	NI		Post-intervention endpoint values were used.
Kagioglou 2021[26]	PAH/CTEPH mixed	AE+RE	NI	DER-EP	NI	DER-EP	NI	DER-EP	NI	Mean(SD) endpoint values extracted/converted from report.
Rakhmawati 2020[27]	CHD-associated PAH/PH	AE	NI	NI	EP	NI	NI	NI	EP	Post-intervention endpoint values were used.
Karapolat 2019[28]	PAH-only	AE+BT	EP	EP	NI	EP	EP	NI	NI	Post-intervention endpoint values were used.
Tran 2021[29]	CTEPH-only/mixed PH	BT	NI	NI	NI	EP	EP	NI	EP	Post-intervention endpoint values were used.
Babu 2019[30]	PAH/PH population	AE+BT	CFB	CFB	NI	CFB	NI	NI	NI	Change-from-baseline data were used where reported.

Note: Abbreviations and codes: AE, aerobic exercise; RE, resistance exercise; BT, breathing training; AE+RE, aerobic exercise plus resistance exercise; AE+BT, aerobic exercise plus breathing training; UC, usual care; PAH, pulmonary arterial hypertension; PH, pulmonary hypertension; CTEPH, chronic thromboembolic pulmonary hypertension; CHD, congenital heart disease; SF-36, Short Form-36; 6MWD, 6-minute walk distance; NT-proBNP, N-terminal pro-B-type natriuretic peptide; VO_2, oxygen uptake; FEV1%, forced expiratory volume in 1 second percentage predicted; HR, heart rate.
CFB, change-from-baseline values; EP, post-intervention endpoint values; DER-CFB, derived or converted change-from-baseline values; DER-EP, derived or converted post-intervention endpoint values; NI, not included in the corresponding outcome network or not available for quantitative synthesis.
Where standard deviations were not directly reported, they were derived from available summary statistics, such as standard errors, confidence intervals, p values, or other reported dispersion measures, following Cochrane Handbook guidance. Where median and interquartile range or graphical data were reported, values were converted or digitally extracted when appropriate and checked by a second reviewer.

Appendix 17: Intervention node classification rationale

Intervention nodes were assigned according to the dominant planned exercise components rather than programme setting, supervision, frequency, session duration, programme duration, intensity or adjunctive components such as stretching, flexibility work or chest-expansion exercises. This approach was used to maintain clinically interpretable nodes for network meta-analysis while acknowledging residual clinical heterogeneity. Because several rehabilitation programmes were multicomponent and did not map perfectly onto a single conventional exercise category, node assignment should be interpreted as a pragmatic classification for evidence synthesis rather than as confirmation that all interventions within a node were identical in content, dose or delivery.

Study ID	Assigned node	Comparator	Main intervention components	Rationale for node classification	Potential limitation
Jia 2024[15]	AE	UC	Wuqinxi bird-play exercise	Classified as AE because the programme was a structured, low-intensity, rhythmic whole-body exercise intervention intended to improve functional capacity.	Traditional Chinese exercise is not identical to conventional treadmill, walking or cycle-based aerobic training; interpretation should therefore be pragmatic.
Jia 2019[16]	AE+BT	UC	Breathing exercises plus whole-body breathing exercises	Classified as AE+BT because the programme combined structured breathing exercises with coordinated whole-body breathing-related movement.	This classification reflects dominant breathing-related and whole-body movement components rather than equivalence to standard aerobic training plus inspiratory muscle training.
Zhang 2025[17]	AE	UC	Cycle ergometer training	Classified as AE because cycling was the dominant aerobic component.	Conventional supervised aerobic training.
Ehlken 2016[18]	AE+BT	UC	Exercise training plus respiratory training	Classified as AE+BT because aerobic/physical exercise was deliberately combined with respiratory training.	Delivered as rehabilitation clinic training plus home continuation.
Chan 2013[19]	AE	UC	Treadmill walking	Classified as AE because treadmill walking was the dominant planned exercise component.	Conventional outpatient supervised AE.
Mereles 2006[20]	AE+BT	UC	Exercise training plus respiratory training	Classified as AE+BT because the programme deliberately combined physical/aerobic exercise with respiratory training.	Multicomponent inpatient-plus-home programme.
González-Saiz 2017[21]	AE+BT	UC	Aerobic exercise plus inspiratory muscle training	Classified as AE+BT because both aerobic and inspiratory muscle training components were planned.	Hospital-based combined training.
Yılmaz 2020[22]	AE	UC	Upper-extremity aerobic exercise	Classified as AE because the dominant component was aerobic upper-limb	Aerobic modality differs from lower-limb treadmill/cycle training.

				exercise.	
Hemnes 2021[23]	AE	UC	Walking-based physical activity intervention	Classified as AE because the intervention primarily targeted aerobic physical activity through walking.	Remotely supported/home-based delivery may differ from supervised hospital programmes.
Martinez-Quintana 2010[24]	AE+RE	UC	Interval aerobic training plus resistance exercises	Classified as AE+RE because both aerobic and resistance components were planned.	Small sample size and congenital heart disease-associated PAH/PH population.
Saglam 2015[25]	BT	UC	Inspiratory muscle training	Classified as BT because inspiratory muscle training was the dominant intervention component.	BT effects may be specific to respiratory muscle outcomes and may not generalise to broader rehabilitation.
Kagioglou 2021[26]	AE+RE	UC	Aerobic exercise plus resistance exercise, with stretching and breathing/chest-expansion exercises	Classified as AE+RE because aerobic and resistance exercise were the dominant planned components.	The programme also included stretching and breathing/chest-expansion exercises; effects may partly reflect co-interventions, supervision and delivery setting.
Rakhmawati 2020[27]	AE	UC	Walking/cycle-based exercise programme	Classified as AE because the dominant component was aerobic walking/cycling.	Partly supervised hospital-plus-home delivery.
Karapolat 2019[28]	AE+BT	AE	Flexibility, aerobic exercise and breathing exercises	Classified as AE+BT because the active intervention combined aerobic exercise with breathing exercises; comparator was AE.	This was the only active-comparator trial and may strongly influence indirect active comparisons.
Tran 2021[29]	BT	UC	Inspiratory muscle training	Classified as BT because inspiratory muscle training was the dominant intervention component.	CTEPH-only population; considered in subtype sensitivity interpretation.
Babu 2019[30]	AE+BT	UC	Home-based exercise training with aerobic and breathing components	Classified as AE+BT because aerobic and breathing components were both planned.	Home-based delivery may differ from supervised hospital programmes.

Note: Intervention nodes were used as pragmatic categories for evidence synthesis. Differences in exercise frequency, session duration, programme duration, intensity, setting, supervision, progression, adherence support and co-interventions may have influenced treatment effects independently of exercise modality. Therefore, treatment-node comparisons should be interpreted as comparisons of broad rehabilitation strategies rather than as definitive evidence for a specific exercise prescription.

Appendix 18. Protocol modifications and timing

Table S18.1. Protocol modifications made after PROSPERO registration
Dates are reported as implementation or finalisation dates. Search-related refinements are reported as having been implemented by the final updated search date, whereas revision-stage refinements are reported using the date on which they were finalised or submitted during manuscript revision.

Protocol item	Original PROSPERO record	Modification in the completed review	Date of modification	Timing relative to the review process	Rationale and impact
Database coverage	The original PROSPERO record listed CINAHL, Embase.com, MEDLINE and PubMed as the main bibliographic databases.	The completed review searched PubMed, Embase, Web of Science, the Cochrane Library, CNKI, VIP and Wanfang.	Implemented by 2 June 2026	Implemented by the final updated search. The modification was completed before final screening of the updated records, before final data extraction and before quantitative synthesis.	Database coverage was broadened to improve search comprehensiveness and to capture eligible trials published in both international and Chinese databases. This refinement did not change the review question.
Language eligibility	The original PROSPERO record stated that only studies published in English would be included.	Studies published in English or Chinese were eligible in the completed review.	Implemented by 2 June 2026	Implemented by the final updated search. The modification was completed before final screening of the updated records, before final data extraction and before quantitative synthesis.	The language criterion was expanded because relevant exercise-rehabilitation trials in PAH and related pulmonary hypertension populations were available in Chinese-language journals. This was intended to reduce language bias and improve completeness.

Protocol item	Original PROSPERO record	Modification in the completed review	Date of modification	Timing relative to the review process	Rationale and impact
Final search date	The original PROSPERO record specified studies published before 18 July 2025, with no restriction on the search start date.	The final updated search covered records from database inception to 2 June 2026.	2 June 2026	Implemented before final screening of the updated records, before final data extraction and before quantitative synthesis.	The search was updated to capture newly published eligible studies before completion of the final analysis.
Trial registry searches	The original record stated that only published studies would be sought; other identification methods included contacting authors or experts and reference list checking.	ClinicalTrials.gov, the WHO International Clinical Trials Registry Platform and the Chinese Clinical Trial Registry were also searched.	Implemented by 2 June 2026	Implemented before final interpretation and before manuscript revision. Registry searches were used to support assessment of unpublished, ongoing or terminated studies and reporting bias.	Trial registry searches were added to improve transparency and to identify ongoing, unpublished or terminated studies. This refinement did not change eligibility criteria or the review objective.
Risk-of-bias assessment tool	The original PROSPERO record planned use of the Cochrane RoB-1 tool.	Risk of bias was assessed using the Cochrane Risk of Bias 2 tool for randomised trials.	Finalised on 21 June 2026	Finalised during manuscript revision after initial study selection and data extraction, and before final certainty assessment, final interpretation and revised manuscript submission.	RoB 2 was used because it is the current Cochrane tool for assessing risk of bias in randomised trials and allows outcome-level assessment. This refinement improved methodological rigour and did not alter the included studies or review objective.

Protocol item	Original PROSPERO record	Modification in the completed review	Date of modification	Timing relative to the review process	Rationale and impact
Certainty-of-evidence assessment	The original PROSPERO record stated that the certainty of evidence would be assessed using the GRADE approach.	Certainty of evidence was assessed using the GRADE approach adapted for network meta-analysis and the Confidence in Network Meta-Analysis (CINeMA) framework.	Finalised on 21 June 2026	Added during manuscript revision after primary network estimates were available and before the final Discussion and Conclusion were finalised.	This refinement improved transparency when interpreting sparse networks, imprecision, indirectness, heterogeneity and incoherence. It did not change the primary analysis or core review question.
Pulmonary hypertension subtype sensitivity analysis	Subtype-specific sensitivity analyses were not specified in detail in the original PROSPERO record.	PAH-only sensitivity analyses were performed where the network remained connected. Evidence from CTEPH-only and congenital heart disease-associated PAH/PH populations was summarised narratively because separate connected networks were not feasible.	Added on 21 June 2026	Added during manuscript revision after the primary analysis as a sensitivity analysis, before final interpretation and revised manuscript submission.	This analysis was added to address clinical heterogeneity, assess applicability across pulmonary hypertension subtypes and improve interpretation of transitivity. It did not change the primary objective.

Protocol item	Original PROSPERO record	Modification in the completed review	Date of modification	Timing relative to the review process	Rationale and impact
PROSPERO record update	The original record indicated that pilot work and formal searching/study identification had been initiated, while screening, data extraction, risk-of-bias assessment and data synthesis were not marked as completed.	The PROSPERO record update was submitted to reflect completion of screening, data extraction, risk-of-bias assessment and data synthesis, and to document methodological refinements made during the review process.	Submitted on 21 June 2026	Updated after completion of the review and during manuscript revision.	The update was made to align the registry record with the completed review and to improve transparency regarding review progress and protocol refinements.

Note: PAH, pulmonary arterial hypertension; PH, pulmonary hypertension; CTEPH, chronic thromboembolic pulmonary hypertension; CNKI, China National Knowledge Infrastructure; VIP, Chinese Science and Technology Periodical Database; RoB 2, Cochrane Risk of Bias 2 tool; GRADE, Grading of Recommendations Assessment, Development and Evaluation; CINeMA, Confidence in Network Meta-Analysis. The listed refinements were made to improve completeness, transparency and interpretation of the review. None of these modifications changed the core review question or objective.

Appendix 19. SF-36 scoring and data harmonisation

Table S19.1. SF-36 data used in the quantitative synthesis

This table documents how SF-36 data were handled for the quantitative synthesis. Only directly reported overall, total or composite SF-36 values that were considered comparable on a 0-100 metric were included in the SF-36 network. The review authors did not calculate an overall SF-36 score by averaging or summing the eight individual SF-36 domains, and no review-author formula was applied to combine domain scores. If a study reported only individual domains, physical or mental component summaries, or a non-comparable SF-36 scoring format, those data were not pooled with the overall/composite SF-36 network.

Study ID	SF-36 data reported in original study	Data used in SF-36 network	Scale used for pooling	Direction of scale	Overall score calculated by review authors?	Included in SF-36 NMA	Reason / clarification
Jia 2024[15]	SF-36 quality-of-life score reported by the trial authors.	Directly reported SF-36 value as extracted from the original report.	Comparable 0-100 metric	Higher score indicates better health-related quality of life	No	Yes	No individual SF-36 domains were combined by the review authors.
Mereles 2006[20]	SF-36 overall/composite quality-of-life score reported by the trial authors.mixed	Directly reported SF-36 overall/composite value as extracted from the original report.	Comparable 0-100 metric	Higher score indicates better health-related quality of life	No	Yes	Used because the reported SF-36 value was considered comparable with other overall/composite SF-36 values.
Yılmaz 2020[22]	SF-36 total or overall quality-of-life result reported by the trial authors.	Directly reported SF-36 value as extracted from the original report.	Comparable 0-100 metric	Higher score indicates better health-related quality of life	No	Yes	No transformation to SMD was applied because the pooled values were treated as comparable mean differences.
Saglam 2015[25]	SF-36 quality-of-life result reported by the trial authors.	Directly reported SF-36 value as extracted from the original report.	Comparable 0-100 metric	Higher score indicates better health-related quality of life	No	Yes	Individual domains were not combined by the review authors.
Karapolat 2019[28]	SF-36 quality-of-life score reported by the trial authors.	Directly reported SF-36 value as extracted from the original report.	Comparable 0-100 metric	Higher score indicates better health-related quality of life	No	Yes	Active-comparator study; SF-36 value was used as reported without domain aggregation.

Babu 2019[30]	SF-36 quality-of-life result reported by the trial authors.	Directly reported SF-36 value as extracted from the original report.	Comparable 0-100 metric	Higher score indicates better health-related quality of life	No	Yes	Used in the SF-36 network as a directly reported quality-of-life value.

Note: SF-36, Short Form-36; NMA, network meta-analysis; SMD, standardised mean difference. Mean differences were used for the SF-36 network because the values included in the analysis were treated as directly reported overall, total or composite SF-36 values on a comparable 0-100 metric. The review authors did not calculate an overall SF-36 score from the eight individual domains. Studies reporting only individual domains, physical component summary scores, mental component summary scores, or non-comparable scoring formats should not be pooled with these overall/composite SF-36 values. Accordingly, SMDs were not used for the SF-36 network.

Appendix 20. Transitivity assessment

Table S20.1. Distribution of potential effect modifiers across direct treatment comparisons

Transitivity was assessed by comparing prespecified potential effect modifiers across direct treatment comparisons. The comparison focused on pulmonary hypertension subtype, age, sex distribution, baseline functional status, baseline 6MWD, programme duration, training frequency, setting and supervision. Because several direct comparisons were sparse, the assessment was qualitative and was used to inform judgements about indirectness and certainty of evidence rather than to support formal statistical testing.

Direct comparison	Contributing studies	No. of studies	PH subtype distribution	Age distribution	Sex distribution	Baseline functional status	Baseline 6MWD	Programme duration	Training frequency / dose	Setting / supervision	Transitivity judgement
AE vs UC	Jia 2024; Zhang 2025; Chan 2013; Yilmaz 2020; Hemnes 2021; Rakhmawati 2020	6	Predominantly PAH-only. One study enrolled congenital heart disease-associated PAH/PH.	Reported mean or group ages were generally adult to middle-aged; approximately 35–56 years in studies with available data. Jia 2024 did not clearly report age in the extracted table.	Female-predominant in most studies with reported data; Jia 2024 did not clearly report sex distribution in the extracted table.	WHO FC I–IV across studies, mostly I–III where reported; one study did not clearly report class.	Reported in several trials but not consistently extractable in all reports. Baseline functional capacity was considered when judging comparability.	6 weeks to 3 months.	Varied from 3 sessions/week to daily or remotely supported activity targets.	Hospital/outpatient supervised training, home-based training and mobile/remote support were all represented.	No single effect modifier showed an extreme imbalance that precluded synthesis. However, subtype and delivery heterogeneity, especially the CHD-associated PAH/PH study and remote/home-based delivery, were considered when interpreting indirectness.
AE+BT vs UC	Jia 2019; Ehlken 2016; Mereles 2006; Gonzalez-Saiz 2017; Babu	5	Included PAH-only and mixed PAH/CTEPH cohorts.	Reported mean or median ages were generally middle-aged	Both sexes were represented; sex distribution varied	WHO FC I–IV or II–IV where reported; some	Reported in several trials but not uniformly available	8 weeks to approximately 15 weeks/3.75 months; most	Substantial variation, from 3 sessions/week to 5–7 sessions/w	Supervised inpatient/hospital-based programmes, supervised-to-home continuation	Clinical heterogeneity was present because PAH-only and mixed PAH/CTEPH

	2019			; approximately 45–65 years in studies with available data.	across studies.	studies did not clearly report functional class in the extracted table.	in a harmonised format. Baseline 6MWD was considered alongside population subtype and disease severity.	programmes lasted about 2–4 months.	eek or daily breathing/home components.	and home-based components were represented.	cohorts and different supervision models were combined. The comparison was retained because interventions shared aerobic and breathing components, but certainty and indirectness were interpreted cautiously.
BT vs UC	Saglam 2015; Tran 2021	2	One PAH-only study and one CTEPH-only study.	Reported group mean ages ranged approximately from 47 to 66 years.	Predominantly female in the available reports.	WHO FC II–III in both studies.	Baseline 6MWD was reported or derivable in the original studies, but the direct comparison was sparse.	6 to 8 weeks.	High-frequency inspiratory muscle training, generally daily or 5 days/week.	Hospital/home or supervised training as reported..	Sparse evidence and subtype heterogeneity were important concerns. This comparison was retained for the main network but interpreted cautiously; PAH-only sensitivity analysis excluded the CTEPH-only study.
AE+RE vs UC	Martinez-Quintana 2010; Kagioglou 2021	2	No strictly PAH-only direct evidence. One congenital heart disease-associated PAH/PH cohort and	Reported mean ages varied widely, approximately from the early 20s to mid-50s.	Both sexes represented; group-specific sex reporting was limited in one study.	WHO FC I–IV across the two studies.	Baseline 6MWD or functional performance measures were reported, but direct	Approximately 3 to 6 months, depending on trial protocol and assessment point.	Generally 2–3 sessions/week, with additional programme-specific components.	Home-based or supervised hospital-based settings were represented.	Important transitivity concerns were present because evidence came from non-PAH-only or mixed populations and different delivery models. AE+RE rankings

			one mixed PAH/CTEPH cohort.				evidence was very sparse.				were therefore treated as exploratory and not confirmatory.
AE+BT vs AE	Karapolat 2019	1	PAH-only.	Adult participants; reported age range approximately 20–74 years.	Both sexes represented.	Functional class not clearly reported in the extracted table.	Baseline exercise capacity was assessed in the original trial, but this was the only active-comparator study.	8 weeks.	3 sessions/week, approximately 45–60 min/session.	Hospital-based cardiopulmonary rehabilitation compared with a home-based exercise programme.	This was the only direct active-comparator comparison. It provided limited direct evidence for active-versus-active comparisons and could not by itself establish transitivity across active intervention nodes.

Abbreviations: AE, aerobic exercise; BT, breathing training; RE, resistance exercise; UC, usual care; PAH, pulmonary arterial hypertension; PH, pulmonary hypertension; CTEPH, chronic thromboembolic pulmonary hypertension; CHD, congenital heart disease; WHO FC, World Health Organization functional class; 6MWD, 6-minute walk distance; NR, not reported.

The table summarises potential effect modifiers at the level of direct treatment comparisons. It is intended to support a qualitative assessment of transitivity. Because several direct comparisons contained few studies, the presence of clinical heterogeneity was considered in the certainty-of-evidence assessment and in the interpretation of SUCRA rankings.

Baseline 6MWD and other baseline functional measures were considered where reported. When a study did not report a given effect modifier in a harmonised or extractable format, this was treated as missing information rather than evidence of comparability.

Appendix 21. Network geometry and direct evidence structure

Table S21.1. Distribution of direct evidence across treatment comparisons by outcome network

The table summarises the edge-level direct evidence structure shown in Figure 2. Numbers in parentheses after intervention nodes are participant counts shown next to nodes. Numbers in the direct-comparison column are edge labels indicating the number of studies providing direct evidence.

Outcome network	Nodes represented in Figure 2	Direct comparisons, n studies	Direct active-versus-active evidence	Indirect active-versus-active comparisons	Interpretation
SF-36	AE+BT (n=74); AE (n=62); AE+RE (n=12); UC (n=124)	AE+BT vs UC: 2<br>AE vs UC: 2<br>AE+RE vs UC: 1<br>AE+BT vs AE: 1	AE+BT vs AE: 1 study	AE+RE vs AE; AE+RE vs AE+BT; other active comparisons not directly represented	Sparse UC-centred network with one active-comparator edge; most active comparisons remain indirect or not estimable.
6MWD	AE+BT (n=150); AE (n=83); BT (n=14); AE+RE (n=16); UC (n=249)	AE+BT vs UC: 5<br>AE vs UC: 4<br>AE+RE vs UC: 2<br>BT vs UC: 1<br>AE+BT vs AE: 1	AE+BT vs AE: 1 study	BT vs AE; BT vs AE+BT; BT vs AE+RE; AE+RE vs AE; AE+RE vs AE+BT	Best-connected outcome network, but direct evidence is concentrated around UC; most active-versus-active estimates are mainly indirect.
NT-proBNP	AE+BT (n=73); AE (n=61); UC (n=120)	AE+BT vs UC: 2<br>AE vs UC: 3	None	AE+BT vs AE	Sparse star-shaped network centred on UC; no direct active-versus-active evidence.
Peak VO2	AE+BT (n=65); BT (n=19); AE (n=59); AE+RE (n=12); UC (n=138)	AE+BT vs UC: 2<br>BT vs UC: 2<br>AE vs UC: 3<br>AE+RE vs UC: 1<br>AE+BT vs AE: 1	AE+BT vs AE: 1 study	BT vs AE; BT vs AE+BT; BT vs AE+RE; AE+RE vs AE; AE+RE vs AE+BT	Moderately connected but still largely UC-centred; only one active-comparator edge is available.

Table S21.1. Continued

Outcome network	Nodes represented in Figure 2	Direct comparisons, n studies	Direct active-versus-active evidence	Indirect active-versus-active comparisons	Interpretation
FEV1%	AE+BT (n=35); BT (n=5); AE (n=12); UC (n=31)	AE+BT vs UC: 1<br>BT vs UC: 1<br>AE+BT vs AE: 1	AE+BT vs AE: 1 study	BT vs AE; BT vs AE+BT; other active comparisons not directly represented	Very sparse network with small participant numbers; active-comparator evidence is based on a single study.
Resting HR	AE+BT (n=53); BT (n=14); AE (n=31); AE+RE (n=4); UC (n=108)	AE+BT vs UC: 2<br>BT vs UC: 1<br>AE vs UC: 2<br>AE+RE vs UC: 1	None	AE+BT vs AE; AE+BT vs BT; AE+RE vs AE; AE+RE vs BT; BT vs AE	Star-shaped network centred on UC; active-versus-active comparisons are entirely indirect.
Maximum HR	AE+BT (n=53); BT (n=5); AE (n=37); AE+RE (n=16); UC (n=114)	AE+BT vs UC: 2<br>BT vs UC: 1<br>AE vs UC: 2<br>AE+RE vs UC: 2	None	AE+BT vs AE; AE+BT vs BT; AE+RE vs AE; AE+RE vs BT; BT vs AE	Star-shaped network centred on UC; no direct active-versus-active comparisons are available.

Note: AE, aerobic exercise; BT, breathing training; RE, resistance exercise; UC, usual care; HR, heart rate; 6MWD, 6-minute walk distance; NT-proBNP, N-terminal pro-B-type natriuretic peptide; FEV_1%, forced expiratory volume in 1 second percentage predicted. The table complements Figure 2 and summarises the distribution of direct evidence across plotted network edges. Direct active-versus-active evidence was limited to AE+BT vs AE in selected outcome networks; most other active-versus-active comparisons were estimated indirectly and should be interpreted cautiously.
